# A new chromosome-scale duck genome shows a major histocompatibility complex with several expanded multigene families

**DOI:** 10.1186/s12915-024-01817-0

**Published:** 2024-02-05

**Authors:** Jiaxiang Hu, Linfei Song, Mengfei Ning, Xinyu Niu, Mengying Han, Chuze Gao, Xingwei Feng, Han Cai, Te Li, Fangtao Li, Huifang Li, Daoqing Gong, Weitao Song, Long Liu, Juan Pu, Jinhua Liu, Jacqueline Smith, Honglei Sun, Yinhua Huang

**Affiliations:** 1 State Key Laboratory of Farm Animal Biotech Breeding, College of Biology Sciences, China Agricultural University, No.2 Yuan Ming Yuan West Road, Hai Dian District, Beijing, 100193 China; 2 Key Laboratory for Prevention and Control of Avian Influenza and Other Major Poultry Diseases, Ministry of Agriculture and Rural Affairs, College of Veterinary Medicine, China Agricultural University, No.2 Yuan Ming Yuan West Road, Hai Dian District, Beijing, 100193 China; 3 Jiangsu Institute of Poultry Science, Yangzhou, China; 4 College of Animal Science and Technology, Yangzhou University, Yangzhou, China; 5 The Roslin Institute and Royal (Dick) School of Veterinary Studies, University of Edinburgh, Midlothian, EH25 9RG UK

**Keywords:** Duck, Influenza resistance, Chromosome-scale genome, Adaptive immunity, MHC gene map

## Abstract

**Background:**

The duck (Anas platyrhynchos) is one of the principal natural hosts of influenza A virus (IAV), harbors almost all subtypes of IAVs and resists to many IAVs which cause extreme virulence in chicken and human. However, the response of duck’s adaptive immune system to IAV infection is poorly characterized due to lack of a detailed gene map of the major histocompatibility complex (MHC).

**Results:**

We herein reported a chromosome-scale Beijing duck assembly by integrating Nanopore, Bionano, and Hi-C data. This new reference genome SKLA1.0 covers 40 chromosomes, improves the contig N50 of the previous duck assembly with highest contiguity (ZJU1.0) of more than a 5.79-fold, surpasses the chicken and zebra finch references in sequence contiguity and contains a complete genomic map of the MHC. Our 3D MHC genomic map demonstrated that gene family arrangement in this region was primordial; however, families such as AnplMHCI, AnplMHCIIβ, AnplDMB, NKRL (NK cell receptor-like genes) and BTN underwent gene expansion events making this area complex. These gene families are distributed in two TADs and genes sharing the same TAD may work in a co-regulated model.

**Conclusions:**

These observations supported the hypothesis that duck’s adaptive immunity had been optimized with expanded and diversified key immune genes which might help duck to combat influenza virus. This work provided a high-quality Beijing duck genome for biological research and shed light on new strategies for AIV control.

**Supplementary Information:**

The online version contains supplementary material available at 10.1186/s12915-024-01817-0.

## Background

Newly emerging or re-emerging influenza caused by the influenza A virus (IAV) continue to pose global public health threats. IAVs are responsible for millions of severe cases and 290,000–650,000 deaths in human each year according to the World Health Organization. Ducks serve as the principal natural reservoir for IAVs and harbor all hemagglutinin (HA) and neuraminidase (NA) subtypes of IAVs with the exception of the H17N10 and H18N11 subtypes. Many IAVs including high pathogenic H5 subtype viruses circulate in ducks and cause little harm, while they are responsible for respiratory and systemic disease when transmitted to other hosts such as chicken and human [1, 2]. The long co-evolution between duck and IAVs has undoubtedly fine-tuned the host immune system to combat influenza virus. Previous studies have examined the non-major histocompatibility complex (non-MHC) elements such as β-defensins, type I interferon, RIG-I and pro-inflammatory cytokines to explain duck’s disease resistance strategies in response to IAV infection [3–6]. However, due to high gene density, GC content, and sequence diversity, the MHC associated with disease resistance is hard to assemble [7, 8], thus limits us to understand how ducks combat IAVs.

High-throughput sequencing technologies and traditional assembly tools have not enabled proper genomic draft of highly repetitive and GC-rich sequences, such as the MHC. Therefore, avian MHC gene maps tended to be constructed through sequencing of MHC-containing fosmids or BAC clones instead of de novo assembly [9]. The first avian genomic MHC map was the chicken minimal and essential one on chromosome 16. This map, spanning 92 kb and harboring 19 genes, was then extended to be 242 kb containing 46 genes [10, 11]. After that, four galliformes (Turkey, quail, golden pheasant, and black grouse) MHC-B regions were reported to be highly syntenic to that of chicken, except for expansion of a few gene families such as *BG*, *MHCIIB*, and *MHCIα* and inversion of gene loci such as *TAPBP*, *TAP1*, and *TAP2* [12–14]. In contrast, study of the MHC in waterfowl is limited, where two fragments of the duck MHC map were first published by Moon et al. and Ren et al. [15, 16]. Limited by the shortage of gene information in the duck MHC, only a few functional studies on duck MHC have been performed [16, 17]. Recently, the development of single-molecule sequencing (third-generation sequencing), such as Pacific Biosciences Single Molecule Real-Time sequencing or Oxford Nanopore sequencing, make it possible to have reads with lengths of hundreds of kilobases, thus largely improved the assembly quality of repetitive regions of genomes [18, 19].

Here we apply 95-fold Nanopore long reads, 117-fold 150bp paired-end Illumina genomic reads, 216-fold optical map reads and 234-fold PE150 Hi-C reads to generate a highly contiguous chromosome-scale Beijing duck reference genome (SKLA1.0) with a complete MHC genomic map. We use this high-quality genome to understand the evolution of innate and adaptive immune genes in duck and describe features relevant to resistance of influenza virus.

## Results

### The chromosome-scale genome assembly has high contiguity and completeness

**Fig. 1:**
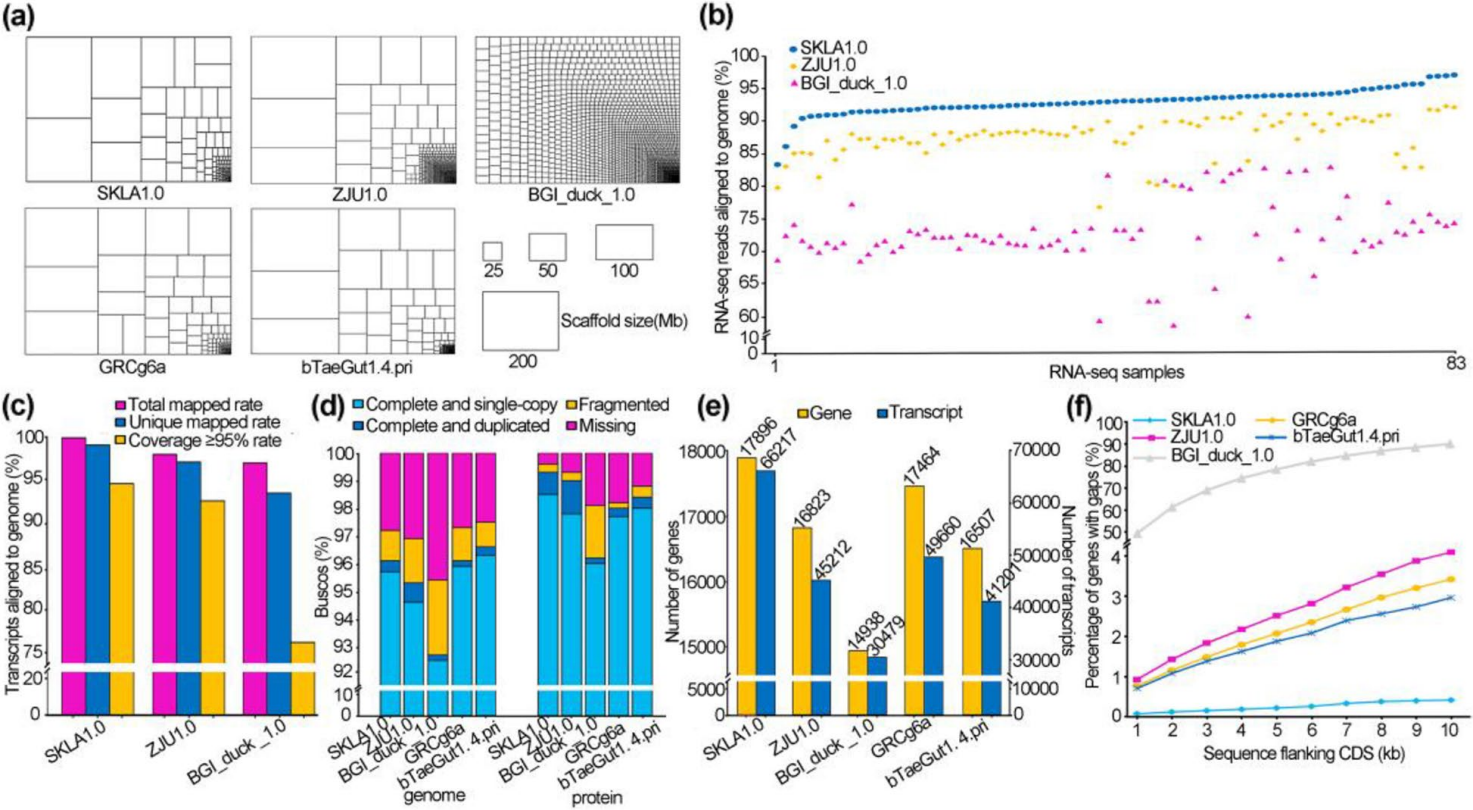
Comparison of genome quality among duck, chicken, and zebra finch. Assessment was carried out according to the following genome versions: duck SKLA1.0; our previous duck genome (BGI duck 1.0); the available duck genome with highest contiguity (ZJU1.0); two high-quality avian genomes (the chicken GRCg6a and zebra finch bTaeGut1.4.pri). **a** Treemaps of five genome assemblies scaled by contig length. **b** Mapping ratios of 83 RNA-seq data to three duck assemblies. **c** Mapping ratio of 1,625,932 transcripts to three duck assemblies. Transcripts were from de novo assembly of RNA-seq reads and corrected Pacbio Iso-seq reads. **d** Completeness of five assemblies and reference protein sets estimated by the BUSCO software. **e** Number of coding genes and transcripts annotated in the five assemblies. **f** Percentage of genes with gaps in flanking sequence

Before assembly, we estimated the genome heterozygosity of C18 duck and the heterozygosity is as low as 0.58% (Additional file 1: Table S1 and Additional file 2: Fig. S1-S3). We generated a high-quality duck genome sequence for Pekin duck (called duck hereafter), a native breed in China, using a hierarchical and hybrid approach. Using 71-fold normal and 24-fold ultra-long Nanopore reads, we assembled the duck genome into 151 contigs covering a total length of 1.22 Gb with a contig N50 of 32.81 Mb (Additional file 1: Table S2-S3). These 151 contigs were then polished with 912 million 150-bp Illumina pair-end reads, corrected and integrated with high-quality optimal maps (Additional file 1: Table S4-S5). This effort generated 69 scaffolds with a scaffold N50 of 72.53 Mb (Additional file 1: Table S6). A total of 274 Gb PE150 Hi-C data was used to order and orient duck scaffolds, correct mis-joined sections and merge overlaps, which generated 40 super-scaffolds (Additional file 1: Table S7). We further performed gap filling using 95-fold corrected Nanopore reads to remove gaps and generated the final duck assembly (SKLA1.0) representing 1.16 Gb of the genomic sequence, which is ~99.11% of the estimated genome size (Table 1). Since duck contains 80 chromosomes (diploid, 2n=80), we inferred that this duck assembly had covered all chromosomes except W (Additional file 1: Table S8). Moreover, we compared our SKLA1.0 assembly with our previous duck BGI_duck_1.0 assembly, the available duck assembly with highest contiguity (ZJU1.0) and two high-quality avian reference genomes (the chicken GRCg6a and zebra finch bTaeGut1.4.pri). These analyses indicated that the SKLA1.0 assembly represents a major improvement over the BGI_duck_1.0 and ZJU1.0 genomes in contiguity, completeness and chromosome size. The contiguity and completeness of SKLA1.0 is also higher than that of the zebra finch bTaeGut1.4.pri and the chicken GRCg6a (Fig. 1a–d and Table 1).

**Table 1.** Comparison of assembly contiguity statistics in three ducks, chicken and zebra finch genomes

Index	Peking duck SKLA1.0	Peking duck ZJU1.0	Peking duck BGI 1.0	Chicken GRCg6a	Zebra finch bTaeGut1.4.pri
Assembly size (Mb)	1159.66	1188.53	1105.04	1065.36	1056.27
Chromosome	40	33	0	34	41
Sequencing technology	Illumina Nanopore Ultra-long Bionano Hi-C	Illumina 10 X PacBio Bionano Hi-C	Illumina	PacBio	Arima Illumina PacBio HIFI Bionano Hi-C
Coverage (X)	117 Illumina 71 Nanopore 24 Ultra-long 222 Bionano 234 Hi-C	92 Illumina 142 10X 143 Pacbio 56 Bionano 82 Hi-C	64 Illumina	82 PacBio	242 Arima 134 Illumina 396 10X 321 Pacbio 335 HIFI 2281 Bionano
Assembler	Nextdenovo SOLVE Juicer 3D-DNA Juicebox	FALCON Scaff10X runBNG SALSA2	SOAPdenovo	FALCON	FALCON Scaff10X Solve SALSA2
Contig N50 (Mb)	32.90	5.68	0.026	17.66	8.96
Contig L50	9	57	11206	19	32
Max contig (Mb)	160.37	28.52	0.26	65.78	65.49
No. contigs	151	1161	227448	1403	551
Scaffold N50 (Mb)	76.57	76.27	1.23	20.79	70.98
Scaffold L50	5	5	268	12	6
No. scaffolds	134	756	78487	525	199

We annotated 17,896 duck coding genes using the Funannotate pipeline and the GETA pipeline together with a manual curation of key gene families (Additional file 1: Table S9-S10 and Additional file 6: Supplementary note). This duck gene set had more protein-coding genes (17,896:17,464:16,507:16,823:14,938), higher BUSCO score (99.3%:98.0%:98.4%:99%:96.2%), many more transcripts (66,217:49,660:41,201:45,212:30,479) and fewer gaps than equivalent gene sets of chicken, zebra finch and two ducks, respectively (Fig. 1d–f and Additional file 1: Table S11).

To determine whether evolution at the gene family level could account for different susceptibility of AIVs, we examined gene family difference between the principal natural hosts and incidental hosts of AIVs with one amphibian, three mammals and four birds (Additional file 2: Fig. S4). This effort showed that duck significantly expanded 25 and contracted 13 immune-related gene families when compared to the chicken (Additional file 1: Table S12). Interestingly, the cell fate determining protein mab21-related, complement C4-A-related, MHC class II-related, butyrophilin (BTN), death inducer-obliterator 1, MHC class I and C-type lectin superfamily members were significantly expanded, while the properdin, B-cell lymphoma 3 protein, zinc finger and BTB domain-containing protein 1 were significantly contracted in duck.

### Duck has an extensive and complex major histocompatibility complex

**Fig. 2:**
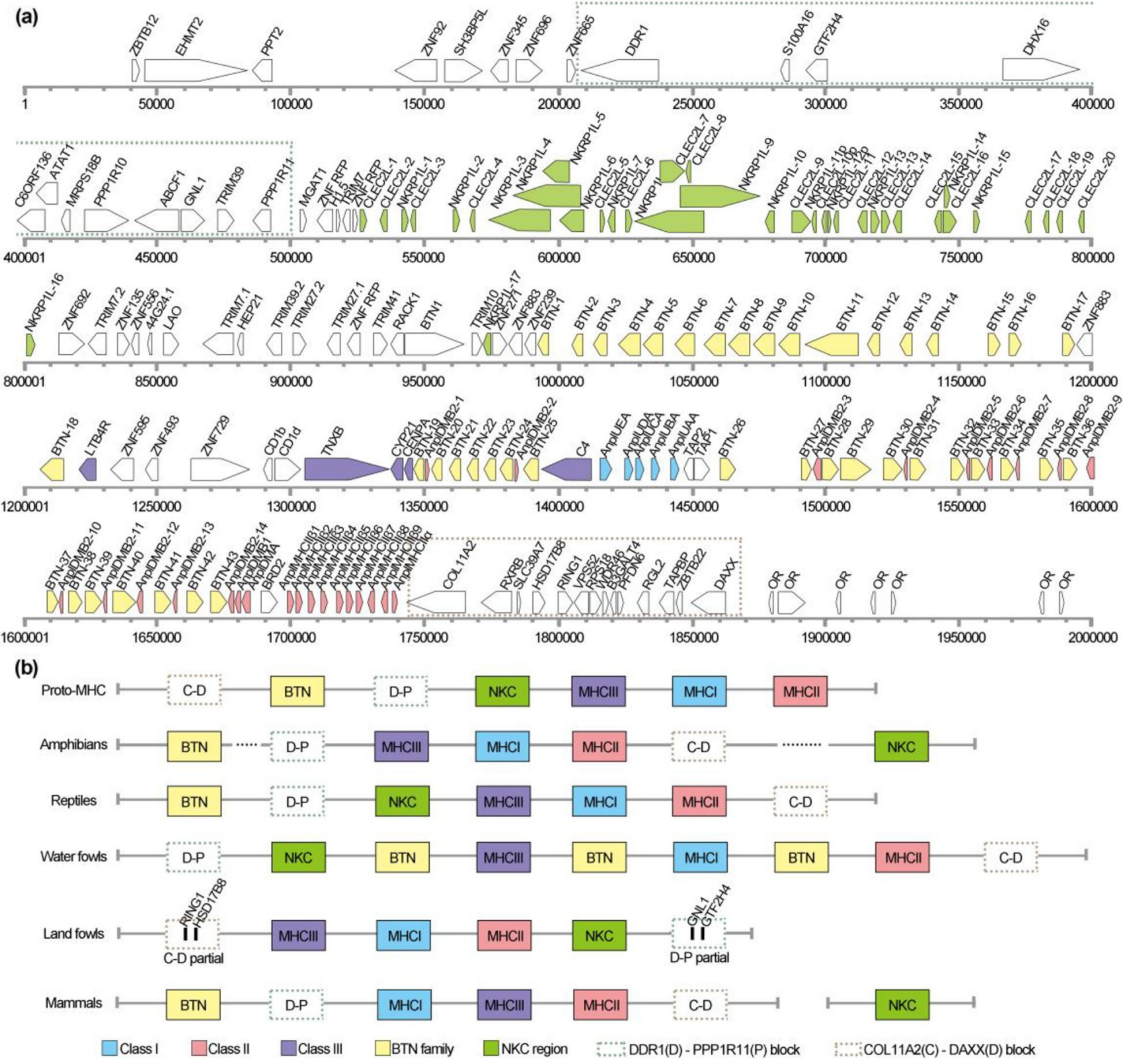
Landscape and comparison of SKLA1.0 MHC. **a** The arrangement order of genes in the duck MHC. The duck MHC is about 1.82 Mb and encodes 183 coding genes. *MHCI*, *MHCII*, *MHCIII*, *BTN*, and *NKC* gene families are colored in blue, pink, purple, yellow, and green respectively. The D-P and the C-D block are in green and brown dotted rectangle, respectively. **b** Comparison of MHC map in amphibians, reptiles, waterfowls, landfowls, and mammals. The MHC map includes seven blocks, namely MHCI, MHCII, MHCIII, BTN, NKC, D-P, and C-D. These blocks may not necessarily be completely separate, and in some species, one block may overlap with another block. The proto-MHC represents the ancient MHC map in vertebrates, which was inferred based on alignment of MHC gene maps from duck and 22 other species as well as previous ancient MHC map. Dash line denotes that BTN and NKC blocks are found in amphibian MHC, but the location is not consistent among these amphibian species

The MHC is crucial to initiate T cell-mediated immunity response to infection. Classical MHC class I molecules (MHCI) present peptides predominantly from proteins in the cytoplasm and nucleus to CD8^+^ T cells and classical MHC class II molecules (MHCII) present peptides predominantly from proteins in intracellular vesicles which are in contact with the extracellular space to CD4^+^ T cells [20–22]. However, function of MHC genes in duck is not well-characterized due to the lack of a complete MHC genomic map. We herein generate a 4.13-Mb contig (referred to as chromosome 17) containing the complete duck MHC genomic sequence. After annotation, we find that the duck MHC is about 1.82-Mb and encodes 183 gene loci (Fig. 2a and Additional file 3: Data S1).

Since MHC-B maps in galliformes share good synteny with each other, we compared duck MHC with these of two galliformes, namely chicken and quail. The data shows that duck MHC is more extensive and complex than the galliform MHCs. For chicken, its “minimal and essential” MHC only contains 41 loci within 0.28 Mb in the chicken GRCg6a reference (GCF_000002315.6) (Additional file 2: Fig. S5). Quail MHC map assembled from both BAC clones [14, 23] and NGS (NCBI genome version: GCF_001577835.2) indicated that the organization of gene families in quail is very similar to that of chicken MHC. We further verified sequence of 5 fragments in duck MHC using Sanger sequencing (Additional file 1: Table S13) and compared the duck MHC genomic map to those of seven amphibians, eight reptiles, five birds and two mammals (Fig. 2b and Additional file 1: Table S14-S16). This analysis indicates that the duck MHC map is of high quality and characterized as a primordial, expanded and complex region. Firstly, the duck MHC is organized as a “*MHCIII*-*MHCI*-*MHCII*” arrangement and has the conserved *ZNF692-TRIM10* subregion (Additional file 2: Fig. S5). Secondly, duck *MHCI* and *MHCII* genes are separated by duplicated *BTN* and *DMB* genes, with *MHCIII* genes flanked by tandemly duplicated *ZFN* and *BTN* genes (Fig. 2a). Thirdly, we defined the C-D block as a region between *COL11A2* (C, collagen type XI alpha 1) gene and *DAXX* (D, death domain associated protein) gene, which encompasses 14 genes (*COL11A2-RXRB-SLC39A7-HSD17B8-RING1-VPS52-RPS18-WDR46-B3GALT4-PFDN6-RGL2-TAPBP-ZBTB22-DAXX*). The C-D block located in the extended class II subregion of human MHC gene map. We defined the D-P block as a region between *DDR1* (D, discoidin domain receptor tyrosine kinase 1) gene and *PPP1R11* (P, protein phosphatase 1 regulatory inhibitor subunit 11) gene, which includes 12 genes (*DDR1-S100A16-GTF2H4-DHX16-C6ORF136-ATAT1-MRPS18B-PPP1R10-ABCF1-GNL1-RIM39-PPP1R11*). D-P block located in the Classical class I subregion of human MHC gene map (Additional file 1: Table S17). The duck MHC has retained the D-P block, BTN gene family, and C-D block, which are present in the human MHC, but are almost lost in chicken (Fig. 2b and Additional file 1: Table S17). Fourthly, the duck MHC is organized in a primordial pattern, similar to previously proposed primordial MHC organizations based on evolutionary analysis [24–26]. Moreover, duck MHC is similar to that of amphibians and reptiles. In addition, duck contains the ancient NKR framework of genes, which are adjacent to the MHC genes (Fig. 2b), as in the previously reported proto-MHC in ectotherms [25].

We further performed gene expression profile analysis and found that, compared to control individuals, 81 MHC genes showed significantly differential expression in ducks infected by H5N1 IAV, supporting the idea that many duck MHC genes were associated with host immune response to IAV infection (Additional file 3: Data S1-S6).

### Characteristics of duck MHCIA genes and TAPBP gene

The MHCI heterotrimers consist of an invariant light chain (β2M), a polymorphic heavy chain (MHCIα, encoded by *MHCIA*), and an antigen peptide and presents peptides through interacting with TAPBP (Tapasin). MHCI and peptide complexes evoke CTL (cytotoxic T cell) response by interacting with CD8α molecule and TCR (T cell receptor) [7]. The chicken dominantly expressed *MHCIA* (BF2) which was associated with disease susceptibility [27]. In contrast, duck has a significantly expanded *MHCIA* gene repertoire, where five *MHCIA* genes are expressed in four IAV target organs (lung, ileum, jejunum and duodenum) and one immune organ (spleen) (Additional file 2: Fig. S6).

**Fig. 3:**
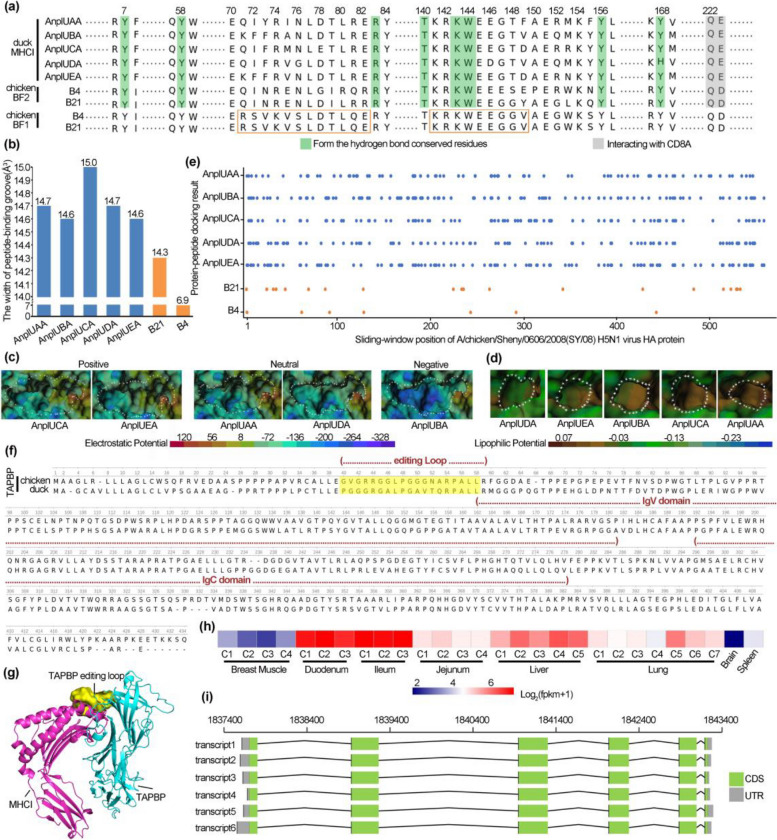
Characteristics of *MHCI* and *TAPBP* genes in duck. Duck and chicken had five and two *MHCI* genes respectively. “*B4” and “*B21” represent different chicken MHC haplotypes. Lung and plasma tissues of control and infected ducks at 12 h, 24 h, and 48 h post inoculation were collected (*n* = 5). **a** Comparison of duck MHCIα proteins to chicken ones. Conserved residues interacting with CD8A or antigen peptide are highlighted in gray and green, respectively. There is a potential inference that two motifs may interact with the NK cell receptor [28], and these two motifs are shown in the orange rectangle. **b** Opening size of the peptide-binding pocket of duck MHCIα proteins and chicken BF2 proteins. **c** Electrostatic potential of duck MHCIα proteins. Area circled by white dotted lines is the peptide-binding pocket. **d** Lipophilic potential of duck MHCIα proteins. Pocket B is circled by white dotted lines. **e** Peptide recognition spectrum of duck MHCIα and chicken BF2 proteins. Short peptides (ranging from 8–10 aa) were randomly extracted from HA protein sequence and were docked into the binding groove of MHCIα proteins. Dots represented peptides could be bound by MHCIα. **f** Sequence alignment and domain of duck and chicken TAPBP protein. **g** Predicted interacting model between duck MHCI and TAPBP. **h** Expression heatmap of duck TAPBP gene. C1~C7 represent the numbers of 7 ducks. Detailed sample information is in Additional file 3: Data S6. **i** Transcripts of duck TAPBP gene

Human HLA-C/E have a conserved motif which can interact with members of the KIR family (Killer cell immunoglobulin like receptor) on natural killer cells to regulate NK killing activity and there is a potential inference that two motifs in chicken BF1 may interact with the NK cell receptor [28–31]. It is hard to determine whether duck MHCIαs may function like human HLA-C/E and chicken BF1 since there are many variants in the key motifs (residues 71–82 and 141–149) (Fig. 3a and Additional file 2: Fig. S7) [15, 17, 28, 32–35]. According to previous researches on MHCIα proteins [15, 32–34], duck MHCIαs have two conserved sites with negative charged side chain (Q222 and E223) which interact with three conserved duck CD8A residues (homologous to S34, Y51 and S53 of human CD8A), four residues (R83, T140, K143, and W144) in pocket F, and four residues (Y7, Y58, Y156, and Y168) in pocket A which participate in the hydrogen bonding network (Additional file 2: Fig. S7) [15, 17, 28, 32–35]. The homologous residue 83 is R in non-mammalian vertebrates but Y in mammals, and the R83 residue may allow the C-terminus of peptide to extend beyond the peptide-binding groove [34]. In addition, like human HLA-A2 protein [35], duck MHCIαs has the conserved cys-cys-trp structural triads (Additional file 2, Fig. S7) [15, 17, 28, 32–35]. These observations suggested that all duck MHCIαs, except UCA missing a region of the α3 domain, might present peptides to CTL cells as chicken BF2 does (Fig. 3a and Additional file 2: Fig. S7) [15, 17, 28, 32–35].

The peptide repertoire presented by chicken BF2 is positively correlated with the opening size of its binding groove [36, 37]. Interestingly, duck MHCIαs are predicted to have large opening size of the peptide binding pockets (Fig. 3b, Additional file 1: Table S18 and Additional file 2: Fig. S8). Duck MHCIαs are divergent in the electrostatic potential of their peptide-binding groove and the lipophilic potential of their B pocket (Fig. 3c, d and Additional file 2: Fig. S9-12) [15]. These observations reminded us that a divergent duck MHCIαs repertoire might allow binding of more types of peptides to increase duck’s immune surveillance for new and dangerous pathogens. This hypothesis was supported by short peptide docking analyses which predicted that duck MHCIαs have a larger number (76 to 104) haemagglutinin binding peptides of A/chicken/Sheny/0606/2008 (SY/08) H5N1 than chicken BF2 haplotypes (B4: 7; B21: 20) (Fig. 3e).

Due to low genome quality, duck *TAPBP* gene is not annotated for a long period of time [38]. In this genome version, we annotated a duck *TAPBP* gene, which loaded MHCI complex with peptide and shared 64% amino acid identity with chicken TAPBP. Duck TAPBP protein contained two tandem Ig domains and had an editing loop blocking the F pocket of MHCIα to bind peptide (Fig. 3f, g). RNA-seq analysis indicated that duck *TAPBP* gene was well expressed in duodenum, ileum, and liver tissues (Fig. 3h). Moreover, alternative splicing analysis showed that duck *TAPBP* gene encoded six transcripts (Fig. 3i). At last, we extract the UTR region and predicted promoter sequences of duck *TAPBP* (Additional file 1: Table S19).

Researchers carried out a large amount of valuable works on duck MHC Class I genes. Moon et al. [15] find that duck *UAA* is the dominantly expressed class I molecule like chicken *BF2*, while other duck *MHCIA* genes (*UBA*, *UCA*, *UDA*, and *UEA*) were not found to be expressed, with defects in the promoters, coding sequence or 3′UTR. Chan et al. showed that the promoters of *UBA*, *UCA*, and *UEA* failed to drive a reporter gene in a heterologous cell line and that *UDA* was expressed only when the miRNA let-7 family was absent [39], and thus these four genes were either non-classical or pseudogenes. Previous works based on limited samples or a single cell line failed to consider the issue of allelic polymorphism [40, 41]. However, the highly allelic polymorphic nature of these genes makes their characteristics and function more complex: *UBA* has a very low expression at the RNA level; *UAA* is a predominantly expressed gene at the RNA level in most cases; expression of *UCA*, *UDA*, and *UEA* varies largely among ducks, in some individuals, one of them even has a comparable expression level with *UAA* (Additional file 3: Data S2-5). In our MHC map, *UCA* has a big deletion in the alpha 3 region, but that does not mean all ducks have a defective UCA gene like C18 duck used in this project. *UCA* genes from other researchers [15] and resequencing data of other ducks indicates that many ducks have a complete *UCA* gene. Gene variation analysis in this region indicates that five *MHCIA* genes and *TAPBP* genes are all polymorphic (Additional file 4: Data S7).

### Duck expanded both the classical MHCIIB genes and the non-classical MHCIIB genes

**Fig. 4:**
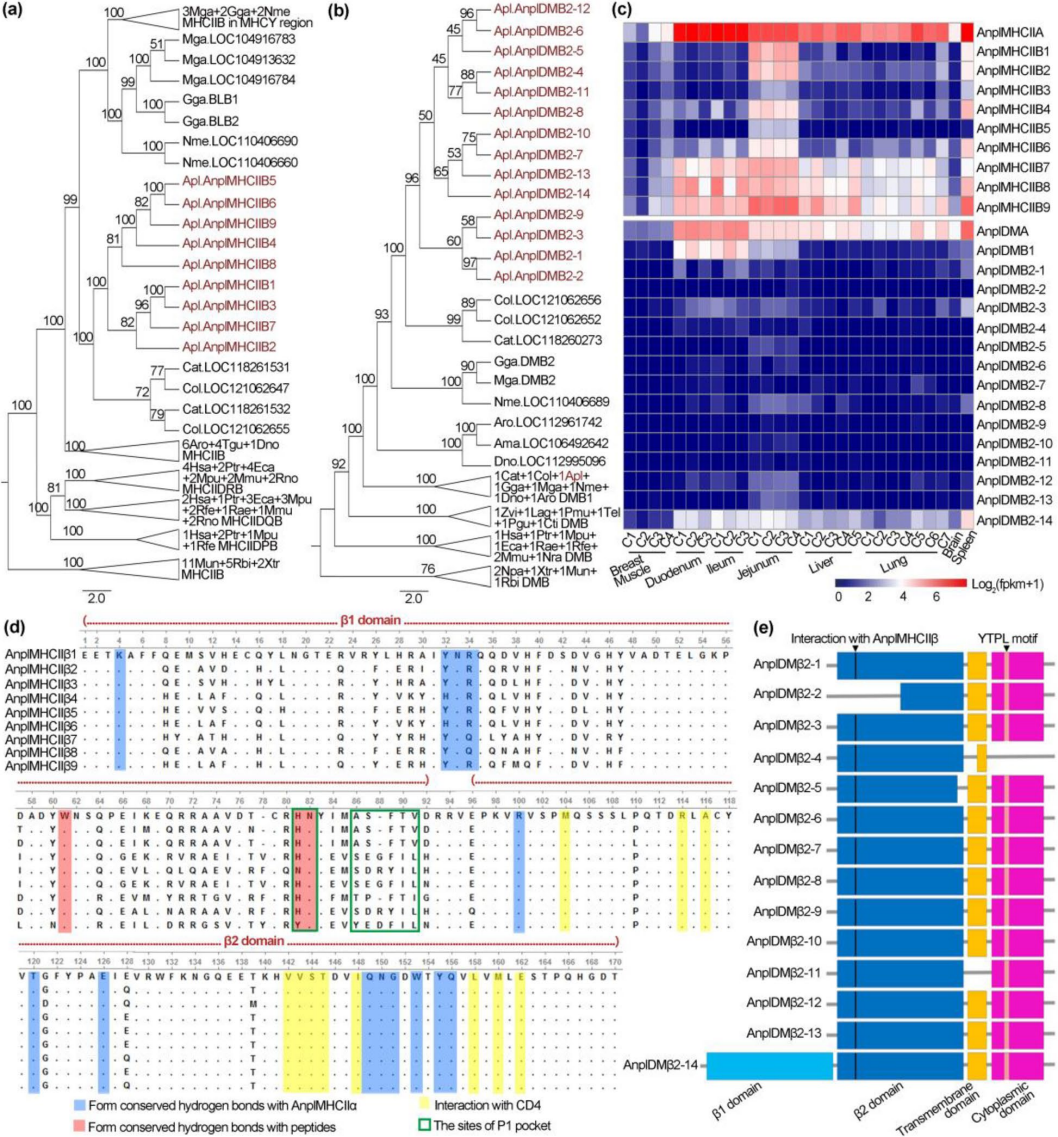
Expansion of *MHCIIB* and *DMB* genes in duck. **a,b** Maximum likelihood trees of *MHCIIB* (**a**) and *DMB* genes (**b**). Amphibian *MHCIIB* (classical MHCII β chain) and *DMB* (non-classical MHCII β chain) genes were set as outgroup. Numbers of the tree branches are bootstrap percentages with 1000 iterations. Abbreviated information on species is in Additional file 1: Table S20. **c** Expression of *MHCII* and *DM* genes in eight duck tissues. The MHCII heterodimer has two chains, namely MHCIIα (encoded by *MHCIIA*) and MHCIIβ (encoded by *MHCIIB*). The DM heterodimer consists of two chains, namely DMα (encoded by *DMA*) and DMβ (encoded by *DMB*). Detailed sample information is in Additional file 3: Data S6. **d** Multiple sequence alignment and domain of duck MHCIIβ proteins. “-” denotes gap, and “·” indicates the same amino acid as the first reference sequence. **e** Schematic of 14 DMβ2 proteins

The *MHCII* repertoire contains several distinct classical and non-classical *MHCII* isotypes, each with characteristic α and β chains encoded by A and B gene in mammals [42]. Interestingly, gene expansion event occurred in duck classical *MHCII and* non-classical *MHCII* gene families. Ducks contain one A gene (*AnplMHCIIA* and *AnplDMA*), but have a large number of B genes (nine *AnplMHCIIBs* and fifteen *AnplDMBs*) (Fig. 2a, Fig. 4a, b, and Additional file 2: Fig. S13-14). Expression pattern of these genes is different: *AnplMHCIIA* and *AnplDMA* are expressed highly in four target tissues of IAVs (ileum, jejunum, duodenum, and lung) and one immune organ (spleen); *AnplMHCIIBs* and *AnplDMB1* are expressed moderately and tissue-specifically; *AnplDMB2* genes have low expression baseline (Fig. 4c and Additional file 3: Data S2).

Chicken BLBs and human HLA-DR1B proteins contained two β domains, namely β1 and β2 domains. The β1 domain assembles a peptide-binding groove (PBG) with the α1 domain of MHCIIα to bind peptides, and researches in mammals indicated that the β2 domain contacts with the D1 domain of CD4 protein [43, 44]. The CD4 protein along with the T cell receptor that stimulate T cells to express cytokines and to directly stimulate B cells [43, 45]. Similarly, duck AnplMHCIIβ proteins contain β1 and β2 domains. According to previous researches on chicken BLBs and human HLA-DRB genes [20, 43, 45], we mapped key residues interacting with MHCIIα chain, CD4 protein and antigen peptide or forming P1 pocket to duck AnplMHCIIβ proteins (Fig. 4d). Multiple sequence alignment of the duck AnplMHCIIβ paralogs indicated that large numbers of variations were located in the β1 domain (except AnplMHCIIβ5 and AnplMHCIIβ9), especially in residues around the P1 pocket (Fig. 4d and Additional file 2: Fig. S15) [20, 43, 45]. The P1 pocket has been reported to affect peptide selection, stabilization of empty MHCII peptide-binding groove and DM-susceptibility in human [20, 46, 47]. More experiments are needed to validate the function of these duck AnplMHCIIβs paralogs. Among these variant sites, three contained loss-of-function mutations: H81N, like in AnplMHCIIβ5, disturbs the conserved H-bond formation between the β chain and the peptide backbone and impacts the ability of the exchange ligand [46]. G86Y, like in AnplMHCIIβ9, fills the P1 pocket, preventing conformational changes and greatly reduced responsiveness to DM heterodimer [47, 48]. Substitution of a neutral non-polar amino acid F89 by neutral polar amino acid Y89, as in AnplMHCIIβ5 and AnplMHCIIβ8, might influence stabilization of the P1 pocket (Fig. 4d and Additional file 2: Fig. S15) [20, 43, 45, 47]. In contrast, β2 domains are relatively monomorphic, including five variant sites and 70 identical sites. There are 11 identical AnplMHCIIβs sites and ten of them are conserved with the homologous sites of human HLA-DR1B or chicken BLB2. These sites in mammals are known to contact with the D1 domain of CD4 molecules [45].

Mammalian DMβ proteins contain four critical parts: the β1 domain interacting with DMα and MHCII (i.e. HLA-DR) to form a heterotetramer; the β2 domain stabilizing the overall topology of HLA-DM-HLA-DR1 heterotetramer; the transmembrane domain (TM) required for HLA-DM catalytic activity; the YTPL tyrosine-based endocytosis motif in the cytoplasmic domain required for DM sorting in endosomes [47, 49–51]. Surprisingly, only one (AnplDMβ2-14) of the fifteen AnplDMβs in duck has the above four parts and can efficiently catalyze peptide exchange like human HLA-DMβ and chicken DMβ2. However, thirteen DMβ2s cannot catalyze peptide exchange due to lack of the TM domain, the β1 domain, or YTPL motif (Fig. 4e and Additional file 2: Fig. S16).

### NK cell receptor NKRP1-like and its ligand-like genes are expanded in duck MHC

**Fig. 5:**
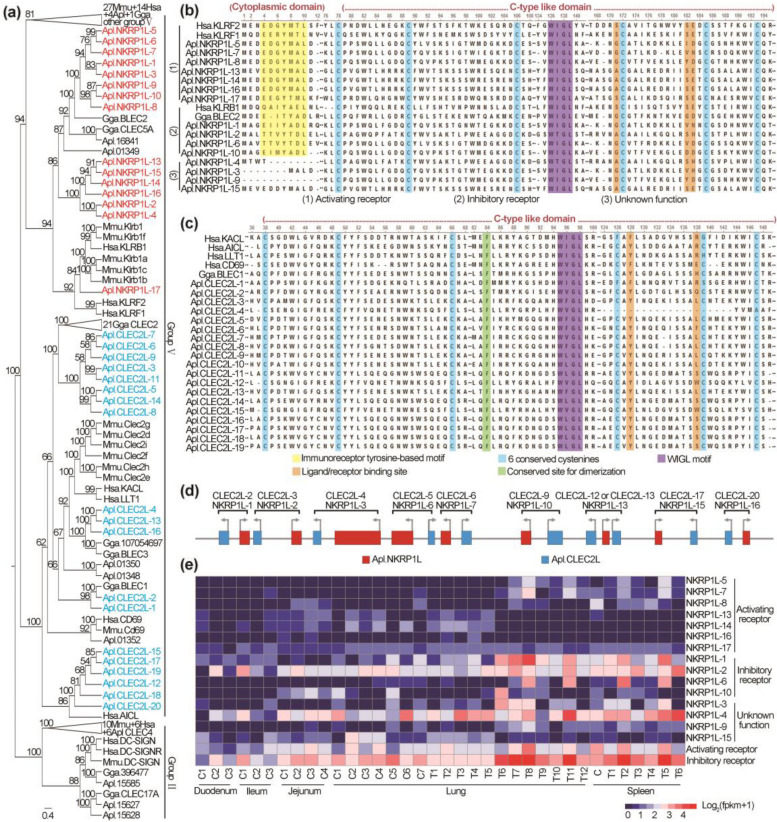
Expansion of *NKRP1-Like* genes and its ligand-like genes in duck MHC. **a** Maximum likelihood tree of *NKC-like* genes. Duck *NKC-like* genes clustered into two groups, namely groups II and V [54]. In group V, 15 duck *NKC-like genes* (referenced as *NKRP1L-1* to *NKRP1L-15*) grouped with mammalian *NKRP1* genes and 19 duck *NKC-like genes* (referenced as *CLEC2L-1* to *CLEC2L-19*) grouped with mammalian NKRP1 ligands. Numbers of the tree branches are bootstrap percentages with 1000 iterations. Abbreviated information on species is in Additional file 1: Table S20. **b,c** Partial multiple sequence alignment of NKC-like proteins in duck MHC (see Additional file 2: Fig. S18 and Additional file 5: Fig. S19 for details). “·” and “-” represent the same amino acids and gap, respectively. **d** Distribution of 9 pairs of *NKRP1-like/CLEC2-like* genes in the duck MHC. **e** Expression of *NKRP1-like* genes in ducks. Total gene expression of activating and inhibitory *NKRP1-like* genes is shown in the bottom two rows. Detailed sample information is in Additional file 1: Table S9

The mammalian NKC (natural kill gene complex) in the MHC paralogous region encoded many (22-50) C-type lectin-like NK cell receptors (CTLRs) to regulate NK cell activation [52]. Among them, the NKRP1 subfamily (including three members) modulate NK cell activation through interacting with NKC-encoded CLEC2 glycoproteins in human [53]. Interestingly, we found that both *NKRP1-like* genes (referred as *NKRP1L-1* to *NKRP1L-17*) and the ligands (referred to as *CLEC2L-1* to *CLEC2L-20*) were significantly expanded in duck MHC (Fig. 2a, Fig. 5a, and Additional file 2: Fig. S17). This was in sharp contrast to the case in chicken, which contains one pair of NKRP1-like receptor/ligand (*BN-K/B-lec*) and one other ligand (*CELC3*) in its MHC [55].

Mammalian *NKRP1* and *CLEC2* contain two critical domains: the cytoplasmic domain includes a tyrosine-based signaling motif, where the hem-ITAM (the hemi-immunoreceptor tyrosine-based activation motif) and the ITIM (the immunoreceptor tyrosine-based inhibition motifs)/ITSM (the immunoreceptor tyrosine-based switch motif) stimulates or inhibits phagocytosis, cytokine production and cytotoxicity, respectively [56]; the single extracellular C-type lectin domain (CTLD) creates a compact structure using a conserved “WI/TGL” motif to bind ligands/receptors [53].

Interestingly, duck *NKRP1-like* genes are remarkably diversified in the tyrosine-based signaling motif, where one contained function undefined hem-ITAM-like (DDYXXL) motif, three lacked the tyrosine-based motif, four contained an ITIM or ITSM motif, and seven had a consensus D/EGYXXL hem-ITAM motif which becomes phosphorylated and rapidly recruits SYK to mediate cytolysis of malignant cells (Fig. 5b and Additional file 2: Fig. S18) [53, 55, 56]. This is different from that human *NKRP1* genes which contain a hem-ITAM domain and the chicken *BN-K* gene which contains an ITIM domain.

For the CTLD domain, duck NKRP1-like proteins are conserved in the “WI/TGL” motif with six invariant cysteine residues forming the core of the extracellular C-lectin like domain (CTLD). However, they were found to be diversified at three sites (homologous to S171, S182, and E183 of human NKp65) which form a hydrogen-bond network with their CLEC2 ligands (Fig. 5b and Additional file 2: Fig. S18) [53, 55, 56]. This was matched by the diversity at the hydrogen-bond network site (homologous to R138 of human KACL) in duck CLEC2s ligands. Perhaps, having a multigene family of C-lectin NK cell receptor/ligand pairs in ducks (like mice) is a different strategy from having a single receptor/ligand gene with a lot of allelic polymorphisms in chickens. Detailed sequence analysis indicated that duck CLEC2s are conserved at the critical site for forming CLEC2 homodimer (homologous to F84 of human KACL) (Fig. 5c and Additional file 5: Fig. S19). Genomic structure showed that nine pairs of duck *NKRAP1-like / CLEC2* genes are next to each other like human *NKRP1A/LLT1*, *NKp80/AICL* and *NKp65/KACL* (Fig. 5d). Further prediction suggested that nine NKRP1-like / CLEC2 dimers are very similar to human NKRP1A/LLT1 (5J2S:A/4QKH:A) in their tertiary structures with small RMSD (the global root mean square deviation, 0.33–1.35Å) and high GMQE (global model quality estimation score, 0.66–0.84) (Additional file 1: Table S18). The total expression level of these inhibitory NK receptor-like genes is higher than that of the activating ones in duck (Fig. 5e).

### Expanded duck BTNs surround and co-regulate with MHCI and MHCII

**Fig. 6:**
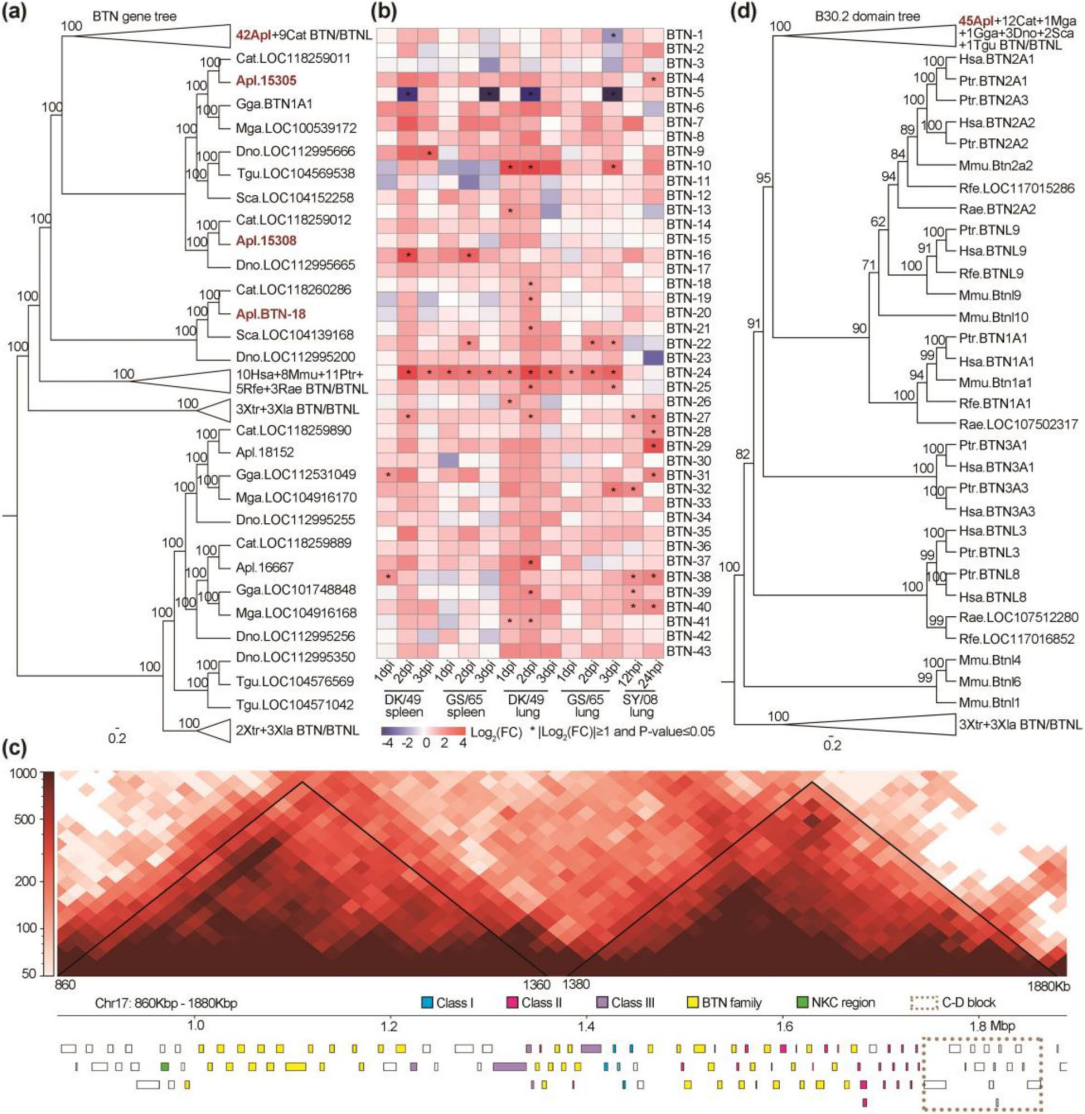
Expansion of the *BTN* gene family in duck. **a, d** Maximum likelihood trees based on full length of *BTN* genes (**a**) and the B30 domain of *BTN* genes (**d**). Numbers on branches are the tree bootstrap percentages with 1000 iterations. The full maximum likelihood tree is shown in Additional file 5: Fig. S20b. Abbreviated information on species is in Additional file 1: Table S20. **b** Gene expression of *BTN* genes in ducks infected by H5N1 virus at 12 hours (12hpi), 1 day (1dpi), 2 days (2dpi), and 3 days (3dpi) after inoculation. Influenza virus strains, tissues, and time point are marked under the map. Detailed sample information is in Additional file 3: Data S6. **c** Two topologically associated domains (TADs) of the duck MHC using Hi-C data from six individuals

*BTN* and *BTNL* (BTN-like) genes belong to the Ig superfamily and mostly compose of a membrane-distal IgV domain, a membrane-proximal IgC domain, a transmembrane region, and an intracellular C-terminal domain B30. Previous researches indicate that BTN and BTNL proteins inhibit activation and proliferation of αβ T cell such as CD4^+^ T cell and CD8^+^ T cell [57]. Recent researches revealed their new function in development [58] and activation of γδ T cell [59–61]. Unexpectedly, a large number (43) of *BTNs* were seen to surround *MHCI*, *MHCII*, *MHCIII*, *TAP1*, *TAP2*, and four *ZNFs* (zinc finger protein) genes in the duck MHC due to lineage-specific duplications (Fig. 2a, Fig. 6a and Additional file 5: Fig. S20a-b). Duck *BTNs* (except *BTN-10* and *BTN-12*) share similar domain structure with human BTNs and most of them express at high level in three IAV target tissues, namely ileum, jejunum, and duodenum (Additional file 5: Fig. S21). Twenty-two BTNs were significantly upregulated by 2.55- to 16.68-fold in lung or spleen, while two *BTNs* were significantly downregulated by 3.97- to 40.22-fold in lung of H5N1-virus infected ducks compared with controls (Fig. 6b).

It has been reported that Btn2a2−/− mice exhibited enhanced effector CD4^+^ and CD8^+^ T cell and Btn2A2 to be regulated by CIITA and RFX through a S-X-Y motif in the promoter [62, 63]. We extracted promoter sequences of all BTN genes in duck and submitted them to FIMO database [64] to predict motifs. There are 16 *BTN* genes (*BTN1, 4, 5, 8, 9, 12, 14, 20-22, 26, 37-38, 40-42*) with S-X-Y motif located in their promoter sequences (Additional file 5: Fig. S22 and Additional file 1: Table S21).

Using Hi-C data, we identified topologically associated domains (TADs) which serve as a structural scaffold for the establishment of regulatory landscape (RL). TADs represent a functionally privileged scale in chromosomes and genes within a TAD tend to be co-regulated [65, 66]. We found two TADs in duck MHC map and 18 *BTNs*, *MHCIs*, *MHCIIs*, *TAP1*, *TAP2*, and *TAPBP* were located in the second TAD (Fig. 6c). These 18 duck *BTN* genes might work in a co-regulating manner with MHCI-related and MHCII-related genes which play key roles in initiation of CD8^+^ T cell and CD4^+^ T cell. The location of these 18 *BTN* genes may contribute to regulating the activity of CD8^+^ T cell and CD4^+^ T cell.

BTN uses the B30.2 domain to recognize phosphor-antigen and then activate the γδ T cell [59]. This domain was first defined as a similar region in linear sequence of *BTN* and *TRIM* (tripartite motif-containing) genes [67–69]. Phylogenetic tree using the B30.2 domains showed that duck *BTNs* are clustered with a group of mammalian *BTN/BNTLs* genes (Fig. 6d and Additional file 5: Fig. S20b).

## Discussion

After challenging duck and chicken with same dose of H5N1 virus, duck has much lower death rate and mild symptoms relative to chicken. To explore the sharp difference of duck and chicken’s immune system, we de novo assemble a markedly improved Pekin duck genome draft SKLA1.0 with 40 chromosomes. We then compared the duck and chicken at the genome and transcriptome level and found that duck expanded classical *MHCI*, *NKRL*, *BTN*, classical *MHCII*, and non-classical *MHCII* genes.

### The arrangement of gene families may contribute to functional divergence

The class III region is sandwiched in between the class I and class II regions in mammals, but not in most non-mammalian vertebrates [24]. From the perspective of evolution, these two kinds of arrangement contribute to functional divergence. In chicken, *TAP* genes are next to *MHCI* genes they serve. Co-evolution will lead to a pathway with polymorphic interacting genes can only work effectively if the polymorphic genes are closely linked in the genome of a bird. In this circumstance, only *MHCI* gene nearest *TAP* gene expressed dominantly in most tissues and plays more important role than *MHCI* genes far from *TAP*. This model may have some drawbacks. For an individual, the antigens loaded and recognized by their *TAP* and *MHCI* are very limited, which makes them susceptible to infection. In human, *MHCI* and *TAP* genes are not adjacent and *TAP* genes pump a wide variety of peptides. Some of these peptides will be appropriate for any human *MHCI* proteins and each MHC haplotype will have a multigene family of class I molecules. Although duck *UAA* is next to *TAP* genes, there are four other *MHCI* genes which are not next to *TAP* genes. In chicken, two class I genes (*BF1* and *BF2*) flank the two *TAP* genes (*TAP1* and *TAP2*) and these two *MHCI* genes are highly polymorphic [70]. In duck, *MHCI* genes near or far from *TAP* genes all has a relatively low allelic polymorphism which is similar to human (Additional file 4: Data S7). Perhaps, the duck *MHCI* gene have evolved into an intermediate state between chicken *MHCI* genes and human *MHCI* genes.

The MHCIII region contains a cohort of genes include those involved in the activation of the complement system (*C2*, *BF*, *C4*), inflammation and cell stress (*TNFa*, *HSPA1A*, and *HSC70*), and Ig-SF members (*1C7*) [71, 72]. Some genes in this region have a functional relevance with the *MHCII* genes, for example antibodies can use complement to regulate antibody responses [73]. *HSC70* plays a central role in modulating antigen transport within cells to control MHC class II presentation during nutrient stress [71]. Human MHCII and MHCIII is adjacent to each other and may co-evolved to give rise to many complicate antiviral strategies. However, duck MHCII and MHCIII is separated by MHCI and the 3D MHC map also indicated that duck MHCII and MHCIII is hardly located in a same TAD. In this arrangement, MHCII may not co-evolve with MHCIII. Perhaps, the MHCII will co-evolve with BTN gene cluster since they are in the same TAD and thus may change antibody response.

### Besides resistance, tolerance may be another strategy employed by duck during IAV infection

Based on gene functional studies in human and chicken [30, 32, 74], these expanded *MHCI* and *MHCII* genes in duck may help to recognize invading pathogens and initiate immune cells such as CD8^+^ T cell and CD4^+^ T cell. Moreover, the expanded *BTN* and *NKR (like)* genes in duck may help to motivate another types of immune cells like γδ T cell and NK cell [53, 55, 69, 75]. Activation of these cells tend to increase the potency of immunity and inflammation. However, the inflammation is so weak in duck during IAV infection (Additional file 5: Fig. S23-26). In addition, antibody plays important roles during virus eradicating, but duck has weak antibody response comparing with chicken (Additional file 5: Fig. S27-28 and Additional file 1: Table S22) [76, 77], which is another confusing thing. There are two distinct host strategies to deal with an infection: antiviral resistance and disease tolerance [8, 78]. The former works by detection, neutralization, destruction, or expulsion of the pathogens, while the latter reduces the negative impact of infection on host fitness. From the point of resistance, we cannot explain the weak inflammation and weak antibody response, we then turn to explore whether there are some tolerance measures in duck.

The CD8 protein that interacts with classical class I molecules in mammals is a heterodimer of CD8α (encoded by *CD8A*) and CD8β (encoded by *CD8B*) to allow cytotoxicity by CTLs [79], while CD8αα homodimers in mice interact with the non-classical class I molecules called TL to deliver various signals in the intestine and thymus [80–83]. *CD8A1* is a variant gene of *CD8A*, which can not activate CD8^+^ T cells but contributes to inflammation [84]. Duck has much less members (1 VS 24) and low expression levels of *CD8A1* genes comparing with chicken, which may contribute to low inflammation level in duck (Additional file 1: Table S20, S23-S24 and Additional file 5: Fig. S29-30). Gene expansion gives rise to 13 fragmental *AnplDMB2s* which fail to edit the peptide and cooperatively present peptides with MHCII heterodimers due to lack of key domains. However, these fragmental AnplDMB2s may might negatively affect peptide presentation through forming defective DM dimer and MHCII-DM tetramer (Additional file 5: Fig. S31). Besides activating NKRP1-like receptors, duck also expanded four inhibitory NKRP1-like receptors, and the expression level of inhibitory NKRP1-like receptors is even higher than that of activating ones. These observations support the idea that ducks might use diverse inhibitory NKRP1-like receptors to inhibit NK activation (Fig. 5e). Besides activating γδ T cells, BTNs inhibit the proliferation and activity of αβ T cell, such as CD4^+^ T and CD8^+^ T cells [57, 62, 63].

These measures may not directly contribute to eliminating virus; however, they tend to protect the host from cytokine storm and too much tissue damage. A more reliable model in duck may be composed of both resistance and tolerance strategies (Additional file 5: Fig. S32).

## Conclusions

To understand the antiviral strategy of duck, we assembled a chromosome-level reference genome (SKLA1.0) and performed immune-pathological and transcriptomic analyses. This new duck reference genome covers 40 chromosomes with a contig N50 of 32.90 Mb which surpasses the two current model organism genomes (chicken and zebra finch) in sequence contiguity. We also successfully assembled the complete duck MHC and verified its accuracy. Moreover, we compared the duck MHC gene map to these of fish, amphibians, reptiles, land birds and mammals. This analysis indicated that the arrangement of gene families in duck MHC is primordial with diversified *AnplMHCI*, *AnplMHCIIβ*, *AnplDMB*, *NKRL*, and *BTN* genes. These expanded genes are tightly organized in their linear and 3D architecture, with 183 genes contained within a 1.82-Mb region and 111 of them being present in only two TADs. These important immune-related genes may help duck better resist to influenza viruses.

## Methods

### Genome assembly

Clean normal and ultra-long Nanopore reads were assembled into contigs using the NextDenovo software (https://github.com/Nextomics/NextDenovo, version 2.1-beta.0). Contigs were then polished three rounds using Illumina clean reads with the Nextpolish software (version 1.2.3) [85]. At the same time, the Bionano map was assembled with the SOLVE software (https://bionanogenomics.com/support/software-downloads, version 3.2.1) using clean BNX files and refined according to polished contigs. Subsequently, hybrid scaffolds were generated by integrating Bionano maps and polished contigs. Hybrid scaffolds were further assigned to chromosomes to develop the draft genome after polishing, splitting, sealing, and merging with clean Hi-C reads using the Trimmomatic (version 0.36) [86], the Juicer software (version 1.5) [87], and the 3d-DNA package (version 180922) [88] with default parameters. After that, the raw genome draft was manually refined according to contact matrices of Hi-C data using the Juicebox (version 1.13.01) [89]. Finally, three rounds of gap filling were performed on the refined genome draft using normal and ultra-long Nanopore reads with the Gapcloser software (version 0.56) [90] to produce the final high-quality chromosomal-level genome.

### Assembly and verification of MHC genomic sequence

The MHC represents one of the most polymorphic and complex regions in vertebrate genomes, with *MHCI*, *MHCII*, and *MHCIII* genes being extremely difficult to assemble due to a high level of repetitive and GC-rich sequence. We performed de novo assembly using 81X Nanopore reads and 9X ultra-long reads (Additional file 1: Table S2). This only produced five small fragments with MHC genes ranging from 100kb to 1Mb. To get a complete MHC map, we further sequenced more ultra-long reads (17X) (Additional file 1: Table S2). Using these three read datasets, we assemble a contig containing the complete MHC genomic sequence and named it as chromosome 17 (chr17).

We assessed the consensus accuracy of the MHC through comparing the chr17 map to the Bionano map constructed with 259.60 Gb BNX data using the SOLVE software (https://bionanogenomics.com/support/software-downloads, version 3.2.1). We found that the MHC genomic sequence on chr17 (0.50 to 2.09 Mb) was completely consistent with the Bionano maps (Additional file 5: Fig. S33). We further visualized Hi-C data for the MHC using the Juicebox (version 1.13.01) software [89] and found that this region had even coverage. This observation suggested that the MHC genomic sequence was of good quality (Additional file 5: Fig. S34). Moreover, we performed sequence alignment between a duck BAC (bacterial artificial chromosome) containing fragmented MHC sequences (AY885227.1) and our MHC sequence using the MAUVE software (version 2.3.1) with default parameters. This indicated that our duck MHC sequence was consistent with these available fragmented MHC sequences. At last, we selected the CLEC subregion of the MHC (containing 36 tandemly repeated genes) to design five primer pairs for PCR amplification (Additional file 1: Table S13). PCR products were purified according to the manufacturer protocols (OMEGA, Norcross, Georgia 30071 USA) and were sequenced using the Sanger sequence technology. This effort verified that the MHC genomic sequence was assembled correctly.

### Gene annotation

We prepared four data for genome annotation: data set 1—RNA-seq dataset from 83 samples; data set 2—1,318,378 transcripts derived from NGS sequencing; data set 3—307,554 full-length transcripts derived from Pacbio Iso-seq; data set 4—77,519 homologous proteins from duck (BGI_duck_1.0), chicken (GRCg6a), mouse (GRCm39), and human (GRCh38.p13). Gene prediction was started using the GETA pipeline (https://github.com/chenlianfu/geta, version 2.4.4) with default parameters by inputting data sets 1 and 4 (Additional file 6: Supplementary Note). Gene prediction was also performed using the Funannotate pipeline (https://github.com/nextgenusfs/funannotate, version 1.7.4) with data sets 2, 3, and 4. We then integrated the above annotated information and performed homologous protein blast to remove low-quality and redundant gene models. Finally, we added transcript information by performing three-round analyses using the PASA pipeline (https://github.com/PASApipeline/PASApipeline/wiki,version 2.4.1) with data sets 2 and 3.

### Annotation and synteny analysis of the MHC

Three kinds of gene annotation namely, de novo prediction, homology-based prediction and transcriptome-based prediction were carried out on chr17 data. We integrated all gene models, performed protein sequence alignment using the BLAST software with e-value < 1e−10 and carried out domain prediction using the INTERPROSCAN (https://www.ebi.ac.uk/interpro/about/interproscan/) with defaults. We further manually removed redundant genes and those without conserved domains of homologous genes. MHC gene information was then collected from amphibians, reptiles, water fowls, land birds, and mammals from the NCBI Gene database to perform synteny analysis. According to previous studies and MHC gene maps [25, 91], we divided the MHC into seven conserved blocks referred as the D-P block, NKC subregion, BTN gene family, MHCIII gene family, MHCI gene family, MHCII gene family, and C-D block (Additional file 1: Table S14-S16). We compared the duck MHC gene map to those species to characterize the duck MHC.

### Gene expansion and contraction analysis

Protein sequences of seven species (chicken, zebra finch, emu, Egyptian rousette, greater horseshoe bat, human, and tropical clawed frog) retrieved from the NCBI together with protein sequences in SKLA1.0 were grouped using the OrthoMCL pipeline [92]. Gene group ID was collected by uploading human and chicken protein ID to the PANTHER database (http://www.pantherdb.org/) and groups with the same PATHER family ID were combined. This produced a total of 6606 gene families including 1800 single-copy gene families and 4806 multi-copy gene families. We then performed multiple sequence alignments using the Prank (version 14063) software, constructed maximum-likelihood trees using the IQ-TREE software (version 1.6.5) [93], viewed the resulting phylogenetic tree using the Figtree software (http://tree.bio.ed.ac.uk/software/figtree, version 1.42) and detected gene expansion/contraction with multiple-cope gene families using the CAFÉ (version 4.2.1) software [94] with default parameters.

### Structure prediction, pocket size calculation, and molecular docking

Protein templates were searched using the SWISS-MODEL website. Structures of duck MHCIα (UAA to UEA) were made by point mutation and optimization by the Discovery Studio software (version 2019) according to a template (PDB: 5GJX). Structure of chicken BF2 proteins were downloaded from the PDB database [32, 36, 95]. Structures of other protein were predicted according to templates using the I-TASSER website (https://zhanglab. ccmb.med.umich.edu/I-TASSER/) (Additional file 1: Table S18). The opening size of the active pocket was calculated using Pymol software (https://pymol.org/2/, version 4.2.0). HA protein sequence of A/chicken/Sheny/0606/2008 (SY/08) H5N1 virus was divided into peptides in silico according to the motif length reported in literatures [17]. Peptides were docked into peptide-binding pocket of MHCIα protein structures using the GalaxyPepDock website (https://galaxy.seoklab.org/cgibin/submit.cgi?type=PEPDOCK) and docking models were filtered according to interaction between B and F pockets of MHCI and peptide. Electrostatic potential (EP) and lipophilic potential (LP) maps were estimated using the MOLCAD program in the SYBYL (version X2.1.1) software.

### Promoter prediction for TAPBP gene

Gene promoter region is reported between the 2000bp upstream of the transcription start site and the translation start site (ATG). We choose the region of chr17:1843226-1845294 for promoter prediction using the BDGP with the defaults: Neural Network Promoter Prediction website (https://www.fruitfly.org/seq_tools/promoter.html).

### Identification of topologically associated domain (TAD)

Topologically associated domains (TAD) represent a major type of chromatin organization, with sizes ranging from tens of kilobases to several megabases, and they are conserved among species. TADs are characterized by pronounced long-range associations between loci located in the same domain, but with less frequent interactions between loci located in adjacent domains. Thus, TADs have two basic features: (1) self-association of regions within the TAD; (2) insulation between regions in neighboring TADs. Different methods of identifying TADs can be employed according to the above features of TADs, with a low number of interactions at TAD boundaries and higher numbers inside TADs [96–98]. Here, we used the insulation square analysis method to call TADs.

Hi-C reads from the duck sample sequenced in this study were mapped to our duck genome SKLA1.0, processed and iteratively corrected using the HiC-Pro software (version 2.11.1) [99]. We then used a perl script Cworld (https://github.com/dekkerlab/cworld-dekker, 0.0.1) matrix2insulation.pl to detect TAD boundaries at 20 kb resolution using the Hi-C data. Briefly, we calculated mean interaction across each bin by sliding a 400 kb × 400 kb (20 bins X 20 bins) square along the matrix diagonal and estimated insulation scores for all 20-kb diagonal bins by quantifying across each chromosome. These 20-kb valleys/minima of insulation score were defined as the TAD boundary. All boundaries with a boundary strength < 0.1 were discarded. Regions outside of the boundaries were then extracted using the inslution2tads.pl script and were defined as TADs. TAD maps were generated using the HiCExplorer (version 3.6) [100].

### Viral infection

A wild-type highly pathogenic H5N1 avian influenza virus, A/chicken/Sheny/0606/2008 (SY/08, clade 7), isolated from cloacal swabs of chickens and stored in −80°C in a biosecurity level 2+ laboratory approved by China Agricultural University was used in this study [101]. The SY/08 virus was propagated in 10-day-old fertilized chicken eggs with a total volume of 0.1–0.3 mL (Beijing Merial Vital Laboratory Animal Technology) at 37°C for 48–72 h and allantoic fluids were harvested and HA inhibition (HI) tests performed using a panel of reference sera [101]. Positive allantoic fluids were estimated for viral titer by counting egg infectious dose (EID_50_) individuals using the Reed and Muench method [102]. The SY/08 virus with titers up to 10^8.5^EID_50_ was used for the following animal infection study.

Two groups of 24-day-old specific pathogen-free (SPF) Shaoxing ducks (Vital River Laboratory, Beijing, China) were inoculated intranasally with 600μl 10^8.5^EID_50_ of SY/08 H5N1 virus or PBS by dripping into the trachea. Lung tissues and blood of ducks (*n*=3 for each group and point) were collected on hours 12, 24, and 48 post infection respectively. Same experiments were also carried out using chickens.

### RNA sequencing

Total RNA was extracted from about 100 mg of each lung tissue using the Qiagen RNeasy kit and RNA samples having an RNA integrity number (RIN) of ≥ 8.9 and a ratio of 28S:18S rRNA of > 1.0 were used to construct cDNA libraries according to the manufacturer’s instruction. They were then sequenced on the HiSeq 4000 System (TruSeq SBS KIT-HS V3, Illumina). Adaptor sequences and low-quality reads (Q value < 20) were filtered from raw RNA-seq data using Trimmomatic software (version 0.33) [86]. Clean reads were aligned to our duck genome SKLA1.0 using the HISAT2 (version v2.1.0) software [103] with default parameters. Multi-mapping reads were removed and only unique mapped reads were used to count gene expression level with the featureCounts script in the Subread package (http://subread.sourceforge.net/, version v2.0.0) under the setting of “-g gene”. Counts of uniquely mapped reads were used to calculate FPKM (fragments per kilobase million) values using the R package, edgeR (version 3.2) with default parameters. Differential expressed genes were determined with the DESeq2 algorithm [104] under thresholds of *P*-value ≤ 0.05 and |log_2_ (fold change)| ≥ 1. Gene expression profiles were viewed using heatmaps generated by the R package ggplot2 and pheatmap.

### Immuno-pathological analysis

Lung tissues were fixed in 4% paraformaldehyde for 24 h, processed for paraffin embedding and sectioned at 4 μm. Lung sections were stained with hematoxylin and eosin (H&E). The other lung sections were immune-histochemically stained as follows: subjected to antigen retrieval by heating the slides to 95°C for 20 min in 0.01 M citrate buffer and blocked with serum; sections were labeled with a rabbit polyclonal NP antibody (Abcam) overnight at 4°C, followed by incubation with goat anti-mouse IgG biotin-conjugated affinity-purified antibody for 1h at 37°C. Immune complexes were visualized using the diaminobenzidine-tetrahydrochloride (ZSGB-BIO, Beijing, China).

After 12, 24, and 48 h post infection, whole bloods were collected from ducks infected by the SY/08 H5N1 virus and from control individual. Serum samples were isolated and tested for cytokine levels using five duck or chicken ELISA kits (Interlekin 4 (IL-4), Interlekin 6 (IL-6), Interlekin 8 (IL-8), interferon-γ (IFN-γ), and tumor necrosis factor-α (TNF-α)) from ELISA LAB (Wuhan, China). The microelisa stripplate provided in the kit had been pre-coated with an antibody specific to chicken/duck IL-4, IL-6, IL-8, IFN-γ, and TNF-α. Standards or samples were added to the Microelisa Stripplate wells to bind specific antibody and were then incubated with a horseradish peroxidase (HRP)-conjugated antibody specific for chicken/duck IL-4, IL-6, IL-8, IFN-γ, and TNF-α. Microelisa Stripplate wells were then washed using the TMB substrate solution, colored by addition of stop solution and OD measured at a wavelength of 450 nm. We further generated standard curves according to ODs of known concentrations (180ng/μL, 90 ng/μL, 60 ng/μL, 30 ng/μL, 15 ng/μL, 7.5 ng/μL) of duck or chicken IL-4, IL-6, IL-8, IFN-γ, and TNF-α and determined duck or chicken cytokine levels using a standard curve with *r*^2^ > 0.99.

MPO assay (NJJCBIO, A044-1-1) were performed according to the manufactures’ instructions. Lung specimens (10 mg) collected from ducks infected with the SY/08 H5N1 virus and from control individuals were homogenized in 190 μL homogenate medium. Suspension was heated for 15 min and then incubated with chromogenic agent for 30 min MPO activity in the solution was quantified by the microplate reader (TECAN GENios). MPO experiments were also carried out using chickens under the same conditions

### Generation of a recombinant attenuated H5N1 virus and antibody test

A recombinant attenuated SY08ΔHA H5N1 virus, which expressed a mutated HA protein containing an amino acid deletion (G325) in the HA cleavage site (HACS) region along with the seven other SY/08 viral proteins, was constructed by reverse genetics as described previously [101]. The SY08ΔHA virus was verified by the Sanger sequencing method and propagated in 10-day-old specific pathogen-free (SPF) chicken embryos. Two groups of 67-day-old SPF Shaoxing ducks were inoculated intranasally with 600 μL of 10^8.5^EID_50_ SY08ΔHA H5N1 virus or PBS by dripping into the trachea. Serum was collected from 10 individuals in each group and antibody titer was tested using hemagglutination inhibition (HI) assays after day 5, 7, 9, 12, and 14 post inoculation.

Spleen tissues of 10 ducks from each group were collected after days 7 and 14 post inoculation and total RNA was extracted using Trizol (Invitrogen, Rockvile, MD, USA). cDNA was generated using the high-capacity cDNA reverse transcription kit (Invitrogen, Rockvile, MD, USA). Quantitative PCR was performed to quantify gene expression based on primers in Additional file 1: Table S22. Differential expression between samples was calculated using the 2^−ΔΔCt^ method and was normalized to the expression level of *GAPDH* gene (internal reference gene). The antibody test experiments were also carried out using chickens under the same condition.

### Supplementary Information

**Additional file 1:** Table S1-Table S24. Table S1 - [Statistics of clean Illumina genomic data]. Table S2 - [Statistics of normal and Ultra-long Nanopore reads]. Table S3 - [Statistics of contig length assembled with Nanopore reads]. Table S4 - [Statistics of contig length assembled with Nanopore reads after polishing]. Table S5 - [Statistics of Bionano BNX reads]. Table S6 - [Statistics of scaffolds generated from Bionano and Nanopore reads]. Table S7 - [Statistics of Hi-C reads]. Table S8 – [Chromosome length of chicken (GRCg6a) and duck (SKLA1.0) genome]. Table S9 - [RNA-seq data for assessing genome quality, gene annotation]. Table S10 – [Chromosome information of the duck SKLA1.0 genome]. Table S11 – [Distribution of transcript annotated in the BGI 1.0, SKLA1.0, ZJU1.0, GRCg6 and bTaeGut1.4.pri genome]. Table S12 – [Expanded/contracted immune-related gene families in duck]. Table S13 – [Primer sequences used for PCR amplification of duck NKC region in the MHC]. Table 14 – [Information on conserved blocks in amphibian MHC]. Table 15 – [Information on conserved blocks in reptilian MHC]. Table S16 - [Information on conserved blocks in water fowl, land fowl and mammals MHC map]. Table S17 - [Gene information in D-P and C-D blocks]. Table S18 – [RMSD and GMQE values of predicted structures of duck MHC genes to templates]. Table S19 – [Promoter of the duck TAPBP gene]. Table S20 – [Species used in the maximum likelihood tree]. Table S21 - [S-X-Y motifs in the promoter of BTN genes]. Table S22 – [Primer sequences used for the quantitative PCR analysis]. Table S23 – [Expression of CD8A and CD8A1 genes in duck and chicken spleen tissue]. Table S24 – [Expression of CD8A and CD8A1 genes in duck and chicken lung tissues at 24hpi]. 

**Additional file 2:** Figure S1- Figure S18. Fig. S1 – [Photo of C18 duck]. Fig. S2 - [17-mer distribution curve]. Fig. S3 - [K-mer depth distribution curve]. Fig. S4 – [Gene expansion and contraction across a phylogenetic tree of eight species]. Fig. S5 – [Conserved synteny of MHC gene map in duck and chicken]. Fig. S6 – [Expressional profile of duck MHCIα genes in eight tissues]. Fig. S7 – [Multiple sequence alignment of MHCIα proteins]. Fig. S8 – [Opening size of the peptide-binding pocket of duck and chicken MHCIα proteins]. Fig. S9 – [Electrostatic potential of peptide-binding pocket of duck and chicken MHCIα proteins]. Fig. S10 – [Lipophilic potential of pocket B of duck and chicken MHCIα proteins]. Fig. S11 – [Electrostatic potential of the peptide-binding pocket of MHCIα proteins in another two ducks]. Fig. S12 – [Lipophilic potential of pocket B of MHCIα proteins in another two ducks]. Fig. S13 – [Maximum likelihood (ML) tree of classical major histocompatibility complex β chain (MHCIIβ) genes]. Fig. S14 – [Expansion of non-classical MHCIIβ (DMB) genes in duck]. Fig. S15 – [Multiple sequence alignment of classical MHCIIβ proteins]. Fig. S16 – [Multiple sequence alignment of DMB proteins]. Fig. S17 – [Maximum likelihood tree of NKC genes]. Fig. S18 – [Multiple sequence alignment of duck, chicken and human NKRP1 genes].

**Additional file 3:** Expression of genes in the MHC. Data S1-Data S6. Data S1 – [Detailed information on protein-coding genes in the MHC region]. Data S2 – [Gene expression level of 8 tissues sampled from 28 ducks]. Data S3 –[Differentially expressed genes of the MHC region in duck lung tissues at 12h and 24h post H5N1 infection]. Data S4 – [Differentially expressed genes of the MHC region in lung and spleen post DK/49 H5N1 infection]. Data S5 – [Differentially expressed genes of the MHC region in lung and spleen post GS/65 H5N1 infection]. Data S6 – [information of ducks used in RNA-seq analysis].

**Additional file 4:** Polymorphism analysis. Data S7. Data S7 – [Variation analysis of MHCI, DM and TAPBP genes].

**Additional file 5:** Figure S19- Figure S34. Fig. S19 – [Multiple sequence alignment of duck, chicken and human CLEC2 genes]. Fig. S20 – [Maximum likelihood (ML) tree of BTN genes]. Fig. S21 – [Expression profiles of duck BTN gene in eight tissues]. Fig. S22 – [Multiple sequence alignment of duck BTN proteins]. Fig. S23 – [Histopathological images of lung tissues from duck and chicken]. Fig. S24 – [Inflammatory cell infiltration in lung tissues of duck and chicken]. Fig. S25 – [Protein level of IFN-γ, IL-4, IL-6, IL-8 and TNF-α in duck (Top) and chicken (Bottom) plasma]. Fig. S26 – [Expressional profiles of inflammation-related genes in lung tissues of duck and chicken infected by A/chicken/Sheny/0606/2008 (SY/08) H5N1 virus]. Fig. S27 – [Antibody titer in ducks and chickens infected by the recombinant attenuated SY08ΔHA H5N1 virus]. Fig. S28 – [Expression of BCL6 and AICDA genes in spleen of ducks and chickens infected by the recombinant attenuated SY08ΔHA H5N1 virus and in control individuals]. Fig. S29 – [Multiple sequence alignment of CD8A proteins]. Fig. S30 – [Maximum likelihood tree of CD8As genes]. Fig. S31 – [Predicted structures of duck DM heterodimers]. Fig. 32 – [A proposed defense model to avian influenza virus in duck]. Fig. S33 – [Comparison of chromosome 17 (chr17) cmap and Bionano cmap]. Fig. S34 – [Hi-C matrix map of chromosome 17].

**Additional file 6:** Detailed materials and methods.

## Data Availability

The dataset(s) supporting the conclusions of this article are available. Six kinds of raw datasets were generated including Illumina genomic reads, RNA-seq reads, normal nanopore reads, ultra-long reads, Bionano BNX reads, and Hi-C reads. All datasets have been deposited in the Sequence Read Archive dataset with NCBI BioProject accession PRJNA792297. Normal and untra-long Nanopore data can be found under accession numbers SRR18156401 and SRR18159141. Whole-genome resequencing data are under accession numbers SRR18178819 and SRR18186809. Hi-C data from liver tissue were under accession numbers SRR19595116 - SRR19595119. Bionano map can be found under accession SUPPF_0000004299. RNA-seq data of lung tissues from ducks in treatment (infected with the SY/08 H5N1 virus) and control (infected with PBS) groups at 12 and 24 h post infection are under accession numbers SRR18934916 - SRR18934927. All data sets and research materials are available by contacting the corresponding author.

## Abbreviations

IAVs: Influenza A viruses
MHC: Major histocompatibility complex
Anpl: Anas platyrhynchos
BTN: Butyrophilin
MHCI/MHCII/MHCIII Class I/II/III: major histocompatibility complex
MHCIα: Alpha chain of the class I major histocompatibility complex
MHCIIβ: Beta chain of the class I major histocompatibility complex
AnplUAA/AnplUBA/AnplUCA/AnplUDA/AnplUEA: The class I major histocompatibility complex gene names for alpha chains at the protein level in duck
BF1/BF2: Alpha chain of class I major histocompatibility complex genes in chicken
KIR: Killer cell immunoglobulin like receptor
DMA/DMB: The non-classical class II major histocompatibility complex gene names for alpha/beta chains at the protein level
MHCIIα/MHCIIβ: Alpha/beta chain of the classical class II major histocompatibility complex molecules
CLEC: C type lectin gene
CTLD: C-lectin like domain
TADs: Topologically associated domains
NKRL: Nature killer cell receptor-like gene
