# Supplementary material for: A new chromosome-scale duck genome shows a major histocompatibility complex with several expanded multigene families
Source: BMC Biol. 2024 Feb 5;22:31. doi: 10.1186/s12915-024-01817-0 (PMC10845735; doi:10.1186/s12915-024-01817-0)
Supplement: Supplementary file 1 — Additional file 1: Table S1-Table S24. Table S1 - [Statistics of clean Illumina genomic data]. Table S2 - [Statistics of normal and Ultra-long Nanopore reads]. Table S3 - [Statistics of contig length assembled with Nanopore reads]. Table S4 - [Statistics of contig length assembled with Nanopore reads after polishing]. Table S5 - [Statistics of Bionano BNX reads]. Table S6 - [Statistics of scaffolds generated from Bionano and Nanopore reads]. Table S7 - [Statistics of Hi-C reads]. Table S8 – [Chromosome length of chicken (GRCg6a) and duck (SKLA1.0) genome]. Table S9 - [RNA-seq data for assessing genome quality, gene annotation]. Table S10 – [Chromosome information of the duck SKLA1.0 genome]. Table S11 – [Distribution of transcript annotated in the BGI 1.0, SKLA1.0, ZJU1.0, GRCg6 and bTaeGut1.4.pri genome]. Table S12 – [Expanded/contracted immune-related gene families in duck]. Table S13 – [Primer sequences used for PCR amplification of duck NKC region in the MHC]. Table 14 – [Information on conserved blocks in amphibian MHC]. Table 15 – [Information on conserved blocks in reptilian MHC]. Table S16 - [Information on conserved blocks in water fowl, land fowl and mammals MHC map]. Table S17 - [Gene information in D-P and C-D blocks]. Table S18 – [RMSD and GMQE values of predicted structures of duck MHC genes to templates]. Table S19 – [Promoter of the duck TAPBP gene]. Table S20 – [Species used in the maximum likelihood tree]. Table S21 - [S-X-Y motifs in the promoter of BTN genes]. Table S22 – [Primer sequences used for the quantitative PCR analysis]. Table S23 – [Expression of CD8A and CD8A1 genes in duck and chicken spleen tissue]. Table S24 – [Expression of CD8A and CD8A1 genes in duck and chicken lung tissues at 24hpi]. [file 12915_2024_1817_MOESM1_ESM.docx]

**Supplementary Tables**

**Table S1** Statistics of clean Illumina genomic data

| Paired-end libraries (bp) | Paired-end insert size  (bp) | Average Read length (bp) | Number of reads (×10^6^) | Total data (Gb) | Sequence coverage (fold) |
| --- | --- | --- | --- | --- | --- |
| 400 | 400 | 150 | 912.06 | 136.81 | 117 |

**Table S2** Statistics of normal and Ultra-long Nanopore reads

| Index | Libraries | | |
| --- | --- | --- | --- |
|  | Normal | Ultra-long1 | Ultra-long2 |
| Total Reads Base (Gb) | 95.41 | 10.62 | 19.47 |
| Clean Reads Base (Gb) | 82.56 | 9.68 | 18.26 |
| Clean Reads Number (Mb) | 3.51 | 0.72 | 0.38 |
| Clean Reads Mean Length (Kb) | 23.54 | 13.58 | 47.31 |
| Clean Reads N50 Length (Kb) | 32.86 | 44.36 | 104.55 |
| Clean Reads Max Length (Kb) | 239.69 | 1147.24 | 628.81 |

**Table S3** Statistics of contig length assembled with Nanopore reads

| Index | Length (Mb) | Number |
| --- | --- | --- |
| N10 | 159.97 | 1 |
| N20 | 81.44 | 2 |
| N30 | 72.35 | 4 |
| N40 | 46.68 | 6 |
| N50 | 32.81 | 9 |
| N60 | 26.31 | 13 |
| N70 | 20.81 | 18 |
| N80 | 13.46 | 25 |
| N90 | 6.19 | 38 |
| Min. | 0.60 | # |
| Max. | 159.97 | # |
| Ave. | 7.81 | # |
| Total | 1178.98 | 151 |

#: Not available.

**Table S4** Statistics of contig length assembled with Nanopore reads after polishing

| Index | Length (Mb) | Number |
| --- | --- | --- |
| N10 | 160.37 | 1 |
| N20 | 81.57 | 2 |
| N30 | 72.53 | 4 |
| N40 | 46.81 | 6 |
| N50 | 32.90 | 9 |
| N60 | 26.38 | 13 |
| N70 | 20.87 | 18 |
| N80 | 13.50 | 25 |
| N90 | 6.23 | 38 |
| Min. | 0.60 | # |
| Max. | 160.37 | # |
| Ave. | 7.83 | # |
| Total | 1182.52 | 151 |

#: Not available.

**Table S5** Statistics of Bionano BNX reads

| Index | F1.bnx | F2.bnx |
| --- | --- | --- |
| Maps | 582138 | 390574 |
| Sites | 23658873 | 23473604 |
| Length (Kb) | 146089462 | 113370606 |
| Average length (Kb) | 250.953 | 290.267 |
| Label Density (each 100 Kb) | 16.195 | 20.705 |
| N50 (Mb) | 0.2558 | 0.3049 |

**Table S6** Statistics of scaffolds generated from Bionano and Nanopore reads

| Index^1^ | Scaffold |
| --- | --- |
| Number of scaffolds | 69 |
| Min length | 0.168 |
| Median length | 4.023 |
| Mean length | 16.834 |
| N50 length | 72.533 |
| Max length | 160.370 |
| Total length | 1161.524 |

^1^unit for length is Mb.

**Table S7** Statistics of Hi-C reads

| Total Reads  Base (Gb) | Total Reads Number (Mb) | Clean Reads  Base (Gb) | Clean Reads Number (Mb) | Clean Reads  Rate (%) |
| --- | --- | --- | --- | --- |
| 307.47 | 2049.83 | 274.19 | 1827.96 | 89.18 |

**Table S8** Chromosome length of chicken (GRCg6a) and duck (SKLA1.0) genome

| Chicken | | | Duck | |
| --- | --- | --- | --- | --- |
| Chromosome | Accession number of contig | Length (Mb) | Chromosome | Length (Mb) |
| chr1 | NC_006088.5 | 197.61 | chr01 | 207.84 |
| chr2 | NC_006089.5 | 149.68 | chr02 | 160.52 |
| chr3 | NC_006090.5 | 110.84 | chr03 | 118.87 |
| chr4 | NC_006091.5 | 91.32 | chr04 | 76.57 |
| # | # | # | chr10 | 22.33 |
| chr5 | NC_006092.5 | 59.81 | chr05 | 66.40 |
| chr6 | NC_006093.5 | 36.37 | chr06 | 38.14 |
| chr7 | NC_006094.5 | 36.74 | chr07 | 39.93 |
| chr8 | NC_006095.5 | 30.22 | chr08 | 32.71 |
| chr9 | NC_006096.5 | 24.15 | chr09 | 26.70 |
| chr10 | NC_006097.5 | 21.12 | chr11 | 22.15 |
| chr11 | NC_006098.5 | 20.2 | chr12 | 21.81 |
| chr12 | NC_006099.5 | 20.39 | chr13 | 22.48 |
| chr13 | NC_006100.5 | 19.17 | chr14 | 20.47 |
| chr14 | NC_006101.5 | 16.22 | chr15 | 18.17 |
| chr15 | NC_006102.5 | 13.06 | chr16 | 15.60 |
| chr16 | NC_006103.5 | 2.84 | chr17 | 4.13 |
| chr17 | NC_006104.5 | 10.76 | chr18 | 12.03 |
| chr18 | NC_006105.5 | 11.37 | chr19 | 12.62 |
| chr19 | NC_006106.5 | 10.32 | chr20 | 11.97 |
| chr20 | NC_006107.5 | 13.9 | chr21 | 16.13 |
| chr21 | NC_006108.5 | 6.84 | chr22 | 8.53 |
| chr22 | NC_006109.5 | 5.46 | chr23 | 5.90 |
| chr23 | NC_006110.5 | 6.15 | chr24 | 8.06 |
| chr24 | NC_006111.5 | 6.49 | chr25 | 7.89 |
| chr25 | NC_006112.4 | 3.98 | chr26 | 3.24 |
| chr26 | NC_006113.5 | 6.06 | chr27 | 6.83 |
| chr27 | NC_006114.5 | 8.08 | chr28 | 7.25 |
| chr28 | NC_006115.5 | 5.12 | chr29 | 5.93 |
| # | # | # | chr30 | 5.46 |
| chr30 | NC_028739.2 | 1.82 | chr31 | 1.95 |
| chr31 | NC_028740.2 | 6.15 | # | # |
| chr32 | NC_006119.4 | 0.73 | # | # |
| chr33 | NC_008465.4 | 7.82 | # | # |
| # | # | # | chr32 | 5.24 |
| # | # | # | chr33 | 2.20 |
| # | # | # | chr34 | 4.82 |
| # | # | # | chr35 | 3.26 |
| # | # | # | chr36 | 3.57 |
| # | # | # | chr37 | 2.45 |
| # | # | # | chr38 | 1.91 |
| # | # | # | chr39 | 1.79 |
| chrW | NC_006126.5 | 6.81 | # | # |
| chrZ | NC_006127.5 | 82.53 | chrZ | 85.48 |

#: means not available.

**Table S9** RNA-seq data for assessing genome quality, gene annotation

| Sample number | Bases (Gb) | label / tissue / treatment / sample time |  |
| --- | --- | --- | --- |
| SRR5131562 | 5.12 | C1 / Breast muscle / # / 3 weeks | Wang L, Zhang H  RNA-seq of breast in 3-week and 6-week age native Pekin and Cherry Valley Pekin ducks  NCBI Bioproject  <https://www.ncbi.nlm.nih.gov/bioproject/PRJNA358835>  (2016) |
| SRR5131563 | 5.04 | C2 / Breast muscle / # / 3 weeks |  |
| SRR5131564 | 5.53 | C3 / Breast muscle / # / 6 weeks |  |
| SRR5131565 | 4.61 | C4 / Breast muscle / # / 6 weeks |  |
| SRR6812634 | 7.98 | # / Breast muscle / # / 42d | Yanhan Liu, Yaxiong Jia, Cun Liu, Limin Ding, and Zhaofei Xia  RNA-seq transcriptome of breast muscle in Anas platyrhynchos in response to Clostridium butyricum  NCBI Bioproject  <https://www.ncbi.nlm.nih.gov/bioproject/PRJNA437203>  (2018) |
| SRR6812635 | 9.60 | # / Breast muscle / # / 42d |  |
| SRR6812636 | 9.29 | # / Breast muscle / # / 42d |  |
| SRR6812637 | 10.08 | # / Breast muscle / # / 42d |  |
| SRR6812638 | 8.45 | # / Breast muscle / # /42d |  |
| SRR6812639 | 8.56 | # / Breast muscle / # /42d |  |
| SRR7811378 | 8.31 | C1 / Ileum / # / 72d | Xingchen Bu  RNA-Seq of different parts of small intestine from anas platyrhynchos under heat stress  NCBI Bioproject  <https://www.ncbi.nlm.nih.gov/bioproject/PRJNA489980>  (2019) |
| SRR7811379 | 7.15 | C2 / Ileum / # / 73d |  |
| SRR7811381 | 8.77 | C3 / Ileum / # / 74d |  |
| SRR7811367 | 7.72 | C1 / Duodenum / # / 62 d |  |
| SRR7811372 | 9.08 | C2 / Duodenum / # / 60 d |  |
| SRR7811373 | 9.26 | C3 / Duodenum / # / 61 d |  |
| SRR1610922 | 8.20 | C1 / Jejunum / # / 6 weeks | Feng Zhu, Jian-Ming Yuan, Zhen-He Zhang, Jin-Ping Hao, Yu-Ze Yang, Shen-Qiang Hu, Fang-Xi Yang, Lu-Jiang Qu, Zhuo-Cheng Hou  Anas platyrhynchos Transcriptome or Gene expression  NCBI Bioproject  <https://www.ncbi.nlm.nih.gov/bioproject/PRJNA263681>  (2014) |
| SRR1610923 | 8.07 | C2 / Jejunum / # / 6 weeks |  |
| SRR1610924 | 8.00 | C3 / Jejunum / # / 6 weeks |  |
| SRR1610925 | 6.92 | C4 / Jejunum / # / 6 weeks |  |
| **SRR1777194** | 11.24 | # / Brain / PBS /# | Yinhua Huang, Yingrui Li, David W Burt, Hualan Chen, Yong Zhang, Wubin Qian, Heebal Kim, Shangquan Gan, Yiqiang Zhao, Jianwen Li, Kang Yi, Huapeng Feng, Pengyang Zhu, Bo Li, Qiuyue Liu, Suan Fairley, Katharine E Magor, Zhenlin Du, Xiaoxiang Hu, Laurie Goodman, Hakim Tafer, Alain Vignal, Taeheon Lee, Kyu-Won Kim, Zheya Sheng, Yang An, Steve Searle, Javier Herrero, Martien A M Groenen, Richard P M A Crooijmans, Thomas Faraut, Qingle Cai, Robert G Webster, Jerry R Aldridge, Wesley C Warren, Sebastian Bartschat, Stephanie Kehr, Manja Marz, Peter F Stadler, Jacqueline Smith, Robert H S Kraus, Yaofeng Zhao, Liming Ren, Jing Fei, Mireille Morisson, Pete Kaiser, Darren K Griffin, Man Rao, Frederique Pitel, Jun Wang, Ning Li  Anas platyrhynchos gene expression response to flu infection  NCBI Bioproject  <https://www.ncbi.nlm.nih.gov/bioproject/PRJNA273367>  (2015) |
| **SRR3097704** | 13.52 | # / Brain / (H5N1 DK/49) / 1dpi |  |
| **SRR3097707** | 10.87 | # / Brain / (H5N1 DK/49) / 2dpi |  |
| **SRR3097710** | 10.21 | # / Brain / (H5N1 DK/49) / 3dpi |  |
| **SRR3097695** | 10.56 | # / Brain / (H5N1 GS/65) / 1dpi |  |
| **SRR3097698** | 8.74 | # / Brain / (H5N1 GS/65) / 2dpi |  |
| **SRR3097701** | 11.67 | # / Brain / (H5N1 GS/65) / 3dpi |  |
| **SRR1777192** | 12.47 | C7 / Lung / PBS / # |  |
| **SRR3097702** | 9.85 | T7 / Lung / (H5N1 DK/49) / 1dpi |  |
| **SRR3097705** | 11.01 | T8 / Lung / (H5N1 DK/49) / 2dpi |  |
| **SRR3097708** | 11.35 | T9 / Lung / (H5N1 DK/49) / 3dpi |  |
| **SRR1777195** | 11.75 | T10 / Lung / (H5N1 GS/65) / 1dpi |  |
| **SRR3097696** | 10.99 | T11 / Lung / (H5N1 GS/65) / 2dpi |  |
| **SRR3097699** | 9.78 | T12 / Lung / (H5N1 GS/65) / 3dpi |  |
| **SRR1777193** | 10.34 | C / Spleen / PBS / # |  |
| **SRR3097703** | 11.58 | T1 / Spleen / (H5N1 DK/49) / 1dpi |  |
| **SRR3097706** | 11.29 | T2 / Spleen / (H5N1 DK/49) / 2dpi |  |
| **SRR3097709** | 11.21 | T3 / Spleen / (H5N1 DK/49) / 3dpi |  |
| **SRR3097715** | 12.05 | T4 / Spleen / (H5N1 GS/65) / 1dpi |  |
| **SRR3097697** | 11.09 | T5 / Spleen / (H5N1 GS/65) / 2dpi |  |
| **SRR3097700** | 11.73 | T6 / Spleen / (H5N1 GS/65) / 3dpi |  |
| **SRR18913556** | 7.01 | C1 / Lung / PBS / 12hdpi | Jiaxiang Hu, Linfei Song, Mengfei Ning, Xinyu Niu, Mengying Han, Chuze Gao, Xingwei Feng, Han Cai, Te Li, Fangtao Li, Huifang Li, Daoqing Gong, Weitao Song, Long Liu, Juan Pu, Jinhua Liu, Jacqueline Smith, Honglei Sun, and Yinhua Huang  Anas platyrhynchos isolate:C18 \| breed:Pekin duck Genome sequencing and assembly  NCBI Bioproject  https://www.ncbi.nlm.nih.gov/bioproject/PRJNA792297  (2023) |
| **SRR18913555** | 8.34 | C2 / Lung / PBS / 12hdpi |  |
| **SRR18913554** | 7.61 | C3 / Lung / PBS / 12hdpi |  |
| **SRR18913561** | 6.65 | T1 / Lung / (H5N1 SY/08) / 12hdpi |  |
| **SRR18913560** | 7.24 | T2 / Lung / (H5N1 SY/08) / 12hdpi |  |
| **SRR18913557** | 7.50 | T3 / Lung / (H5N1 SY/08) / 12hdpi |  |
| **SRR18913550** | 9.06 | C4 / Lung / PBS / 24hdpi |  |
| **SRR18913559** | 8.77 | C5/ Lung / PBS / 24hdpi |  |
| **SRR18913558** | 8.73 | C6 / Lung / PBS / 24hdpi |  |
| **SRR18913551** | 7.99 | T4 / Lung / (H5N1 SY/08) / 24hdpi |  |
| **SRR18913553** | 7.83 | T5 / Lung / (H5N1 SY/08) / 24hdpi |  |
| **SRR18913552** | 9.16 | T6 / Lung / (H5N1 SY/08) / 24hdpi |  |
| SRR20707342 | 6.18 | C1 / Liver / # / 14 week | Yiming Wang, Linfei Song, Mengfei Ning, Jiaxiang Hu, Han Cai, Weitao Song, Daoqing Gong, Long Liu, Jacqueline Smith, Huifang Li, and Yinhua Huang  RNA-seq for duck liver tissue under fatty live formation and then recovery conditions.  NCBI Bioproject  https://www.ncbi.nlm.nih.gov/bioproject/PRJNA863477(2023) |
| SRR20707341 | 6.21 | C2 / Liver / # / 14 week |  |
| SRR20707330 | 6.21 | C3 / Liver / # / 14 week |  |
| SRR20707319 | 6.22 | C4 / Liver / # / 14 week |  |
| SRR20707318 | 6.2 | C5 / Liver / # / 14 week |  |
| SRR20707317 | 6.16 | # / Liver / overfeeding / 14 week |  |
| SRR20707316 | 6.18 | # / Liver / overfeeding / 14 week |  |
| SRR20707315 | 6.17 | # / Liver / overfeeding / 14 week |  |
| SRR20707314 | 6.02 | # / Liver / overfeeding / 14 week |  |
| SRR20707313 | 6.21 | # / Liver / overfeeding / 14 week |  |
| unpublished data | 6.22 | # / Liver / # / 15 week |  |
| unpublished data | 6.21 | # / Liver / # / 15 week |  |
| unpublished data | 6.23 | # / Liver / # / 15 week |  |
| unpublished data | 6.19 | # / Liver / # / 15 week |  |
| unpublished data | 6.23 | # / Liver / # / 15 week |  |
| unpublished data | 6.24 | # / Liver / overfeeding / 15 week |  |
| unpublished data | 6.24 | # / Liver / overfeeding / 15 week |  |
| unpublished data | 6.22 | # / Liver / overfeeding / 15 week |  |
| unpublished data | 6.19 | # / Liver / overfeeding / 15 week |  |
| unpublished data | 6.17 | # / Liver / overfeeding / 15 week |  |
| unpublished data | 6.24 | # / Liver / # / 16 week |  |
| unpublished data | 6.24 | # / Liver / # / 16 week |  |
| unpublished data | 6.22 | # / Liver / # / 16 week |  |
| unpublished data | 6.19 | # / Liver / # / 16 week |  |
| unpublished data | 6.17 | # / Liver / # / 16 week |  |
| unpublished data | 6.23 | # / Liver / overfeeding / 16 week |  |
| unpublished data | 6.21 | # / Liver / overfeeding / 16 week |  |
| unpublished data | 6.2 | # / Liver / overfeeding / 16 week |  |
| unpublished data | 6.17 | # / Liver / overfeeding / 16 week |  |
| unpublished data | 6.21 | # / Liver / overfeeding / 16 week |  |

#: means not available and no treatment. Datasets in bold text were from our lab. Dpi: days post infection.

**Table S10** Chromosome information of the duck SKLA1.0 genome

| Duck name | Size (Mb) | GC% | Number of coding gene | Protein |
| --- | --- | --- | --- | --- |
| chr01 | 207.84 | 40.15 | 2292 | 9593 |
| chr02 | 160.52 | 39.86 | 1460 | 6101 |
| chr03 | 118.87 | 40.08 | 1262 | 5484 |
| chr04 | 76.57 | 39.75 | 816 | 3790 |
| chr05 | 66.40 | 41.38 | 997 | 4263 |
| chr06 | 38.14 | 41.65 | 536 | 2337 |
| chr07 | 39.93 | 41.22 | 511 | 2115 |
| chr08 | 32.71 | 42.13 | 524 | 2359 |
| chr09 | 26.70 | 42.89 | 472 | 1819 |
| chr10 | 22.33 | 43.16 | 367 | 1385 |
| chr11 | 22.15 | 43.26 | 422 | 1605 |
| chr12 | 21.81 | 42.27 | 386 | 1387 |
| chr13 | 22.48 | 43.38 | 368 | 1469 |
| chr14 | 20.47 | 44.82 | 384 | 1371 |
| chr15 | 18.17 | 44.87 | 424 | 1594 |
| chr16 | 15.60 | 45.57 | 370 | 1484 |
| chr17 | 4.13 | 55.56 | 252 | 323 |
| chr18 | 12.03 | 48.15 | 309 | 1413 |
| chr19 | 12.62 | 47.02 | 317 | 1246 |
| chr20 | 11.97 | 46.93 | 329 | 1253 |
| chr21 | 16.13 | 45.66 | 378 | 1369 |
| chr22 | 8.53 | 47.69 | 265 | 892 |
| chr23 | 5.90 | 48.95 | 205 | 613 |
| chr24 | 8.06 | 50.71 | 253 | 867 |
| chr25 | 7.89 | 49.15 | 186 | 671 |
| chr26 | 3.24 | 57.57 | 330 | 729 |
| chr27 | 6.83 | 51.84 | 288 | 1001 |
| chr28 | 7.25 | 51.49 | 353 | 832 |
| chr29 | 5.93 | 52.67 | 315 | 952 |
| chr30 | 5.46 | 57.09 | 158 | 178 |
| chr31 | 1.95 | 59.47 | 89 | 213 |
| chr32 | 5.24 | 51.09 | 138 | 158 |
| chr33 | 2.20 | 57.81 | 101 | 144 |
| chr34 | 4.82 | 52.02 | 208 | 212 |
| chr35 | 3.26 | 57.72 | 222 | 512 |
| chr36 | 3.57 | 49.26 | 161 | 164 |
| chr37 | 2.45 | 55.38 | 110 | 117 |
| chr38 | 1.91 | 59.78 | 61 | 75 |
| chr39 | 1.79 | 50.38 | 59 | 97 |
| chrZ | 85.48 | 40.00 | 853 | 3553 |
| Un | 20.34 | 47.71 | 365 | 477 |

**Table S11** Distribution of transcript annotated in the BGI 1.0, SKLA1.0, ZJU1.0, GRCg6 and bTaeGut1.4.pri genome

| Number of transcripts per gene | SKLA1.0 | ZJU1.0 | BGI 1.0 | GRCg6 | bTaeGut  1.4.pri |
| --- | --- | --- | --- | --- | --- |
| 1 | 48.66% | 52.94% | 68.09% | 48.30% | 52.15% |
| 2 | 11.57% | 16.72% | 12.11% | 18.75% | 19.45% |
| 3 | 8.44% | 9.30% | 6.60% | 10.03% | 9.80% |
| 4 | 6.28% | 5.94% | 3.96% | 6.54% | 6.06% |
| 5 | 4.73% | 3.78% | 2.55% | 4.45% | 3.70% |
| 6 | 3.98% | 2.88% | 1.82% | 2.91% | 2.59% |
| 7 | 3.13% | 2.15% | 1.34% | 1.90% | 1.57% |
| 8 | 2.55% | 1.42% | 0.74% | 1.59% | 0.98% |
| 9 | 1.94% | 1.00% | 0.70% | 1.04% | 0.79% |
| >=10 | 8.72% | 3.88% | 2.09% | 4.49% | 2.91% |

**Table S12** Expanded/contracted immune-related gene families in duck

| Panther ID | Family name | Number of genes | | | P-value |
| --- | --- | --- | --- | --- | --- |
|  |  | chicken | duck | chicken and duck  common ancestor |  |
| PTHR10271 | INTERFERON-INDUCED PROTEIN WITH TETRATRICOPEPTIDE REPEATS | 1 | 2 | 1 | 0.027 |
| PTHR10656 | CELL FATE DETERMINING PROTEIN MAB21-RELATED | 15 | 26 | 15 | 0 |
| PTHR10728 | CYTOSOLIC PHOSPHOLIPASE A2 | 10 | 12 | 10 | 0.007 |
| PTHR10751 | GUANYLATE-BINDING PROTEIN 2 | 6 | 8 | 6 | 0 |
| PTHR11258 | 2-5-OLIGOADENYLATE SYNTHASE 1 | 2 | 1 | 2 | 0.004 |
| PTHR11334 | MAS-RELATED G-PROTEIN COUPLED RECEPTOR MEMBER X2 | 5 | 6 | 5 | 0.002 |
| PTHR11412 | COMPLEMENT C4-A-RELATED | 14 | 16 | 14 | 0 |
| PTHR11461 | PLASMINOGEN ACTIVATOR INHIBITOR | 27 | 28 | 27 | 0.032 |
| PTHR11559 | ACETYLCHOLINESTERASE | 4 | 2 | 3 | 0.002 |
| PTHR11571 | GLUTATHIONE S-TRANSFERASE P | 9 | 7 | 8 | 0 |
| PTHR11639 | PROTEIN S100-A1-RELATED | 13 | 15 | 13 | 0 |
| PTHR11710 | 40S RIBOSOMAL PROTEIN S19 | 1 | 0 | 1 | 0.034 |
| PTHR11908 | XANTHINE DEHYDROGENASE/OXIDASE | 3 | 2 | 3 | 0.01 |
| PTHR11937 | ACTIN-RELATED PROTEIN 3 | 23 | 21 | 22 | 0 |
| PTHR12080 | SLAM FAMILY MEMBER 7 | 9 | 13 | 10 | 0 |
| PTHR19143 | FICOLIN-1 | 25 | 23 | 25 | 0.004 |
| PTHR19441 | WAP FOUR-DISULFIDE CORE DOMAIN PROTEIN 12 | 2 | 1 | 2 | 0 |
| PTHR19944 | MHC CLASS II-RELATED | 6 | 26 | 15 | 0 |
| PTHR19971 | SIGNAL-REGULATORY PROTEIN BETA-1-RELATED | 3 | 7 | 3 | 0 |
| PTHR22802 | C-TYPE LECTIN DOMAIN FAMILY MEMBER | 4 | 11 | 8 | 0 |
| PTHR22906 | PROPERDIN | 1 | 0 | 1 | 0.035 |
| PTHR23193 | NUCLEAR PORE COMPLEX PROTEIN NUP153 | 3 | 9 | 4 | 0 |
| PTHR23266 | IMMUNOGLOBULIN HEAVY CONSTANT EPSILON | 4 | 5 | 4 | 0 |
| PTHR23267 | IMMUNOGLOBULIN KAPPA VARIABLE 1-8-RELATED | 2 | 3 | 2 | 0 |
| PTHR24006 | INACTIVE UBIQUITIN CARBOXYL-TERMINAL HYDROLASE 17-LIKE PROTEIN 4-RELATED | 23 | 34 | 25 | 0 |
| PTHR24064 | SOLUTE CARRIER FAMILY 22 MEMBER 3 | 15 | 17 | 16 | 0.011 |
| PTHR24100 | BUTYROPHILIN | 3 | 47 | 7 | 0 |
| PTHR24118 | B-CELL LYMPHOMA 3 PROTEIN | 5 | 4 | 5 | 0.002 |
| PTHR24247 | HISTAMINE H4 RECEPTOR | 23 | 27 | 24 | 0.003 |
| PTHR24253 | COAGULATION FACTOR XI | 16 | 14 | 16 | 0 |
| PTHR24271 | KALLIKREIN-1 | 8 | 9 | 8 | 0 |
| PTHR24399 | ZINC FINGER AND BTB DOMAIN-CONTAINING PROTEIN 1 | 17 | 18 | 19 | 0 |
| PTHR45615 | MYOSIN-9 | 17 | 19 | 18 | 0.005 |
| PTHR45710 | C-TYPE LECTIN DOMAIN-CONTAINING PROTEIN 180 | 24 | 22 | 18 | 0 |
| PTHR46393 | COMPLEMENT FACTOR B | 1 | 0 | 1 | 0.033 |
| PTHR46746 | KILLER CELL LECTIN-LIKE RECEPTOR | 2 | 17 | 2 | 0 |
| GROUP_01 | DEATH INDUCER-OBLITERATOR 1 | 1 | 6 | 1 | 0 |
| GROUP_02 | MHC CLASS I | 2 | 5 | 3 | 0 |

**Table S13** Primer sequences used for PCR amplification of duck NKC region in the MHC

| Fragment | Forward(F) or Reserved(R) | sequence |
| --- | --- | --- |
| Chr17:529571bp-527616bp | F | CCACTTCACCAAGATGCCAGTTCCT |
|  | R | GCTCGGTGCCTCTATCCAAC |
| Chr17:534321bp-537206bp | F | GGTTTTTTTTGCGTGAGTCAGTGT |
|  | R | ATCATCTGTTGTGTTGCACTTCAAG |
| Chr17:693844bp-695032bp | F | TACGCAGCCAATGTGGTTTTAACAC |
|  | R | AACCCCTATTAAACCTTGAACACTG |
| Chr17:805394bp-807595bp | F | AATTAGCAAGGGAACTTGAAACTTG |
|  | R | AACGGCCAATGTGCCTACATC |
| Chr17:810867bp-813348bp | F | AGTTGTAGTCTTCGGCCCTGTAAT |
|  | R | TTAATCTTAATGGTGGTGTGTGTTG |

**Table 14** Information on conserved blocks in amphibian MHC

| Species | Genome version | Chromosome | Syntenic region | Location (bp) |
| --- | --- | --- | --- | --- |
| Bombina bombina  (fire-bellied toads) | aBomBom1.pri | 7 | C-D block | 554287467 - 558275741 |
|  |  |  | BTN family | 550521094 - 551017097 |
|  |  |  | D-P block | 580316374 - 581817563 |
|  |  |  | Class III | 571261905 - 579748639 |
|  |  |  | Class I | 560339069 - 565139263 |
|  |  |  | Class II | 559199676 - 560085498 |
| Bufo gargarizans  (true toad) | ASM1485885v1 | 9 | C-D block | 21774760 - 22092684 |
|  |  |  | BTN family | 550521094 - 551017097 |
|  |  |  | D-P block | 12443174 - 12681255 |
|  |  |  | Class III | 571261905 - 579748639 |
|  |  |  | Class I | 560339069 - 565139263 |
|  |  |  | Class II | 559199676 - 560085498 |
| Bufo bufo  (common toad) | aBufBuf1.1 | 8 | C-D block | 209322216 - 209649989 |
|  |  |  | BTN family | 210410727 - 210506201 |
|  |  |  | D-P block | 199861958 - 200100828 |
|  |  |  | Class III | 205080174 - 206960466 |
|  |  |  | Class I | 207443581 - 208407353 |
|  |  |  | Class II | 208421155 - 208914446 |
|  |  |  | NKC region | 9125924 - 9131715 |
| Hyla sarda  (tyrrhenian tree frog) | aHylSar1.hap1 | 9 | C-D block | 171514002 - 171914914 |
|  |  |  | BTN family | 172936173 - 173058940 |
|  |  |  | D-P block | 163600942 - 163994366 |
|  |  |  | Class III | 163219011 - 169640159 |
|  |  |  | Class I | 170164847 - 170718370 |
|  |  |  | Class II | 170737692 - 171110036 |
|  |  |  | NKC region | 8572673 - 8582969 |
| Microcaecilia  unicolor  (tiny cayenne  caecilian) | aMicUni1.1 | 3 | C-D block | 205219733 - 205792126 |
|  |  |  | BTN family | 216765382 - 230458253 |
|  |  |  | D-P block | 210879450 - 216646925 |
|  |  |  | Class III | 208241009 - 210166222 |
|  |  |  | Class I | 207215077 - 207614732 |
|  |  |  | Class II | 205809401 - 206403046 |
|  |  |  | NKC region | 202228993 - 202272003 |
| Rana temporaria  (common frog) | aRanTem1.1 | 9 | C-D block | 168369728 - 168992051 |
|  |  |  | BTN family | 170530437 - 170660420 |
|  |  |  | D-P block | 51956907 - 52618037 |
|  |  |  | Class III | 29988466 - 129284356 |
|  |  |  | Class I | 163887922 - 167048987 |
|  |  |  | Class II | 167061937 - 167671202 |
|  |  |  | NKC region | 136869412 - 136909143 |
| Xenopus tropicalis  (tropical clawed  frog) | UCB_Xtro_10.0 | 8 | C-D block | 72026094 - 72205156 |
|  |  |  | BTN family | 134809201 - 135945754 |
|  |  |  | D-P block | 75279984 - 75499159 |
|  |  |  | Class III | 73620813 - 74143733 |
|  |  |  | Class I | 133793794 - 134423265 |
|  |  |  | Class II | 72939510 - 73245385 |
|  |  |  | NKC region | 80578849 - 80588343 |

**Table 15** Information on conserved blocks in reptilian MHC

| Species | Genome version | Chromosome | Syntenic region | Location (bp) |
| --- | --- | --- | --- | --- |
| Eublepharis macularius  (leopard geckos) | MPM_Emac_v1.0 | 4 | C-D block | 184517247 - 188207737 |
|  |  |  | D-P block | 156272863 - 158929761 |
|  |  |  | Class III | 163440248 - 165761850 |
|  |  |  | Class I | 176566283 - 185282037 |
|  |  |  | Class II | 186310527 - 188178441 |
|  |  |  | NKC region | 157577830 - 159380030 |
| Euleptes europaea  (leaf-toed gecko) | rEulEur1.hap1 | 1 | C-D block | 146311 - 555327 |
|  |  |  | BTN family | 27556660 - 27564139 |
|  |  |  | D-P block | 23207402 - 27219987 |
|  |  |  | Class III | 17040327 - 19828786 |
|  |  |  | Class I | 1341574 - 4177213 |
|  |  |  | Class II | 248140 - 1106271 |
|  |  |  | NKC region | 23974681 - 24741698 |
| Gopherus  flavomarginatus  (bolson tortoises) | rGopFla2.mat.asm | 12 | C-D block | 162819 - 350623 |
|  |  |  | BTN family | 6480559 - 8903588 |
|  |  |  | D-P block | 5622290 - 6061908 |
|  |  |  | Class III | 1620309 - 1653906 |
|  |  |  | Class I | 923345 - 1324334 |
|  |  |  | Class II | 417633 - 921323 |
|  |  |  | NKC region | 3062736 - 5486298 |
| Hemicordylus  capensis  (cape cliff lizard) | rHemCap1.1.pri | 2 | C-D block | 225182077 - 225810102 |
|  |  |  | BTN family | 262702547 - 262719295 |
|  |  |  | D-P block | 258714752 - 262350899 |
|  |  |  | Class III | 250345761 - 253553411 |
|  |  |  | Class I | 228928720 - 242659581 |
|  |  |  | Class II | 225330170 - 226597610 |
|  |  |  | NKC region | 258344026 - 260548278 |
| Lacerta agilis  (sand lizards) | rLacAgi1.pri | 2 | C-D block | 119010123 - 120616599 |
|  |  |  | BTN family | 107341638 - 107567443 |
|  |  |  | D-P block | 103919293 - 106111605 |
|  |  |  | Class III | 108189727 - 109485230 |
|  |  |  | Class I | 115000543 - 115899740 |
|  |  |  | Class II | 116434270 - 120162741 |
|  |  |  | NKC region | 105239801 - 105888754 |
| Malaclemys  terrapin pileata  (mississippi  diamondback terrapin) | rMalTer1.hap1 | 13 | C-D block | 46317918 - 46511659 |
|  |  |  | BTN family | 27219807 - 42260974 |
|  |  |  | D-P block | 42809542 - 44552791 |
|  |  |  | Class I | 45597674 - 45971452 |
|  |  |  | Class II | 45980866 - 46313743 |
|  |  |  | NKC region | 43373286 - 44700566 |
| Podarcis muralis  (common wall lizard) | PodMur_1.0 | 2 | C-D block | 125252195 - 126757978 |
|  |  |  | BTN family | 114303386 - 117756758 |
|  |  |  | D-P block | 503342 - 1402582 |
|  |  |  | Class III | 112081735 - 113245389 |
|  |  |  | Class I | 117849035 - 124368965 |
|  |  |  | Class II | 125544244 - 127890757 |
|  |  |  | NKC region | 108709965 - 109386969 |
| Podarcis raffonei  (aeolian wall lizard) | rPodRaf1.pri | 2 | C-D block | 125369432 - 127243830 |
|  |  |  | BTN family | 113004918 - 117583859 |
|  |  |  | D-P block | 108998272 - 111688513 |
|  |  |  | Class I | 120886641 - 121733257 |
|  |  |  | Class II | 124644457 - 126126122 |
|  |  |  | NKC region | 110813520 - 111463776 |

**Table S16** Information on conserved blocks in water fowl, land fowl and mammals MHC map

| Taxonomy | Species | Genome version | Chromosome  or Scaffold | Syntenic region | Location (bp) |
| --- | --- | --- | --- | --- | --- |
| Water fowls | Anas platyrhynchos  (duck) | SKLA1.0 | Chr17 | D-P block | 208275- 490032 |
|  |  |  |  | NKC region | 524444- 802384 |
|  |  |  |  | BTN family | 990420- 1215278 |
|  |  |  |  | Class III | 1304942- 1414666 |
|  |  |  |  | BTN region | 1347785- 1394161 |
|  |  |  |  | Class I | 1417920- 1446852 |
|  |  |  |  | BTN region | 1462921- 1675601 |
|  |  |  |  | Class II | 1698243- 1739459 |
|  |  |  |  | C-D block | 1744410- 1863114 |
|  | Cygnus olor  (mute swan) | bCygOlo1.pri.v2 | NW_024429053.1  (chr33 Un) | D-P block | 17877-92164 |
|  |  |  | Chr33 | NKC region | 499968-726245 |
|  |  |  |  | BTN family | 396928-482653 |
|  |  |  |  | Class III | 254537-344253 |
|  |  |  |  | BTN family | 280114-284918 |
|  |  |  |  | Class I | 230272-252041 |
|  |  |  |  | BTN family | 173327-446987 |
|  |  |  |  | Class II | 127448-139557 |
|  |  |  |  | C-D block | 102487-123420 |
| Land birds | Gallus gallus  (chicken) | GRCg7b | Chr16 | C-D block (partial) | 2346599-2384376 |
|  |  |  |  | Class III | 2230489-2282627 |
|  |  |  |  | Class I | 2213444-2229459 |
|  |  |  |  | Class II | 2185532-2194309 |
|  |  |  |  | NKC region | 2178712-2185541 |
|  |  |  |  | C-D block (partial) | 1986912-2009545 |
|  |  |  |  | NKC region | 1222428-1832691 |
|  |  |  | NW_024095939.1  (chr16 Un) | D-P block (partial) | 84514-189140 |
|  | Meleagris gallopavo  (turkey) | Turkey_5.1 | Chr18 | Class III | 13691-69861 |
|  |  |  |  | Class I | 70320-85674 |
|  |  |  |  | Class II | 114229-115023 |
|  |  |  |  | NKC region | 119228-126369 |
|  | Numida meleagris  (helmeted guineafowl) | NumMel1.0 | Chr15 | Class III | 4-39931 |
|  |  |  |  | Class I | 37482-45478 |
|  |  |  |  | Class II | 77812-91820 |
|  |  |  |  | NKC region | 91849- 104161 |
|  | Coturnix japonica  (Japanese quail) | Coturnix japonica 2.1 | Chr16 | Class III | 9633-76146 |
|  |  |  |  | Class I | 79801-131088 |
|  |  |  |  | Class II | 132794-177772 |
|  |  |  |  | NKC region | 171812-186884 |
| Mammals | Homo sapiens  (human) | GRCh38.p13 | Chr6 | BTN family | 26500303- 26453415 |
|  |  |  |  | D-P block | 30066864-30914106 |
|  |  |  |  | Class I | 29942532-31357179 |
|  |  |  |  | Class III | 31982057-32109338 |
|  |  |  |  | Class II | 32439887-33089696 |
|  |  |  |  | C-D block | 33162692-33322959 |
|  |  |  | Chr12 | NKC region | 8950044-9215657 |
|  | Pan troglodytes  (chimpanzee) | NHGRI_mPanTro3-v1.1-hic.freeze_pri | Chr6 | BTN family | 18639284-18773752 |
|  |  |  |  | D-P block | 29575714-30389594 |
|  |  |  |  | Class I | 29448067-30848748 |
|  |  |  |  | Class III | 30946245-31521178 |
|  |  |  |  | Class II | 31875028-32656904 |
|  |  |  |  | C-D block | 32677085-32837462 |
|  |  |  | Chr12 | NKC region | 9140902-9459838 |

**Table S17** Gene information in D-P and C-D blocks

| Gene | | Location in duck | Homology in human (gene location) | Description |
| --- | --- | --- | --- | --- |
| D-P block | DDR1 | 208275-236459 | NP_001189452.2 (30880958-30900156) | discoidin domain receptor  tyrosine kinase 1 |
|  | S100A16 | 282616-285787 | No | protein S100-A16-like |
|  | GTF2H4 | 291680-306528 | NP_001508.1 (30908207-30914106) | general transcription factor  IIH subunit 4 |
|  | DHX16 | 368126-396218 | NP_003578.2 (30653127-30673006) | DEAH-box helicase 16 |
|  | C6ORF136 | 396433-408435 | NP_659466.2 (30647133-30653207) | chromosome 6 open reading  frame 136 |
|  | ATAT1 | 404025-413805 | NP_001241881.1 (30626874-30646821) | alpha tubulin acetyltransferase 1 |
|  | MRPS18B | 414802-417124 | NP_054765.1 (30617320-30626393) | mitochondrial ribosomal  protein S18B |
|  | PPP1R10 | 424813-438001 | NP_001363124.1 (30600400-30618607) | protein phosphatase 1 regulatory  subunit 10 |
|  | ABCF1 | 440812-457644 | NP_001020262.1 (30571442-30591522) | ATP binding cassette subfamily  F member 1 |
|  | GNL1 | 457964-467205 | NP_005266.2 (30541381-30557199) | G protein nucleolar 1 |
|  | TRIM39 | 471703-478057 | NP_001356450.1 (30326469-30343729) | tripartite motif containing 39 |
|  | PPP1R11 | 485558-490032 | NP_068778.1 (30066864-30070333) | protein phosphatase 1 regulatory  inhibitor subunit 11 |
| C-D block | COL11A2 | 1744410-1766390 | XP_016855823.1 (33162692-33193009) | collagen type XI alpha  1 chain |
|  | RXRB | 1770238-1781343 | NP_002948.1 (33193588-33200991) | retinoid X receptor alpha |
|  | SLC39A7 | 1786147-1786825 | NP_001275706.1 (33200867-33204437) | solute carrier family 39 member 7 |
|  | HSD17B8 | 1791852-1795909 | NP_055049.1 (33204655-33206831) | hydroxysteroid 17-beta  dehydrogenase 8 |
|  | RING1 | 1796397-1805008 | XP_005245470.1 (33208500-33212716) | ring finger protein 2 |
|  | VPS52 | 1805616-1810491 | NP_072047.4 (33250272-33271965) | VPS52 subunit of GARP complex |
|  | RPS18 | 1810594-1815028 | NP_072045.1 (33272075-33276511) | ribosomal protein S18 |
|  | WDR46 | 1815123-1816906 | NP_001157739.1 (33278207-33289239) | WD repeat domain 46 |
|  | B3GALT4 | 1817932-1819746 | NP_003773.1 (33277123-33278825) | beta-1,3-galactosyltransferase 4 |
|  | PFDN6 | 1820719-1822705 | NP_001172110.1 (33289597-33290934) | prefoldin subunit 6 |
|  | RGL2 | 1829013-1834133 | XP_024302273.1 (33291654-33299365) | ral guanine nucleotide dissociation  stimulator like 2 |
|  | TAPBP | 1837706-1843225 | NP_003181.3 (33299694-33314254) | TAP binding protein |
|  | ZBTB22 | 1844613-1846175 | NP_001138810.1 (33314418-33317942) | zinc finger and BTB domain  containing 22 |
|  | DAXX | 1849795-1863114 | NP_001135441.1 (33318558-33322959) | death domain associated protein |

In duck, D-P block and C-D block are located in chromosome 17.

In human, D-P block and C-D block are located in chromosome 6.

**Table S18** RMSD and GMQE values of predicted structures of duck MHC genes to templates

| Gene ID | Gene Symbol | Template | RMSD | GMQE |
| --- | --- | --- | --- | --- |
| DUCK_11735 | AnplUAA | 5GJX:A | 0.000 | 0.89 |
| DUCK_11734 | AnplUBA |  | 0.000 | 0.89 |
| DUCK_11733 | AnplUCA |  | 0.000 | 0.88 |
| DUCK_11732 | AnplUDA |  | 0.000 | 0.88 |
| DUCK_11731 | AnplUEA |  | 0.000 | 0.89 |
| DUCK_11792 | TAPBP | 6ENY:C | 0.653 | 0.6 |
| DUCK_11771 | AnplMHCIIα | 6KVM:B | 0.620 | 0.83 |
| DUCK_11772 | AnplMHCIIβ1 |  | 0.689 | 0.84 |
| DUCK_11773 | AnplMHCIIβ2 |  | 1.094 | 0.84 |
| DUCK_11774 | AnplMHCIIβ3 |  | 0.995 | 0.83 |
| DUCK_11775 | AnplMHCIIβ4 |  | 0.708 | 0.84 |
| DUCK_11776 | AnplMHCIIβ5 |  | 0.581 | 0.84 |
| DUCK_11777 | AnplMHCIIβ6 |  | 0.865 | 0.85 |
| DUCK_11778 | AnplMHCIIβ7 |  | 0.776 | 0.85 |
| DUCK_11779 | AnplMHCIIβ8 |  | 0.671 | 0.85 |
| DUCK_11780 | AnplMHCIIβ9 |  | 0.668 | 0.84 |
| DUCK_11769 | AnplDMA | 4FQX:C | 1.681 | 0.81 |
| DUCK_11768 | AnplDMB1 | 4FQX:D | 0.931 | 0.75 |
| DUCK_11722 | AnplDMB2-1 |  | 0.768 | 0.73 |
| DUCK_11728 | AnplDMB2-2 |  | 0.730 | 0.61 |
| DUCK_11740 | AnplDMB2-3 |  | 1.438 | 0.56 |
| DUCK_11744 | AnplDMB2-4 |  | 0.511 | 0.69 |
| DUCK_11747 | AnplDMB2-5 |  | 0.493 | 0.41 |
| DUCK_11749 | AnplDMB2-6 |  | 2.142 | 0.45 |
| DUCK_11751 | AnplDMB2-7 |  | 0.565 | 0.68 |
| DUCK_11753 | AnplDMB2-8 |  | 0.458 | 0.69 |
| DUCK_11755 | AnplDMB2-9 |  | 1.286 | 0.52 |
| DUCK_11757 | AnplDMB2-10 |  | 0.521 | 0.78 |
| DUCK_11760 | AnplDMB2-11 |  | 0.452 | 0.57 |
| DUCK_11762 | AnplDMB2-12 |  | 0.868 | 0.50 |
| DUCK_11764 | AnplDMB2-13 |  | 0.652 | 0.54 |
| DUCK_11767 | AnplDMB2-14 |  | 0.699 | 0.78 |
| DUCK_18203 | NKRP1-1 | 5J2S:A | 0.715 | 0.68 |
| DUCK_18205 | NKRP1-2 |  | 0.513 | 0.69 |
| DUCK_18207 | NKRP1-3 |  | 0.372 | 0.69 |
| DUCK_18210 | NKRP1-6 |  | 0.941 | 0.69 |
| DUCK_18212 | NKRP1-7 |  | 0.676 | 0.66 |
| DUCK_18218 | NKRP1-10 |  | 1.025 | 0.66 |
| DUCK_18225 | NKRP1-13 |  | 0.891 | 0.69 |
| DUCK_18231 | NKRP1-15 |  | 1.001 | 0.69 |
| DUCK_18236 | NKRP1-16 |  | 1.118 | 0.69 |
| DUCK_18202 | CLEC2-2 | 4QKH:A | 0.449 | 0.84 |
| DUCK_18204 | CLEC2-3 |  | 1.019 | 0.81 |
| DUCK_18206 | CLEC2-4 |  | 1.492 | 0.78 |
| DUCK_18211 | CLEC2-5 |  | 0.493 | 0.81 |
| DUCK_18213 | CLEC2-6 |  | 1.348 | 0.81 |
| DUCK_18219 | CLEC2-9 |  | 0.561 | 0.81 |
| DUCK_18224 | CLEC2-12 |  | 0.329 | 0.79 |
| DUCK_18226 | CLEC2-13 |  | 0.517 | 0.75 |
| DUCK_18232 | CLEC2-17 |  | 0.463 | 0.76 |
| DUCK_18235 | CLEC2-20 |  | 0.730 | 0.77 |

AnplUAA, AnplUBA, AnplUCA, AnplUDA and AnplUEA were made point mutation and optimized using the Discovery Studio 2019. The other proteins were predicted with the I-TASSER (https://zhanggroup.org//I-TASSER/).

**Table S19** Promoter of the duck TAPBP gene

| Start | End | Score | Promoter Sequence |
| --- | --- | --- | --- |
| 39 | 89 | 0.88 | CTGGCCCCGCCCCCATTTAAGCCACGCCCCCATTTGAGCCACGCCCCTTT |
| 133 | 183 | 0.95 | CCCCCTCCCCTTTAAGCCCCGCCCACCCCCCAG  CAGGAGGCCGGAGGCGG |
| 242 | 292 | 1.00 | CTCAAAATACAAAAAAATGGGGACAGGGGGAG  GGGCACGGTCCCCACCCC |
| 1397 | 1447 | 0.83 | CCCCCCGGCGAACACCCCCTCCCCGCGTGGCGA  CCCGGCCACCGGCTCCT |

**Table S20** Species used in the maximum likelihood tree

| Abbreviation | Species name | common name |
| --- | --- | --- |
| Ama | Apteryx mantelli mantelli | brown kiwi |
| Apl | Anas platyrhynchos | duck |
| Aro | Apteryx rowi | Okarito brown kiwi |
| Cat | Cygnus atratus | black swan |
| Col | Cygnus olor | mute swan |
| Cti | Crotalus tigris | Tiger rattlesnake |
| Dno | Dromaius novaehollandiae | emu |
| Eca | Equus caballus | horse |
| Gga | Gallus gallus | chicken |
| Hsa | Homo sapiens | human |
| Lag | Lacerta agilis | Sand lizard |
| Mga | Meleagris gallopavo | turkey |
| Mmu | Mus musculus | mouse |
| Mpu | Mustela putorius furo | domestic ferret |
| Mun | Microcaecilia unicolor | caecilians |
| Nme | Numida meleagris | helmeted guineafowl |
| Npa | Nanorana parkeri | Altirana parkeri |
| Pgu | Pantherophis guttatus | Corn Snake |
| Pmu | Podarcis muralis | Common wall lizard |
| Ptr | Pan troglodytes | chimpanzee |
| Rae | Rousettus aegyptiacus | Egyptian rousette |
| Rbi | Rhinatrema bivittatum | two-lined caecilian |
| Rfe | Rhinolophus ferrumequinum | greater horseshoe bat |
| Rno | Rattus norvegicus | Norway rat |
| Sca | Struthio camelus australis | ostrich |
| Tel | Thamnophis elegans | Western terrestrial garter snake |
| Tgu | Tinamus guttatus | white-throated tinamou |
| Xla | Xenopus laevis | African clawed frog |
| Xtr | Xenopus tropicalis | tropical clawed frog |
| Zvi | Zootoca vivipara | common lizard |

**Table S21** S-X-Y motifs in the promoter of BTN genes

| Gene name | Motif S | Motif X | Motif Y |
| --- | --- | --- | --- |
| BTN-1 | GGGTTTT | CCTAGTAACTGC | ACCATTGG |
| BTN-4 | GGATTGT | CCTAGTAACTGC | ACCATTGG |
| BTN-5 | GGGTTTT | CCTAGTAACTGC | ACCATTGG |
| BTN-8 | GGGTTTT | CCTAGTAACAAG | ACCATTGG |
| BTN-9 | GGATTGT | CCTAGTAACTGC | ACTATTGG |
| BTN-12 | GGGTTTT | CCTAGTAACTGC | ACCATTGG |
| BTN-14 | GGATTGT | CCTAGTAACTGC | ACCATTGG |
| BTN-20 | GGGTTTT | CCTAGTAACTGC | ACTATTGG |
| BTN-21 | GGGTTTT | CCTAGTAACTGC | ACTATTGG |
| BTN-22 | GGGTTTT | CCTAGTAACTGC | ACTATTGG |
| BTN-26 | GGATTGT | CCTAGTAACTGC | ACCATTGG |
| BTN-37 | GGATTGT | CCTAGTAACTGC | ACTATTGG |
| BTN-38 | GGATTGT | CCTAGTAACAAG | ACTATTGG |
| BTN-40 | GGATTGT | CCTAGTAACAAG | ACTATTGG |
| BTN-41 | GGGTTTT | CCTAGTAACTGC | ACCATTGG |
| BTN-42 | GGGTTTT | CCTAGTAACAAG | ACTATTGG |

**Table S22** Primer sequences used for the quantitative PCR analysis

| gene name | Forward (F) or Reverse (R) | sequence |
| --- | --- | --- |
| Duck BCL6 | F | TGAGCGTGAATGGAGAAGAC |
|  | R | ACATCTCAGCATGTTGTGGG |
| Duck AICDA | F | AAGGGCCGTCGTGAAAC |
|  | R | GCTGAGATGTAGCGTAGGAA |
| Duck GAPDH | F | GCTGCCCAGAACATTATCC |
|  | R | CAGGTCAGGTCCACGACA |
| Chicken BCL6 | F | TGTCAAATCTAGCGAAGCTG |
|  | R | CCAGGCAAACTGTTGAATAA |
| Chicken AICDA | F | CCTACCCAAACTTGACCCTC |
|  | R | TGTCTTTTCCCTGTTCTCCA |
| Chicken GAPDH | F | CCAGAACATCATCCCAGCGTC |
|  | R | CGGCAGGTCAGGTCAACAAC |

Hpi: hours post infection.

**Table S23** Expression of *CD8A* and *CD8A1* genes in duck and chicken spleen tissue

| Species | Number of samples | CD8A | CD8A1 |
| --- | --- | --- | --- |
| duck | 1 | 34.92 | 0.72 |
| chicken | 3 | 69.55 | 186.23 |

Expression of Chicken CD8A1 is the total expression of 24 CD8A1 genes

**Table S24** Expression of *CD8A* and *CD8A1* genes in duck and chicken lung tissues at 24hpi

| Gene | | C4 | C5 | C6 | T4 | T5 | T6 | Log_2_(FC) | P value |
| --- | --- | --- | --- | --- | --- | --- | --- | --- | --- |
| Duck | CD8A | 4.51 | 4.37 | 9.19 | 2.92 | 2.68 | 4.12 | -0.83 | 0.02 |
|  | CD8A1 | 0.22 | 0.20 | 0.48 | 0.24 | 0.10 | 0.08 | -1.07 | 0.18 |
| Chicken | CD8A | 8.60 | 8.43 | 9.95 | 23.87 | 25.98 | 5.20 | 1.27 | 0.01 |
|  | CD8A1 | 4.30 | 5.56 | 7.13 | 5.58 | 9.13 | 3.88 | 0.13 | 0.82 |

Hpi: hours post infection.

Animals in C and T group were infected by PBS and SY/08 H5N1 virus respectively.

Expression of Chicken CD8A1 is the total expression of 24 CD8A1 genes
