# Supplementary material for: A new chromosome-scale duck genome shows a major histocompatibility complex with several expanded multigene families
Source: BMC Biol. 2024 Feb 5;22:31. doi: 10.1186/s12915-024-01817-0 (PMC10845735; doi:10.1186/s12915-024-01817-0)
Supplement: Supplementary file 2 — Additional file 2: Figure S1- Figure S18. Fig. S1 – [Photo of C18 duck]. Fig. S2 - [17-mer distribution curve]. Fig. S3 - [K-mer depth distribution curve]. Fig. S4 – [Gene expansion and contraction across a phylogenetic tree of eight species]. Fig. S5 – [Conserved synteny of MHC gene map in duck and chicken]. Fig. S6 – [Expressional profile of duck MHCIα genes in eight tissues]. Fig. S7 – [Multiple sequence alignment of MHCIα proteins]. Fig. S8 – [Opening size of the peptide-binding pocket of duck and chicken MHCIα proteins]. Fig. S9 – [Electrostatic potential of peptide-binding pocket of duck and chicken MHCIα proteins]. Fig. S10 – [Lipophilic potential of pocket B of duck and chicken MHCIα proteins]. Fig. S11 – [Electrostatic potential of the peptide-binding pocket of MHCIα proteins in another two ducks]. Fig. S12 – [Lipophilic potential of pocket B of MHCIα proteins in another two ducks]. Fig. S13 – [Maximum likelihood (ML) tree of classical major histocompatibility complex β chain (MHCIIβ) genes]. Fig. S14 – [Expansion of non-classical MHCIIβ (DMB) genes in duck]. Fig. S15 – [Multiple sequence alignment of classical MHCIIβ proteins]. Fig. S16 – [Multiple sequence alignment of DMB proteins]. Fig. S17 – [Maximum likelihood tree of NKC genes]. Fig. S18 – [Multiple sequence alignment of duck, chicken and human NKRP1 genes]. [file 12915_2024_1817_MOESM2_ESM.docx]

## Supplementary Figures


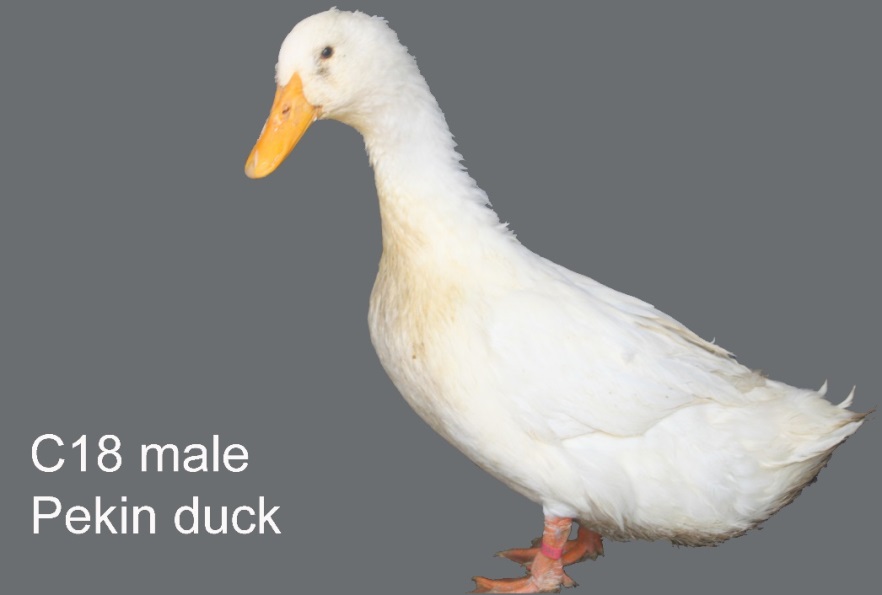


**Fig. S1** A healthy adult male Pekin duck (Anas platyrhynchos) which was used in this study for genome assembly.

**
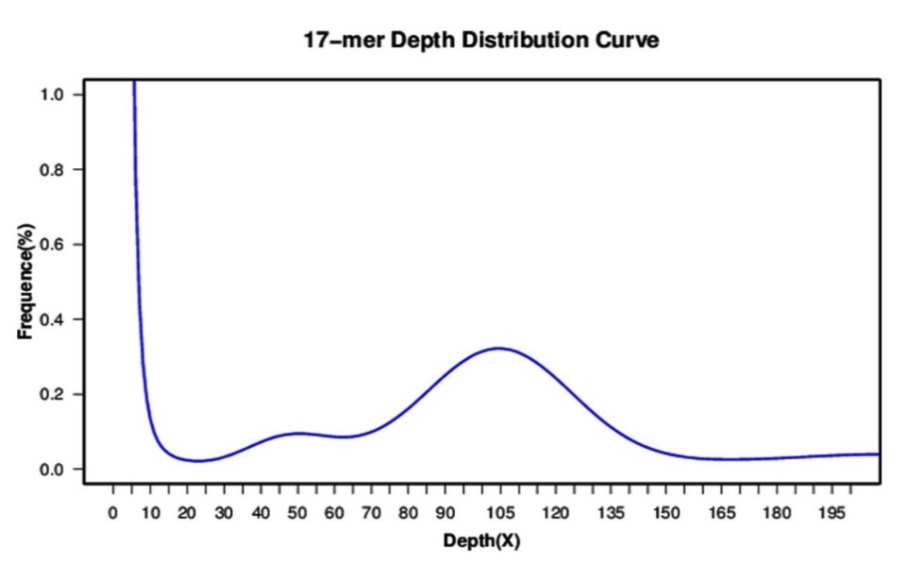
Fig. S2** 17-mer distribution curve. K-mer depth as x axis, the frequency of K-mer as y axis. The 7-mer distribution curve is an abnormal Poisson distribution which present a bimodal distribution. The peaks are near the 54x and 109x respectively.

**
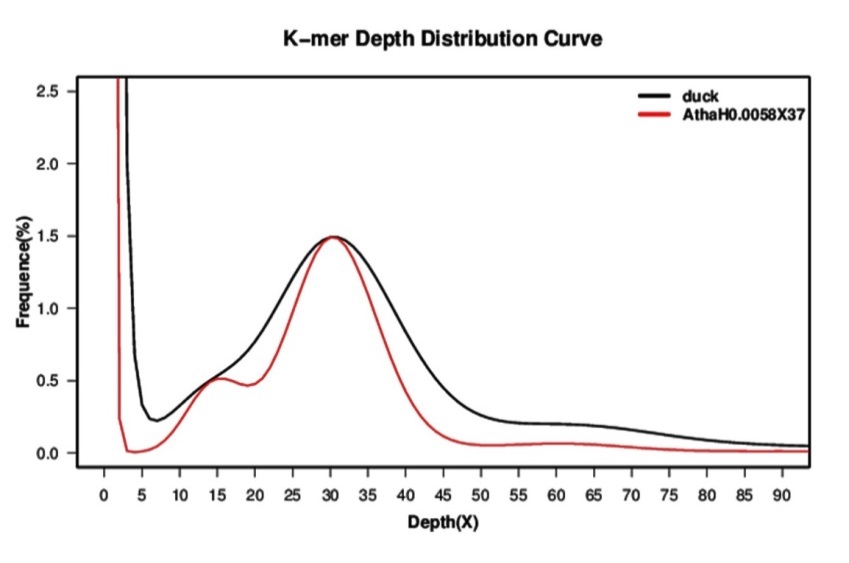
Fig. S3** K-mer depth distribution curve. K-mer depth as x axis, the frequency of K-mer as y axis. The black curve is our genome, the red curve is the result of simulation using Arabidopsis genome. The heterozygosity of Arabidopsis (H) is 1.0%, the depth of (X) is 31, other species were inferred according to this standard.


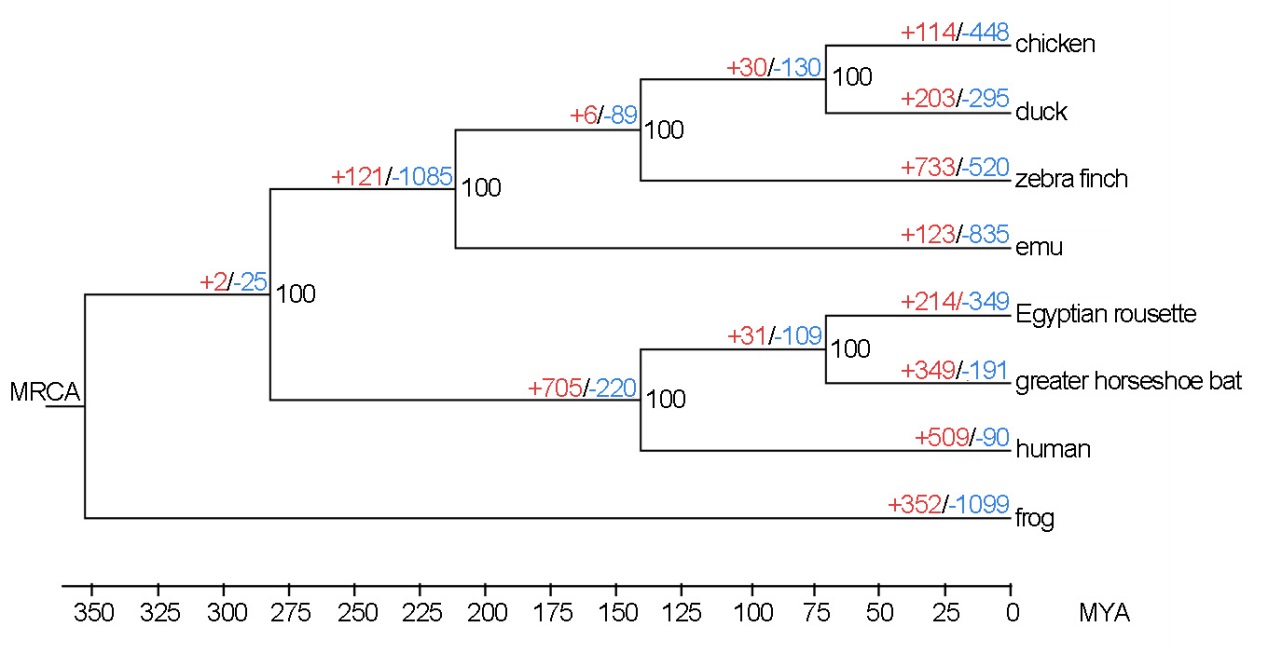
**Fig. S4** Gene expansion and contraction across a phylogenetic tree of eight species. We performed gene expansion and contraction analysis of 4,806 multiple-cope gene families using a maximum likelihood phylogenetic tree based on 1,800 single-copy gene families. Number of expanded and contracted gene families are in red and blue respectively. Bootstrap values (percentage of 1,000 bootstrap replicates) are shown in black on each clade. MRCA - most common ancestor. MYA - millions of years ago.


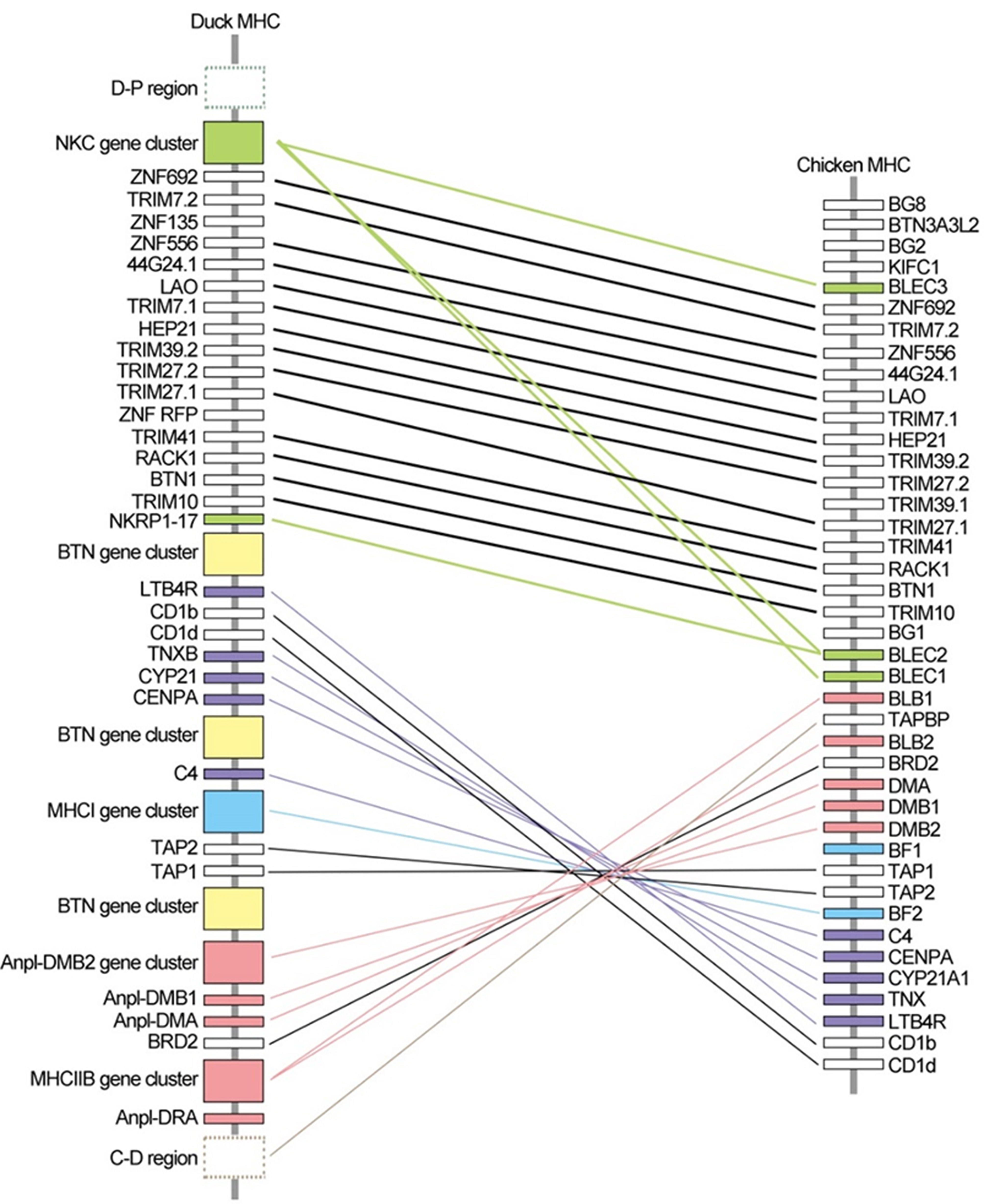


**Fig. S5** Conserved synteny of MHC gene map in duck and chicken. Homologs genes between species are linked by lines. Duck MHC is harbored in chromosome 17 (SKLA1.0). MHC region of chicken is located in chromosome 16 (chicken genome version: GCF_000002315.6). *MHCI*, *MHCII*, *MHCIII*, *BTN* and *NKC* gene family are colored in blue, pink, purple, yellow and green, respectively. The green and brown dotted boxes represent D-P and C-D regions respectively.


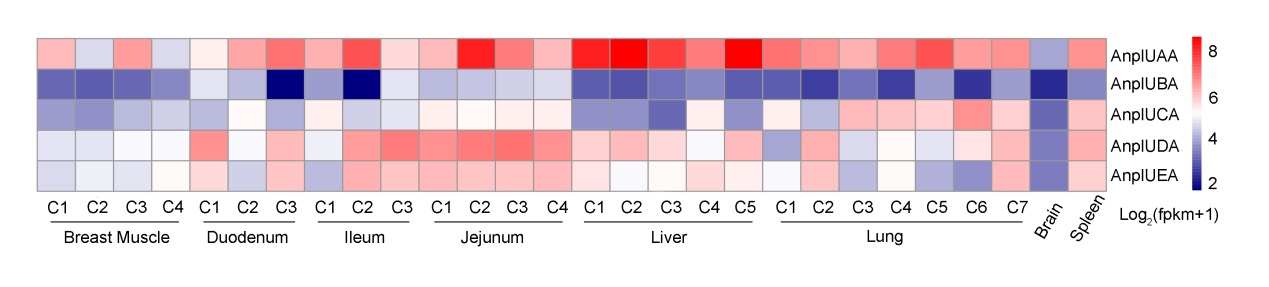
**Fig. S6** Expressional profile of duck *MHCIα* genes in eight tissues. The heatmap is based on the data provided in Additional file 3: Data S2 using the R packages ggplot2 (http://had.co.nz/ggplot2/) and pheatmap (https://cran.r-project.org/web/packages/pheatmap/).


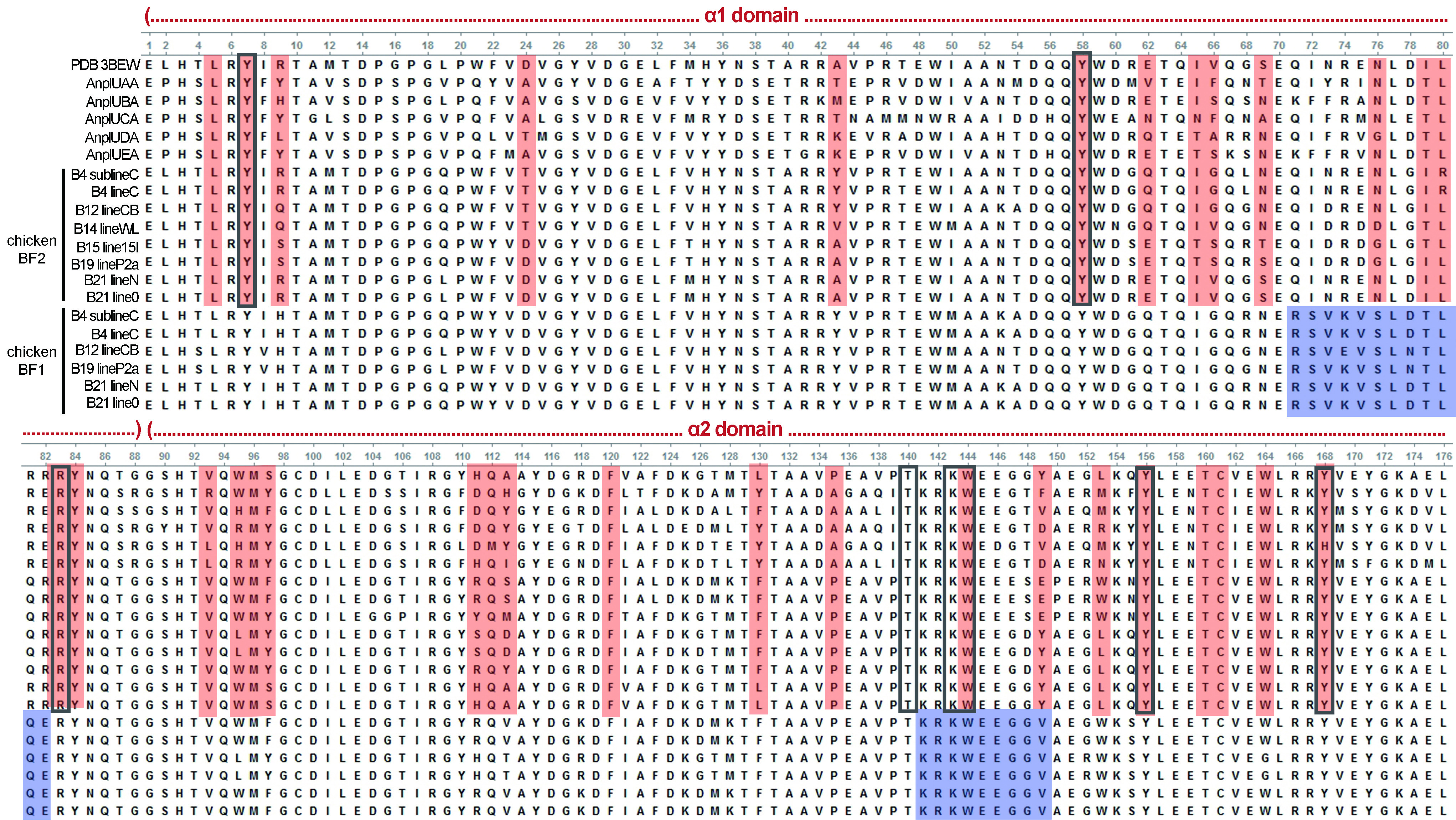

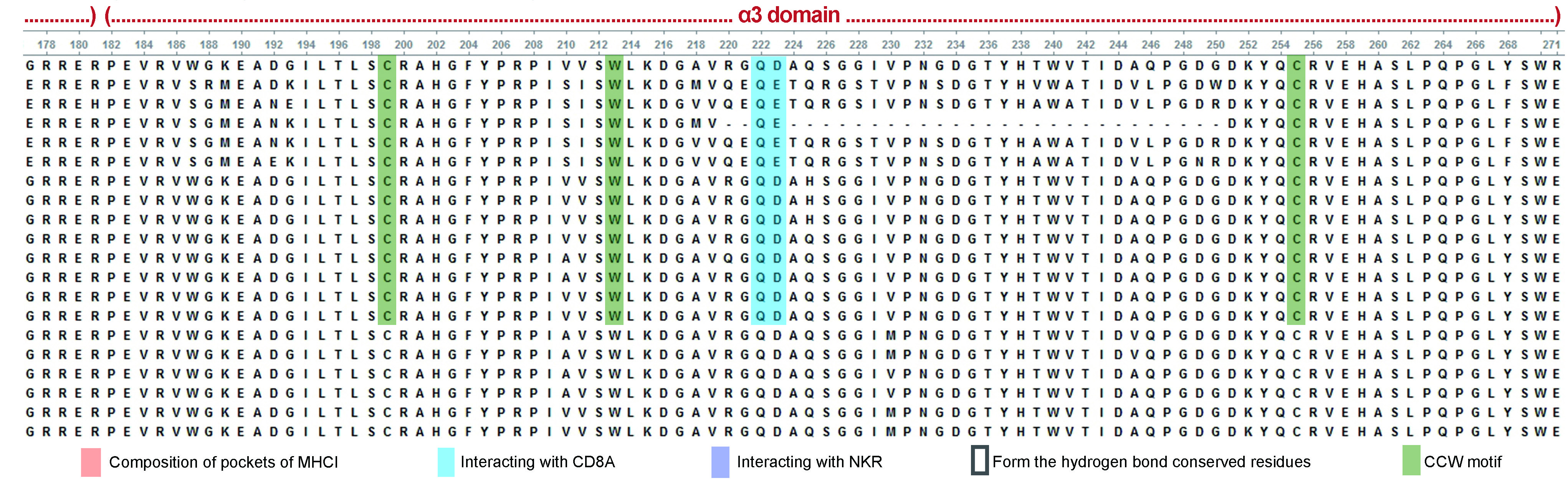


**Fig. S7** Multiple sequence alignment of MHCIα proteins. Duck MHCIα proteins (AnplUAA, AnplUBA, AnplUCA, AnplUDA, and AnplUEA) are from our data. Chicken MHCIα proteins (BF1 and BF2) were downloaded from the NCBI website. B4, B12, B14, B15, B19 and B21 represent *MHCIα* genes of different chicken lines. Protein sequence of chicken MHC Class I Haplotype B21 was set as reference sequence with PDB number of 3BEW. Sequence alignment was performed using the Prank software (version 140603) under parameters of “AA” model with 1,000 iterations. Protein sequence domains were predicted using the INTERPROSCAN (http://www.edi.ac.uk/interpro/) software with defaults parameters. According to previous researches in human, chicken and duck, we have predicted and marked a series of functionally important residues.


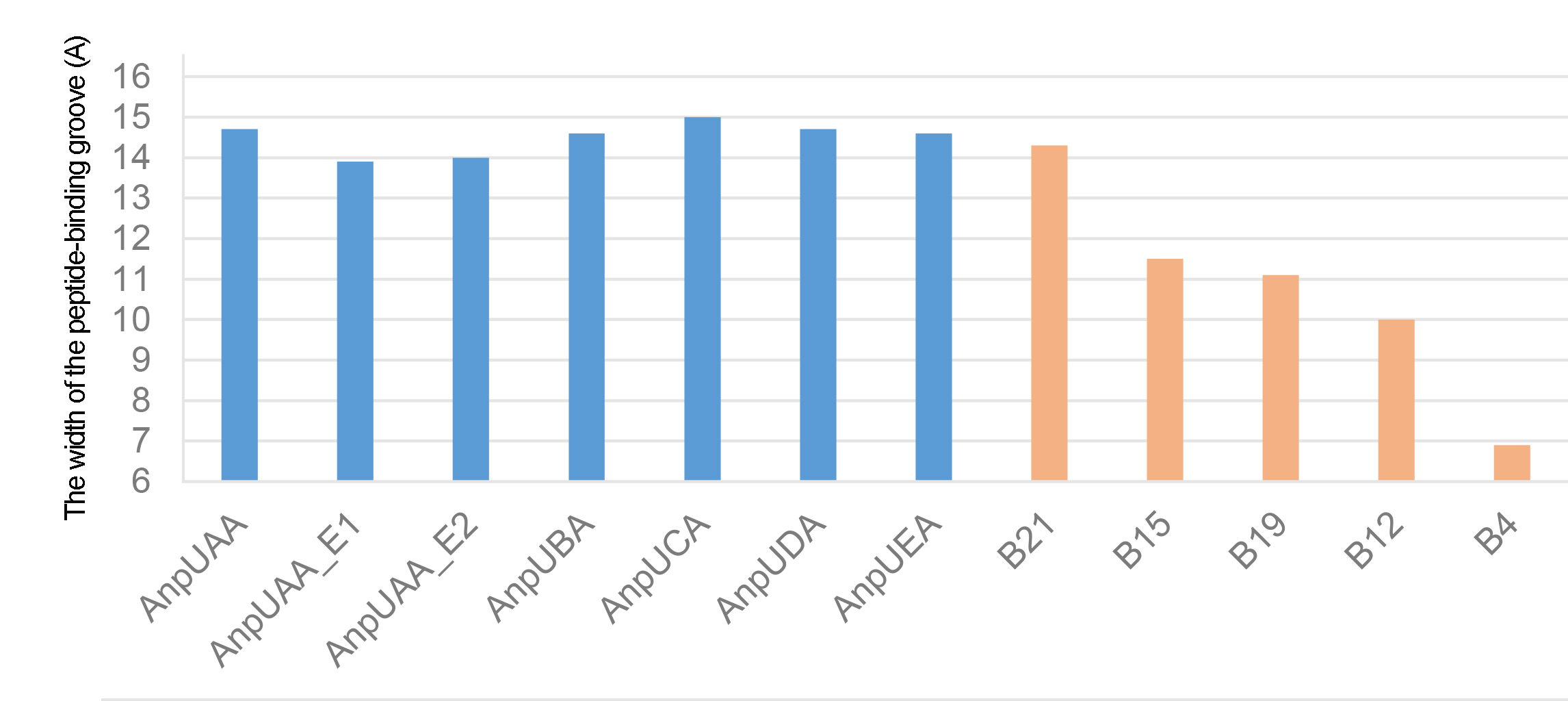


**Fig. S8** Opening size of the peptide-binding pocket of duck and chicken MHCIα proteins. Duck has five *MHCIα* genes (*AnplUAA*, *AnplUBA*, *AnplUCA*, *AnplUDA* and *AnplUEA*). AnpUAA_E1 and AnpUAA_E2 are experimentally-determined structure of UAA molecules (PDB code 5GJX and 5GJY). Chicken has only one *MHCIα* gene (BF2). B21, B19, B15, B12 and B4 represent *BF2* genes in different chicken lines. Structures of chicken BF2 gene in line B4 (PDB: 4E0R), B21 (PDB: 3BEV), B12 (PDB: 5YMV), B19 (PDB: 7WBG), and B15 (PDB: 6IRL) were downloaded from the PDD database (<https://www.rcsb.org/>). Structures of duck MHCIα (AnplUAA to AnplUEA) were made by point mutation and optimized by the Discovery Studio 2019 according to duck MHCIα protein (PDB:5GJX) respectively.


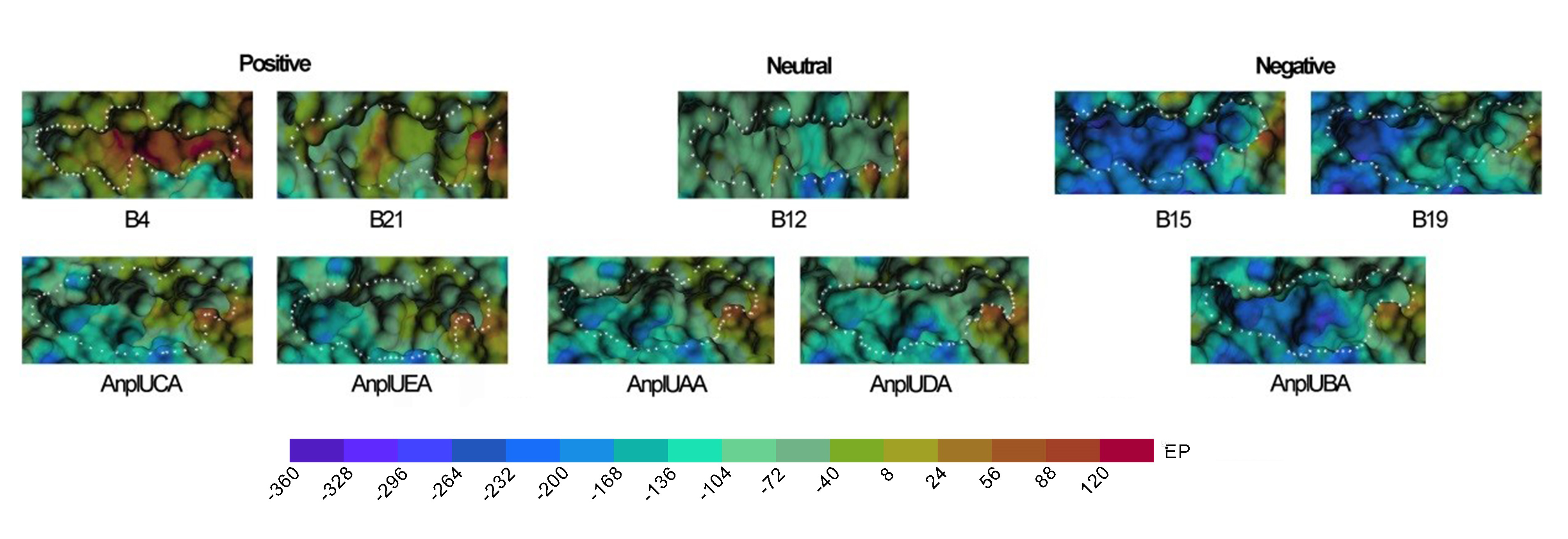
**Fig. S9** Electrostatic potential of peptide-binding pocket of duck and chicken MHCIα proteins. Peptide binding pockets of MHCIα proteins are circled with a while line. Map and data were generated by the SYBYL software (version X2.1.1).


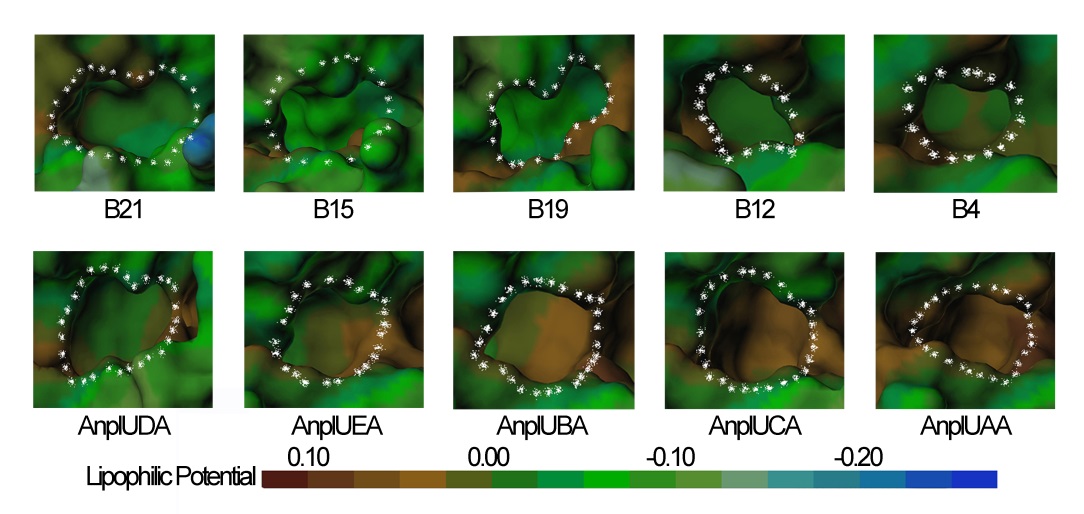


**Fig. S10** Lipophilic potential of pocket B of duck and chicken MHCIα proteins. Hydrophilic areas are colored in blue and lipophilic areas are colored in brown. The B pocket of MHCIα proteins is circled using a while line. Map and data were generated by the SYBYL software (version X2.1.1).


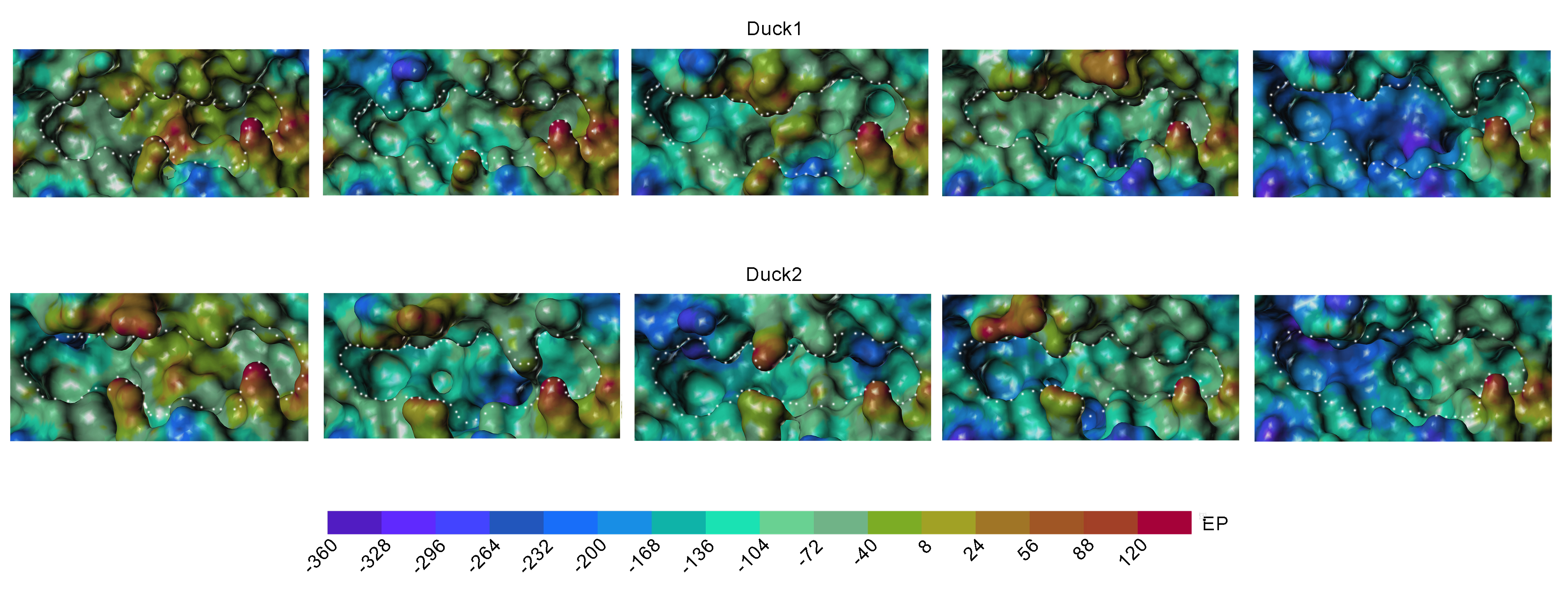
**Fig. S11** Electrostatic potential of the peptide-binding pocket of MHCIα proteins in another two ducks. Peptide-binding pockets of MHCIα proteins are circled with a while line. Map and data were generated by the SYBYL software (version X2.1.1). MHCIα proteins of duck 1 is retrieved from a published paper. MHCIα proteins of duck 2 is from our laboratory.


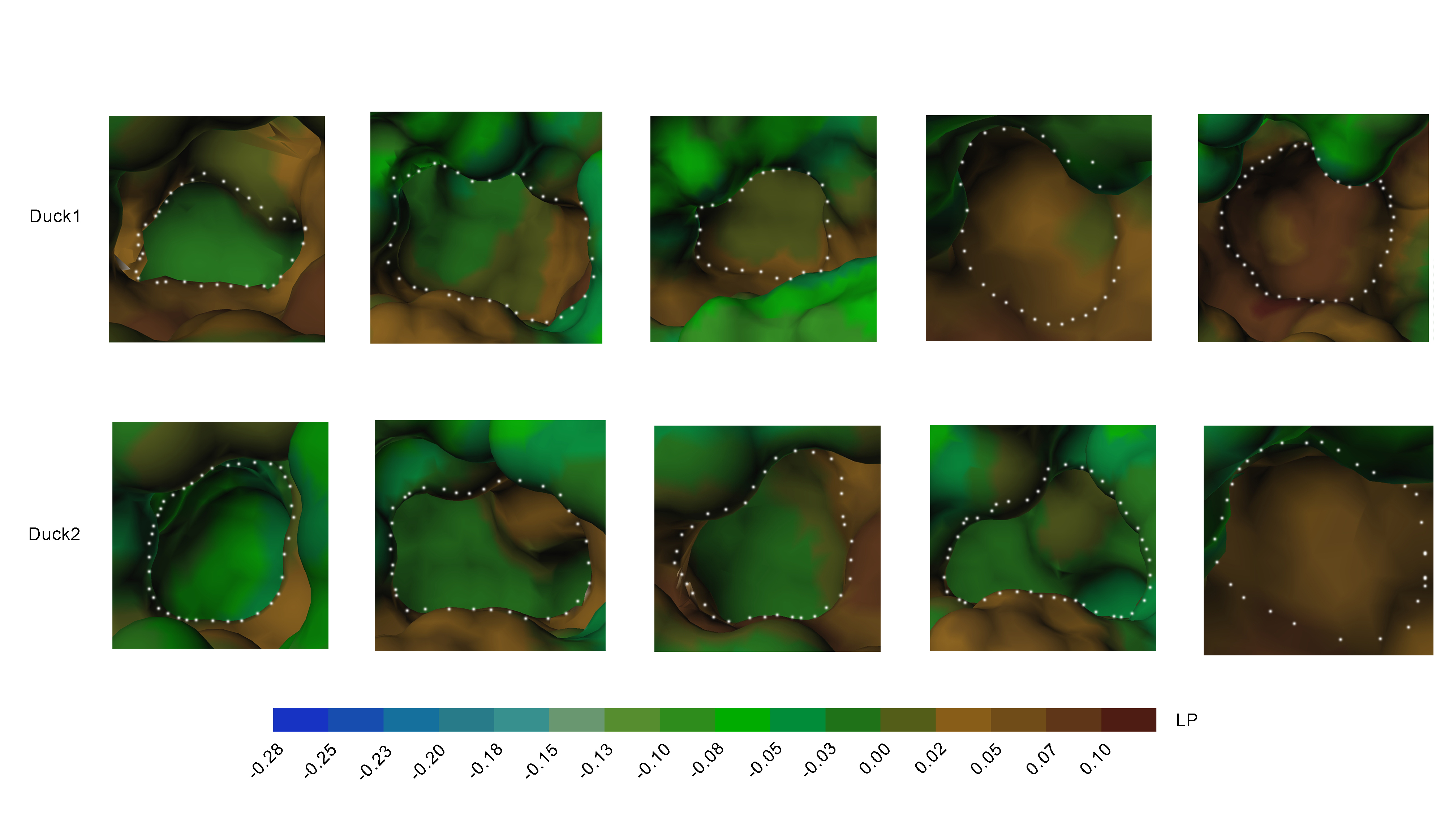


**Fig. S12** Lipophilic potential of pocket B of MHCIα proteins in another two ducks. Hydrophilic areas are colored in blue and lipophilic areas are colored in brown. The B pocket of MHCIα proteins is circled using a while line. Map and data were generated by the SYBYL software (version X2.1.1). MHCIα proteins of duck 1 is retrieved from a published paper. MHCIα proteins of duck 2 is from our laboratory.


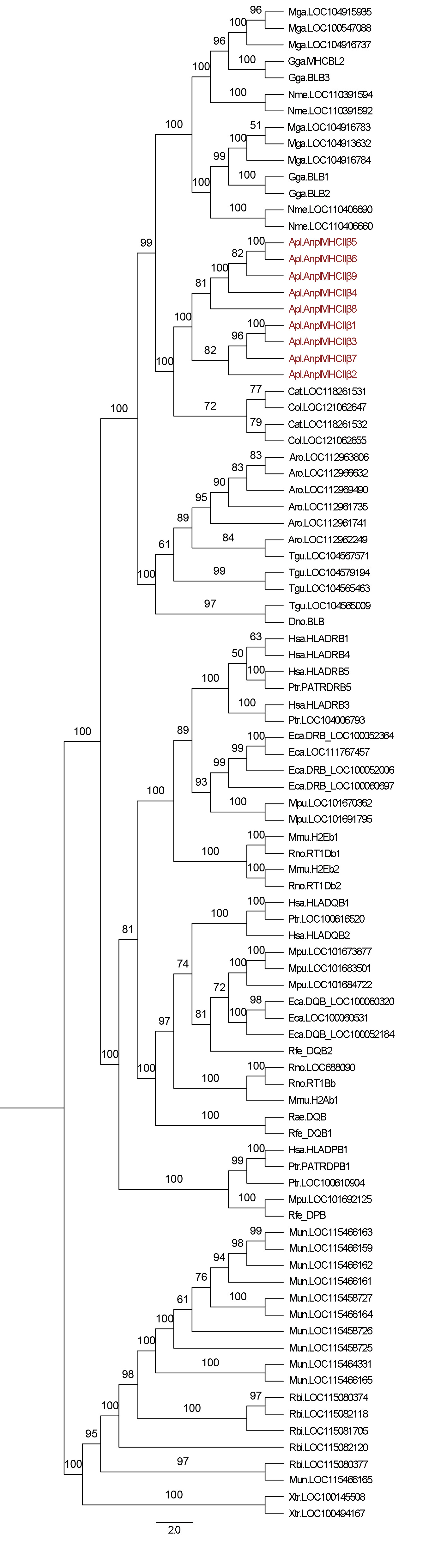


**Fig. S13** Maximum likelihood (ML) tree of classical major histocompatibility complex β chain (*MHCIIβ*) genes. This is the full ML tree of Fig. 4a with all expanded and collapsed clades shown. This ML tree was generated using 92 CDS sequences from 3 amphibians (tropical clawed frog, Xenopus tropicalis; caecilians, Microcaecilia unicolor; two-lined caecilian, Rhinatrema bivittatum), 8 mammalians (human, Homo sapiens; chimpanzee, Pan troglodytes; horse, Equus caballus; domestic ferret, Mustela putorius furo; mouse, Mus musculus; Norway rat, Rattus norvegicus; greater horseshoe bat, Rhinolophus ferrumequinum; Egyptian rousette, Rousettus aegypti- acus), and 9 birds (duck, Anas platyrhynchos; turkey, Meleagris gallopavo; chicken, Gallus gallus; helmeted guineafowl, Numida meleagris; black swan, Cygnus atratus; mute swan, Cygnus olor; Okarito brown kiwi, Apteryx rowi; white-throated tinamou, Tinamus guttatus; emu, Dromaius novaehollandiae). Duck MHCIIβ CDSs were from our SKLA1.0 genome and others were downloaded from the NCBI website (https://www.ncbi.nlm.nih.gov/). Multiple sequence alignment was performed using the Prank software (version 140603) under the “DNA” model with 1,000 iterations. The ML tree was built using the IQ-tree software (version 1.6.5) with defaults of 1,000 replications after auto-selection of the best model using “-m TEST” based on the Bayesian information criterion (BIC) score, and was visualized using the Figtree program (version 1.42). Bootstrap values of 1,000 bootstrap replicates are marked on branches.


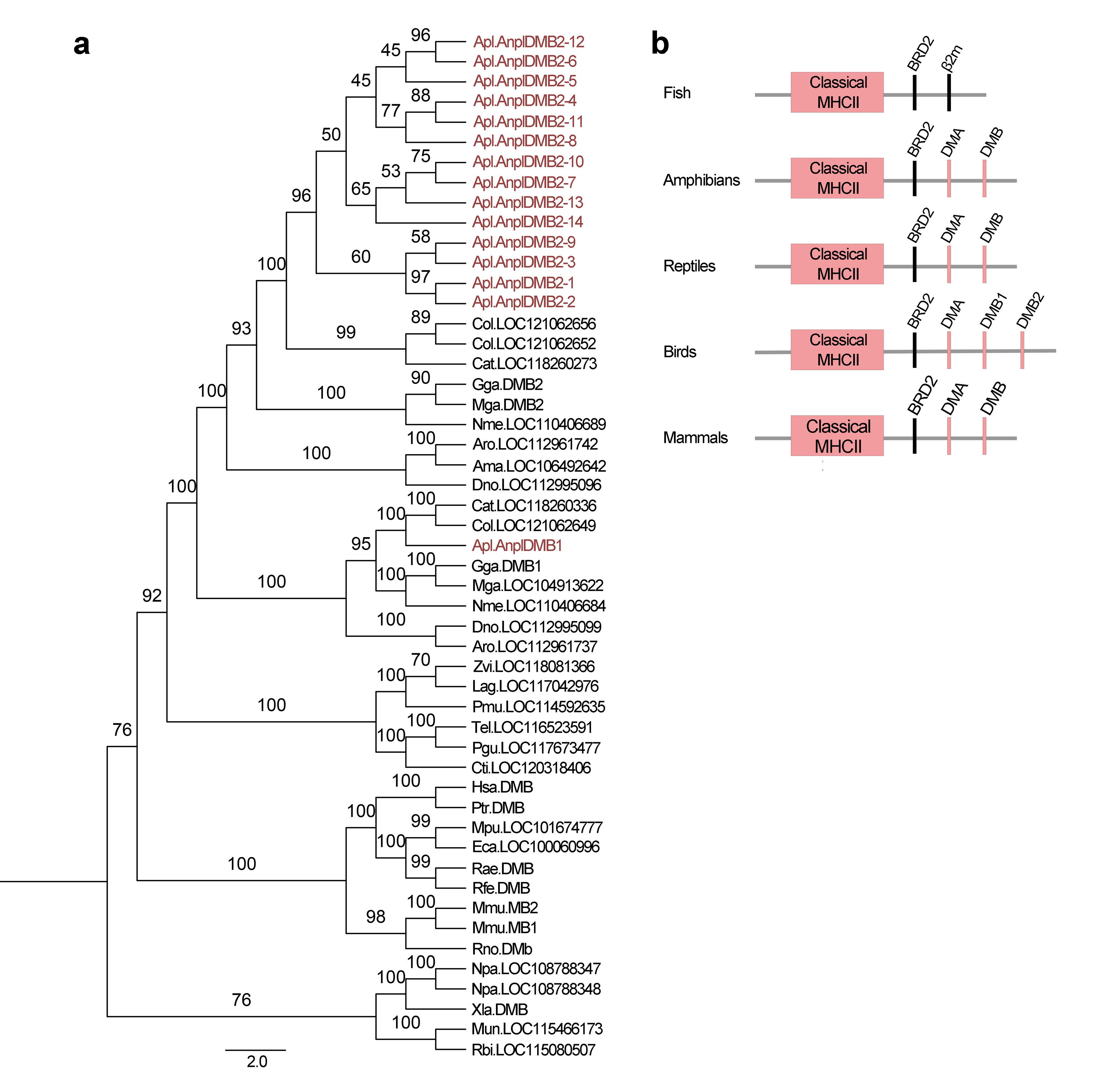


**Fig. S14** Expansion of non-classical MHCIIβ (*DMB*) genes in duck. **a.** Maximum likelihood tree of *DMB* genes. This is the tree displayed in Fig. 4b with all collapsed clades expanded. This ML tree was generated using 51 CDS sequences from 4 amphibians (Altirana parkeri, Nanorana parkeri; African clawed frog, Xenopus laevis; caecilians, Microcaecilia unicolor; two-lined caecilian, Rhinatrema bivittatum), 8 mammalians (human, Homo sapiens; chimpanzee, Pan troglodytes; domestic ferret, Mustela putorius furo; horse, Equus caballus; Egyptian rousette, Rousettus aegyptiacus; greater horseshoe bat, Rhinolophus ferrumequinum; mouse, Mus musculus; Norway rat, Rattus norvegicus), 6 Reptiles (common lizard, Zootoca vivipara; Sand lizard, Lacerta agilis; Common wall lizard, Podarcis muralis; Western terrestrial garter snake, Thamnophis elegans; Corn Snake, Pantherophis guttatus; Tiger rattlesnake, Crotalus tigris) and 9 birds (duck, Anas platyrhynchos; mute swan, Cygnus olor; black swan, Cygnus atratus; chicken, Gallus gallus; turkey, Meleagris gallopavo; helmeted guineafowl, Numida meleagris; Okarito brown kiwi, Apteryx rowi; brown kiwi, Apteryx mantelli mantelli; emu, Dromaius novaehollandiae). Duck CDS sequences were retrieved from our SKLA1.0 genome and others were downloaded from NCBI website (https://www.ncbi.nlm.nih.gov/). Multiple sequence alignment was performed using the Prank software (version 140603) under the “DNA” model with 1,000 iterations. The ML tree was built using the IQ-tree software (version 1.6.5) with the default parameters of 1,000 replications after auto-selection of the best model using “-m TEST” based on the Bayesian information criterion (BIC) score, and was visualized using the Figtree program (version 1.42). Bootstrap value of 1,000 bootstrap replicates were marked on branches. **b.** Syntenic analysis of the *MHCII* gene family. This result shows that birds have duplicated *DMB* gene and generated two *DMB* members namely *DMB1* and *DMB2*.

**
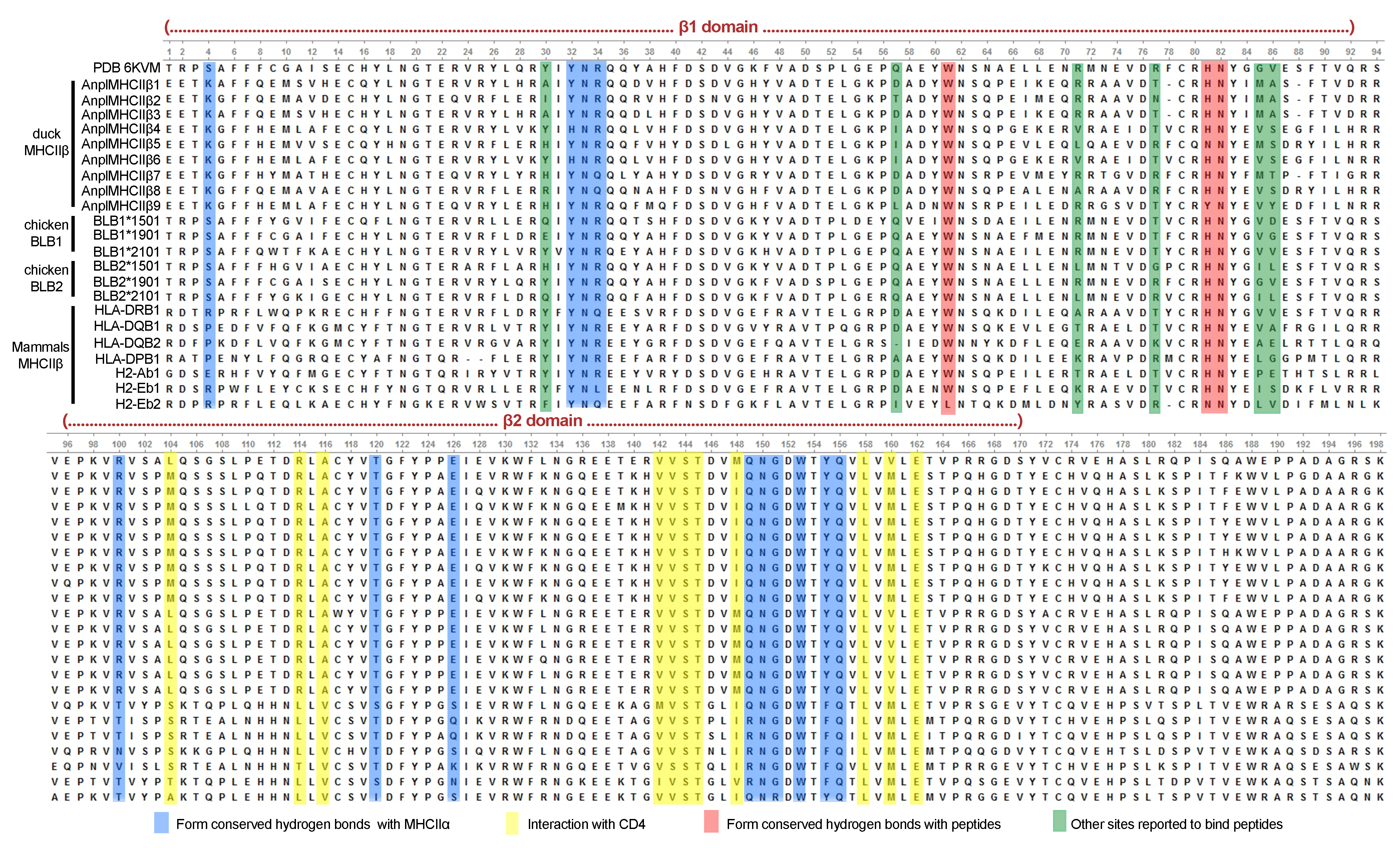
**

**Fig. S15** Multiple sequence alignment of classical MHCIIβ proteins. Duck major histocompatibility complex β chain (MHCIIβ) proteins were retrieved from our SKLA1.0 genome. Chicken MHCIIβ (BLB1 and BLB2) and mammalian MHCIIβ protein sequences were downloaded from the NCBI website (https://www.ncbi.nlm.nih.gov/). Alignment of protein sequences was performed using the Prank software (version 140603) under the “AA” model with 1,000 iterations. Domain structures of proteins were predicted using the INTERPROSCAN (http://www.edi.ac.uk/interpro/). “-” denotes gap area. According to gene functional studies in human and chicken, we have predicted key residues in duck MHCIIβ proteins.


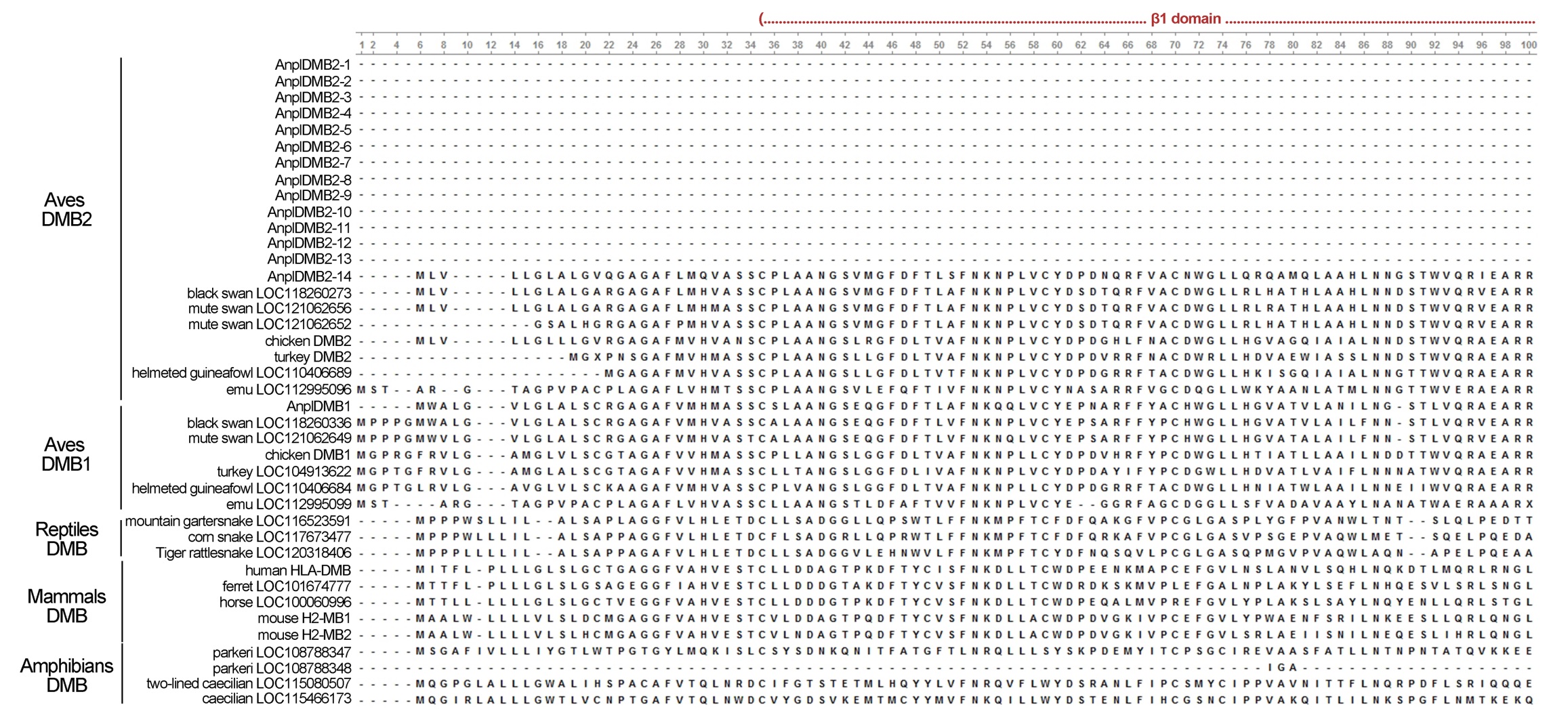


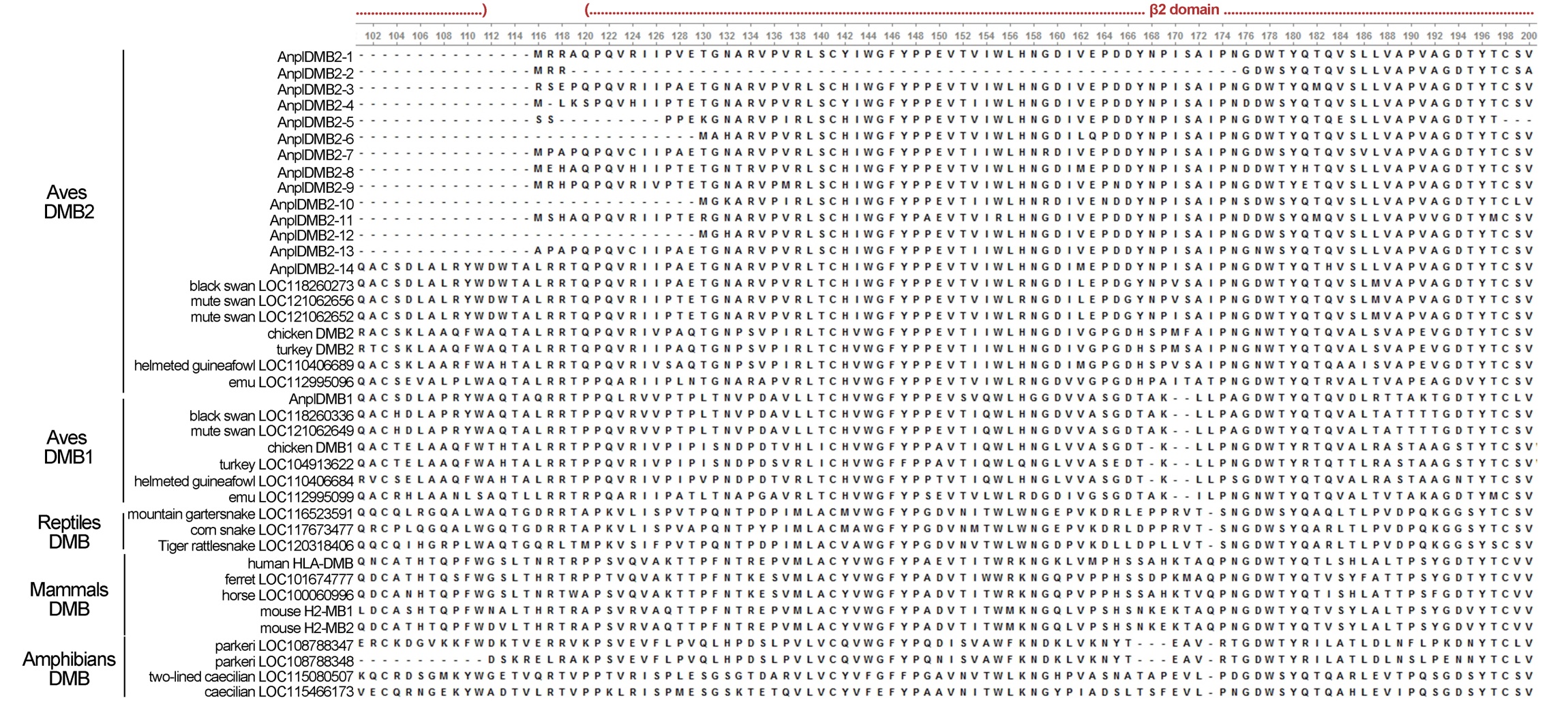


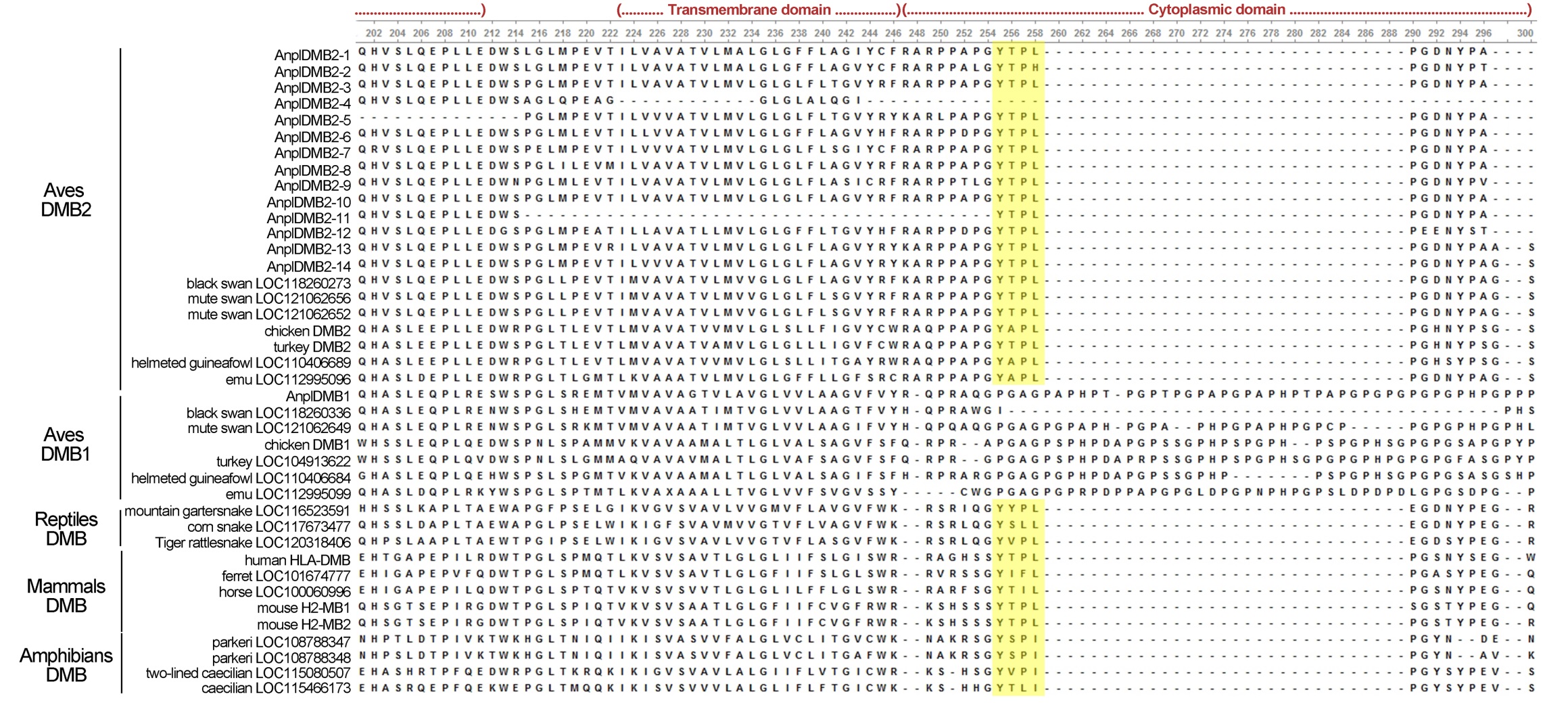


**Fig. S16** Multiple sequence alignment of DMB proteins. Duck DMBs protein sequences were retrieved from our SKLA1.0 genome. Other DMB protein sequences were downloaded from the NCBI website (https://www.ncbi.nlm.nih.gov/). Protein structures were predicted using the INTERPROSCAN (http://www.edi.ac.uk/interpro/) with default parameters. Multiple sequence alignments were performed using the Prank software (version 140603) under the “AA” model with 1,000 iterations. The cytoplasmic YTPL targeting motif is colored in yellow. Avian DMB1s lost this motif. “-” denotes gap area.


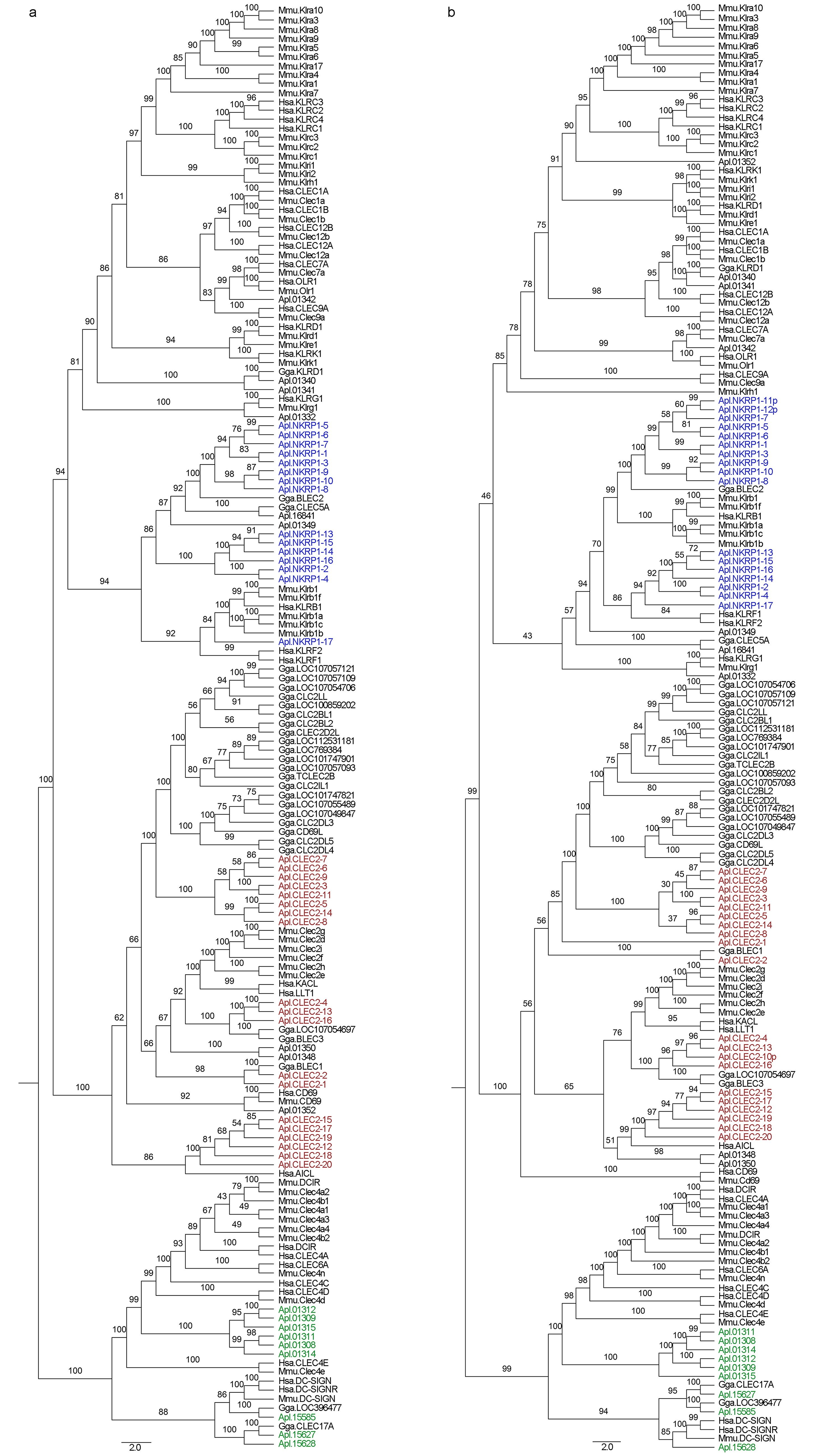


**Fig. S17** Maximum likelihood tree of *NKC* genes. Duck sequences were retrieved from our SKLA1.0 genome. The other sequences were downloaded from the NCBI website (https://www.ncbi.nlm.nih.gov/). Multiple sequence alignment was performed using the Prank software (version 140603) under the “DNA” model with 1,000 iterations. The ML tree was build using the IQ-tree software (version 1.6.5) with defaults of 1,000 replications after auto-selection of the best model using “-m TEST” based on the Bayesian information criterion (BIC) score. The tree was visualized using the Figtree program (version 1.42). Bootstrap values in proportion of 1,000 replicates are marked on branches. Duck natural killer cell receptor *NKRP1* and its ligand *CLEC2* are colored in blue and red respectively. Green represents duck DC-specific C-type lections. **a.** Maximum likelihood tree (ML) of *NKC* genes. This is the full tree displayed in Fig. 5a with all collapsed clades expanded. This ML tree was generated using 29 human, 50 mouse, 29 chicken and 52 duck CDS sequences. **b** Maximum likelihood tree (ML) of *NKC* genes including three duck pseudogenes (*NKRP1-11p*, *NKRP1-12p* and *CLEC2-10p*).


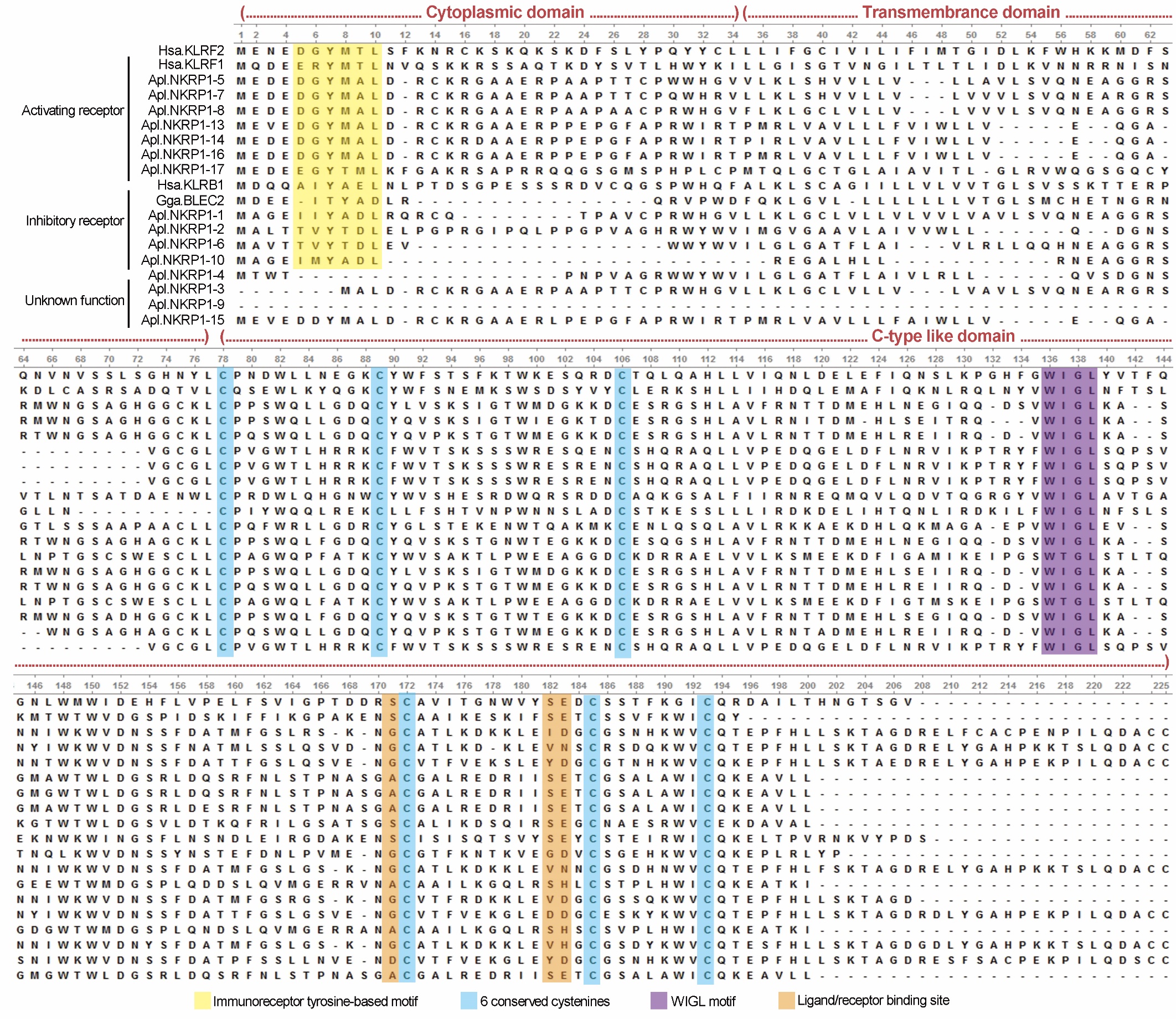


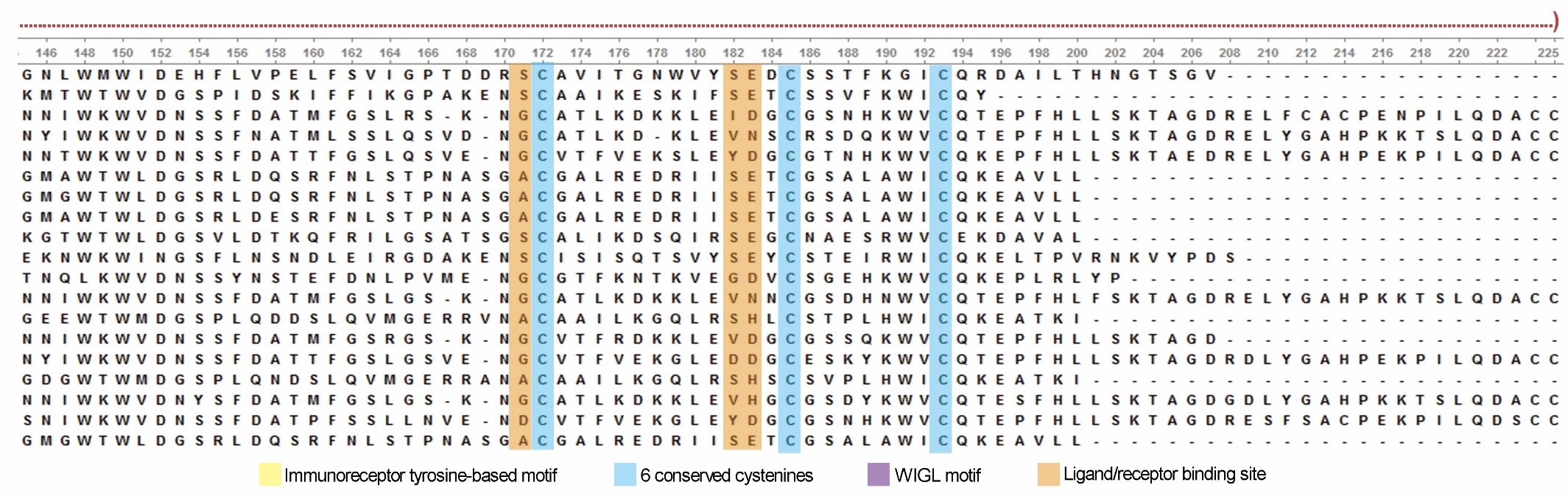


**Fig. S18** Multiple sequence alignment of duck, chicken and human *NKRP1* genes. Duck NKRP1 protein sequences were retrieved from our SKLA1.0 genome. Other NKRP1 proteins were downloaded from NCBI website (https://www.ncbi.nlm.nih.gov/). Protein structures were predicted using the INTERPROSCAN (http://www.edi.ac.uk/interpro/) with default parameters. Multiple sequence alignments were performed using the Prank software (version 140603) under the “AA” model with 1,000 iterations. “-” denotes gap. According to previous researches in chicken and human, we have predicted key residues in duck NKR-like genes.
