# Supplementary material for: A new chromosome-scale duck genome shows a major histocompatibility complex with several expanded multigene families
Source: BMC Biol. 2024 Feb 5;22:31. doi: 10.1186/s12915-024-01817-0 (PMC10845735; doi:10.1186/s12915-024-01817-0)
Supplement: Supplementary file 5 — Additional file 5: Figure S19- Figure S34. Fig. S19 – [Multiple sequence alignment of duck, chicken and human CLEC2 genes]. Fig. S20 – [Maximum likelihood (ML) tree of BTN genes]. Fig. S21 – [Expression profiles of duck BTN gene in eight tissues]. Fig. S22 – [Multiple sequence alignment of duck BTN proteins]. Fig. S23 – [Histopathological images of lung tissues from duck and chicken]. Fig. S24 – [Inflammatory cell infiltration in lung tissues of duck and chicken]. Fig. S25 – [Protein level of IFN-γ, IL-4, IL-6, IL-8 and TNF-α in duck (Top) and chicken (Bottom) plasma]. Fig. S26 – [Expressional profiles of inflammation-related genes in lung tissues of duck and chicken infected by A/chicken/Sheny/0606/2008 (SY/08) H5N1 virus]. Fig. S27 – [Antibody titer in ducks and chickens infected by the recombinant attenuated SY08ΔHA H5N1 virus]. Fig. S28 – [Expression of BCL6 and AICDA genes in spleen of ducks and chickens infected by the recombinant attenuated SY08ΔHA H5N1 virus and in control individuals]. Fig. S29 – [Multiple sequence alignment of CD8A proteins]. Fig. S30 – [Maximum likelihood tree of CD8As genes]. Fig. S31 – [Predicted structures of duck DM heterodimers]. Fig. 32 – [A proposed defense model to avian influenza virus in duck]. Fig. S33 – [Comparison of chromosome 17 (chr17) cmap and Bionano cmap]. Fig. S34 – [Hi-C matrix map of chromosome 17]. [file 12915_2024_1817_MOESM5_ESM.docx]

## Supplementary Figures


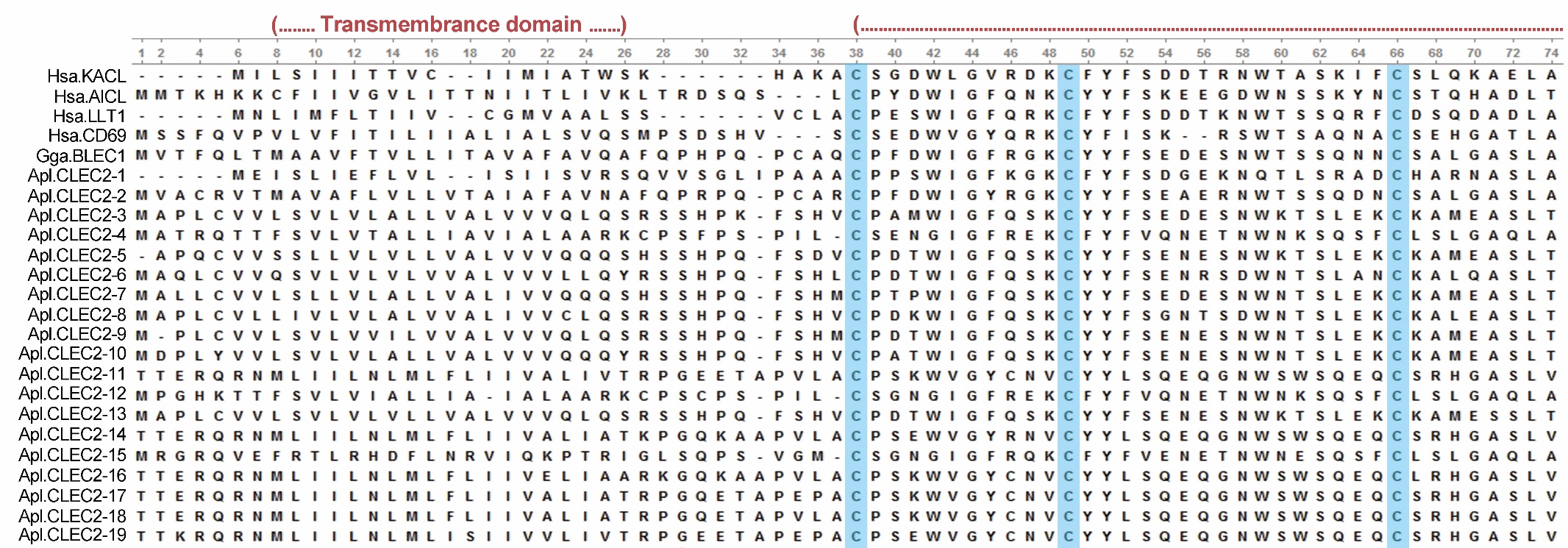


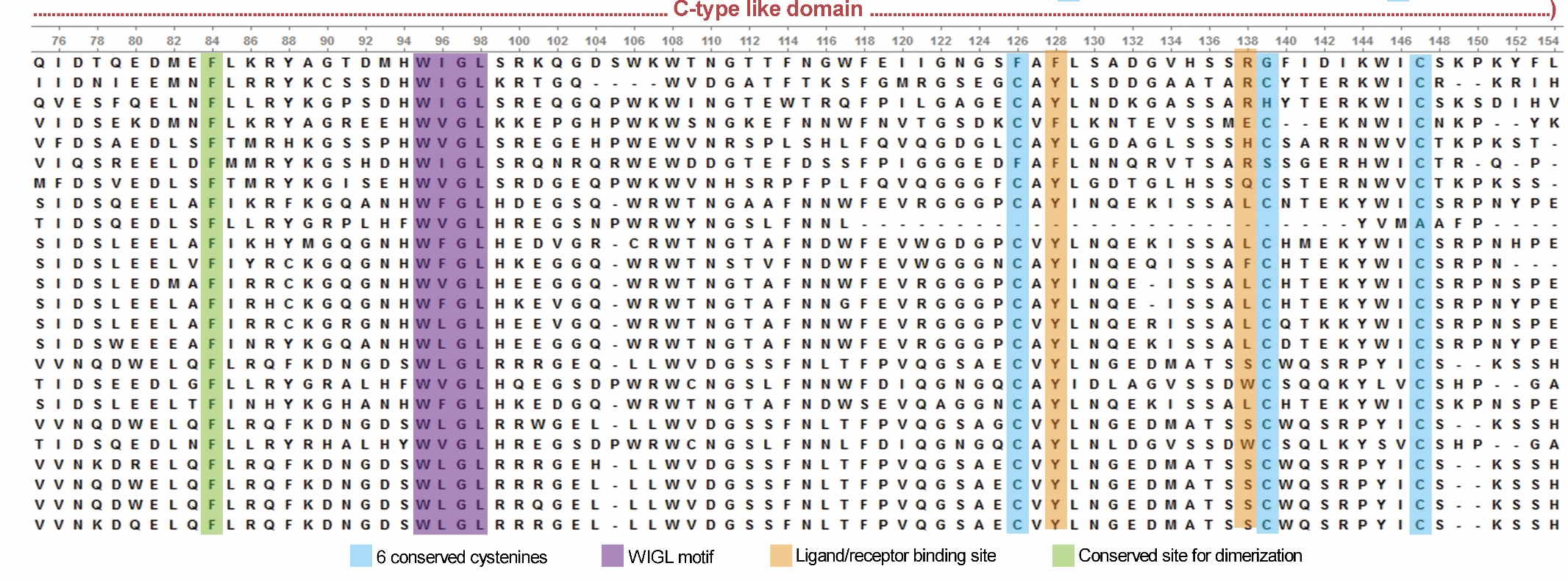


**Fig. S19** Multiple sequence alignment of duck, chicken and human *CLEC2* genes. Duck CLEC2 (like) protein sequences were retrieved from our SKLA1.0 genome. Other CLEC2 proteins were downloaded from the NCBI website (https://www.ncbi.nlm.nih.gov/). Protein structures were predicted using the INTERPROSCAN (http://www.edi.ac.uk/interpro/) with default parameters. Multiple sequence alignments were performed using the Prank software (version 140603) under the “AA” model with 1,000 iterations. “-” denotes gap.


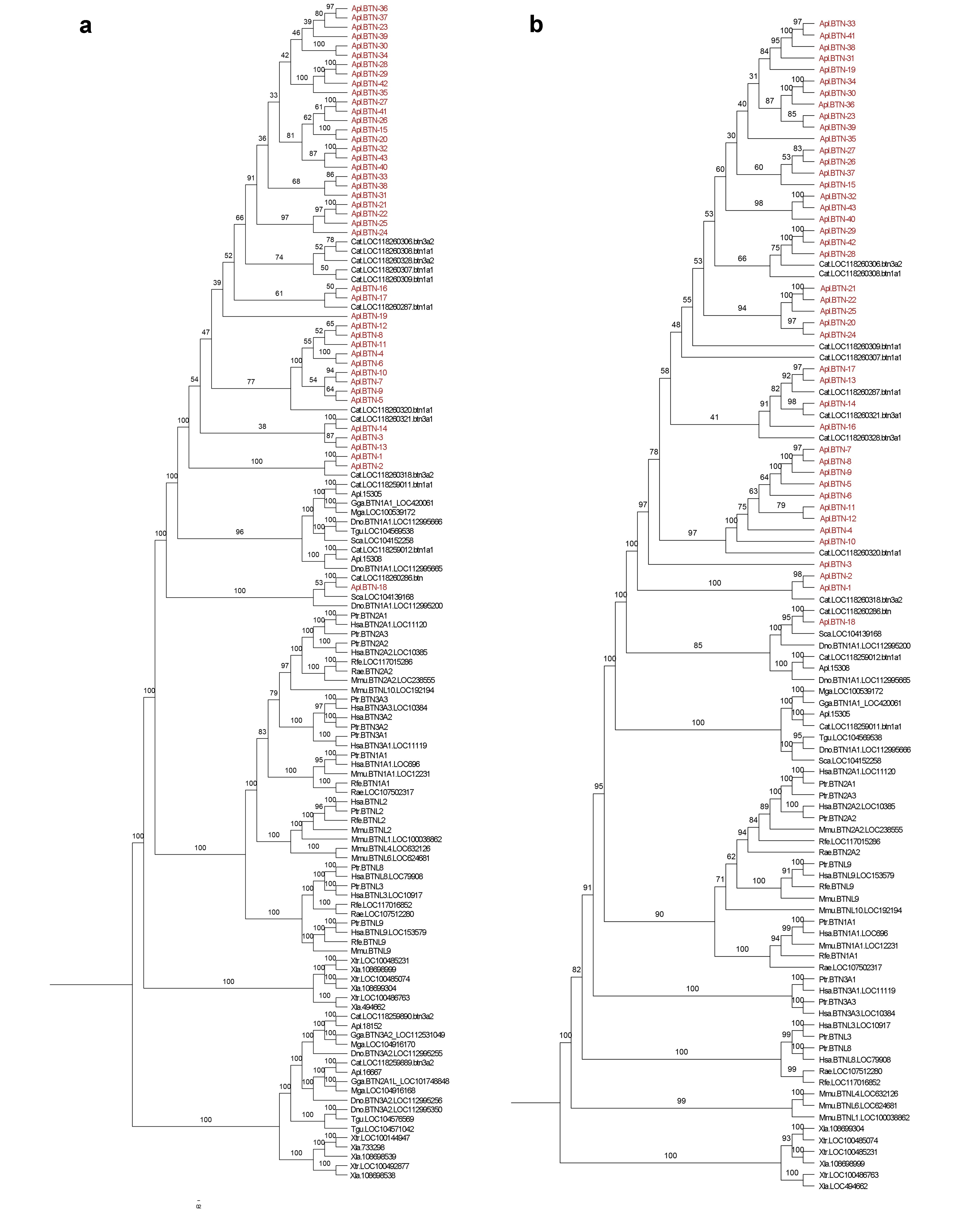


**Fig. S20** Maximum likelihood (ML) tree of *BTN* genes. Duck CDS sequences were taken from our SKLA1.0 genome and other sequences were downloaded from the NCBI website (https://www.ncbi.nlm.nih.gov/). ML trees were generated using 126 CDS sequences from 2 amphibians (African clawed frog, Xenopus laevis; tropical clawed frog, Xenopus tropicalis), 5 mammalians (chimpanzee, Pan troglodytes; human, Homo sapiens; Egyptian rousette, Rousettus aegyptiacus; greater horseshoe bat, Rhinolophus ferrumequinum; mouse, Mus musculus) and 7 birds (duck, Anas platyrhynchos; black swan, Cygnus atratus; chicken, Gallus gallus; turkey, Meleagris gallopavo; emu, Dromaius novaehollandiae; white-throated tinamou, Tinamus guttatus; ostrich, Struthio camelus australis). Multiple sequence alignment was performed using the Prank software (version 140603) under the “DNA” model with 1,000 iterations. ML trees were built using the IQ-tree software (version 1.6.5) with the defaults of 1,000 replications after auto-selection of the best model using “-m TEST” based on the Bayesian information criterion (BIC) score, and was visualized using the Figtree program (version 1.42). Bootstrap values in proportion of 1,000 replicates are marked on branches. Duck *BTN* genes of the MHC region are shown in red. **a.** ML tree based on full CDS sequences. **b.** ML tree based on CDS sequences of the B30.2 domain. B30.2 domains were annotated through the online analysis of conserved domains in the NCBI database, with defaults of CDD v.3.16 50369 PSSMs and E value of 0.01 in a concise model. Hsa.BTNL2, Ptr.BTNL2, Rfe.BTNL2, Mmu.BTNL2, Hsa.BTN3A2 and Ptr.BTN3A2 containing no B30.2 domain were not included in this ML tree.


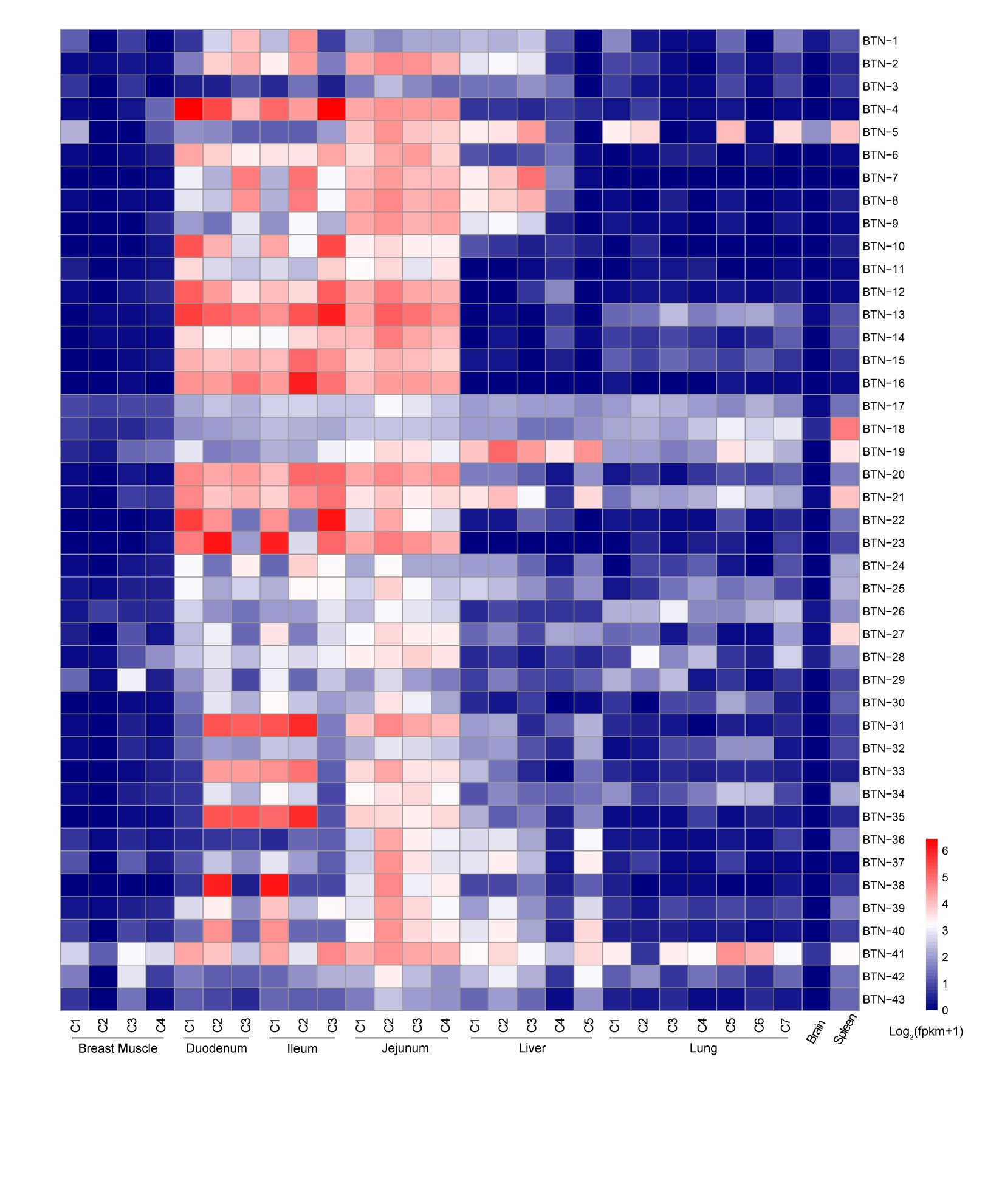


**Fig. S21** Expression profiles of duck *BTN* gene in eight tissues. The heatmap was generated based on data in Additional file 3: Data S2 using the R packages ggplot2 (http://had.co.nz/ggplot2/) and pheatmap (<https://cran.r-project.org/web/packages/pheatmap/>). Detailed sample information is in Additional file 3: Data S6.


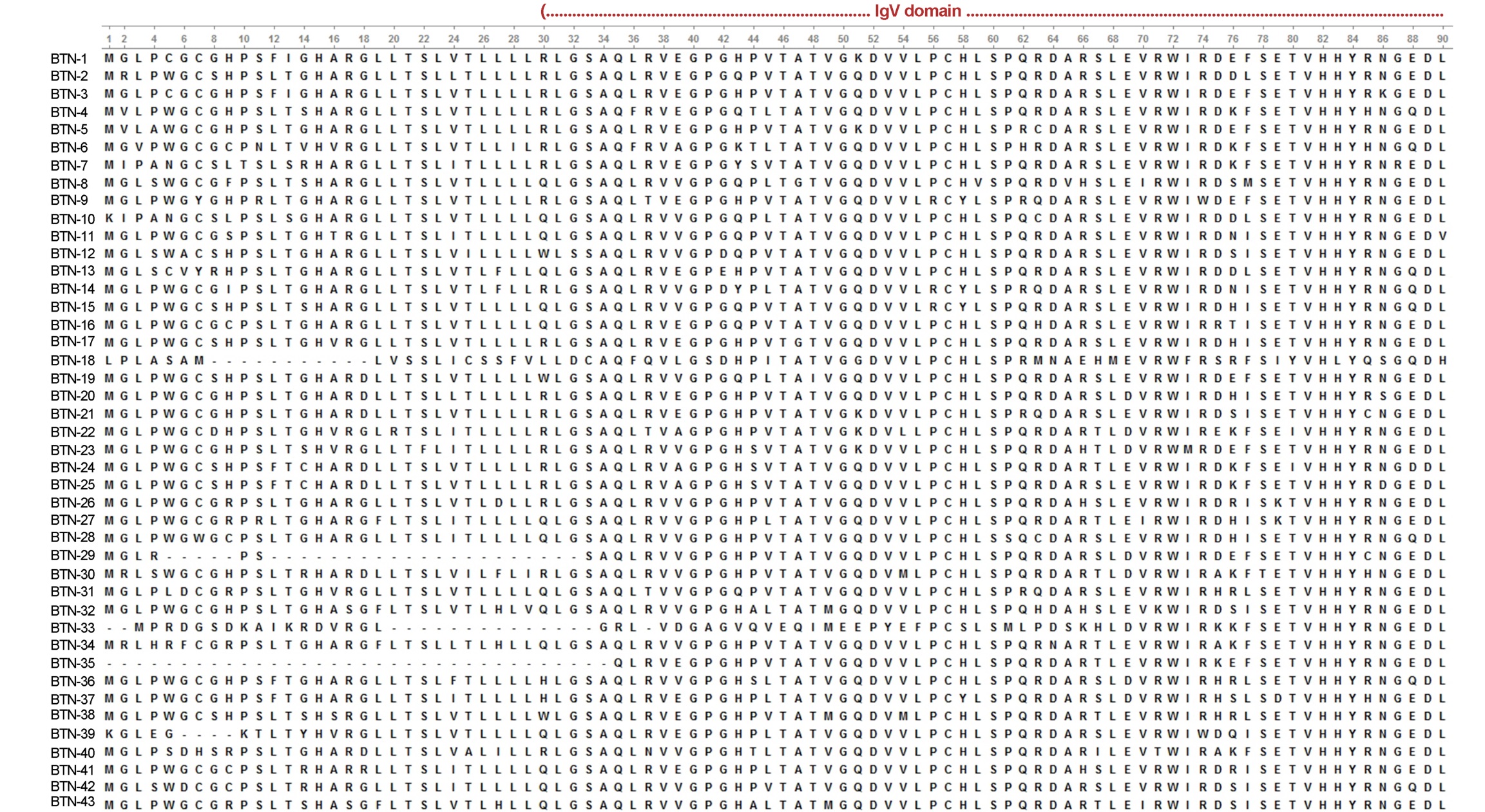


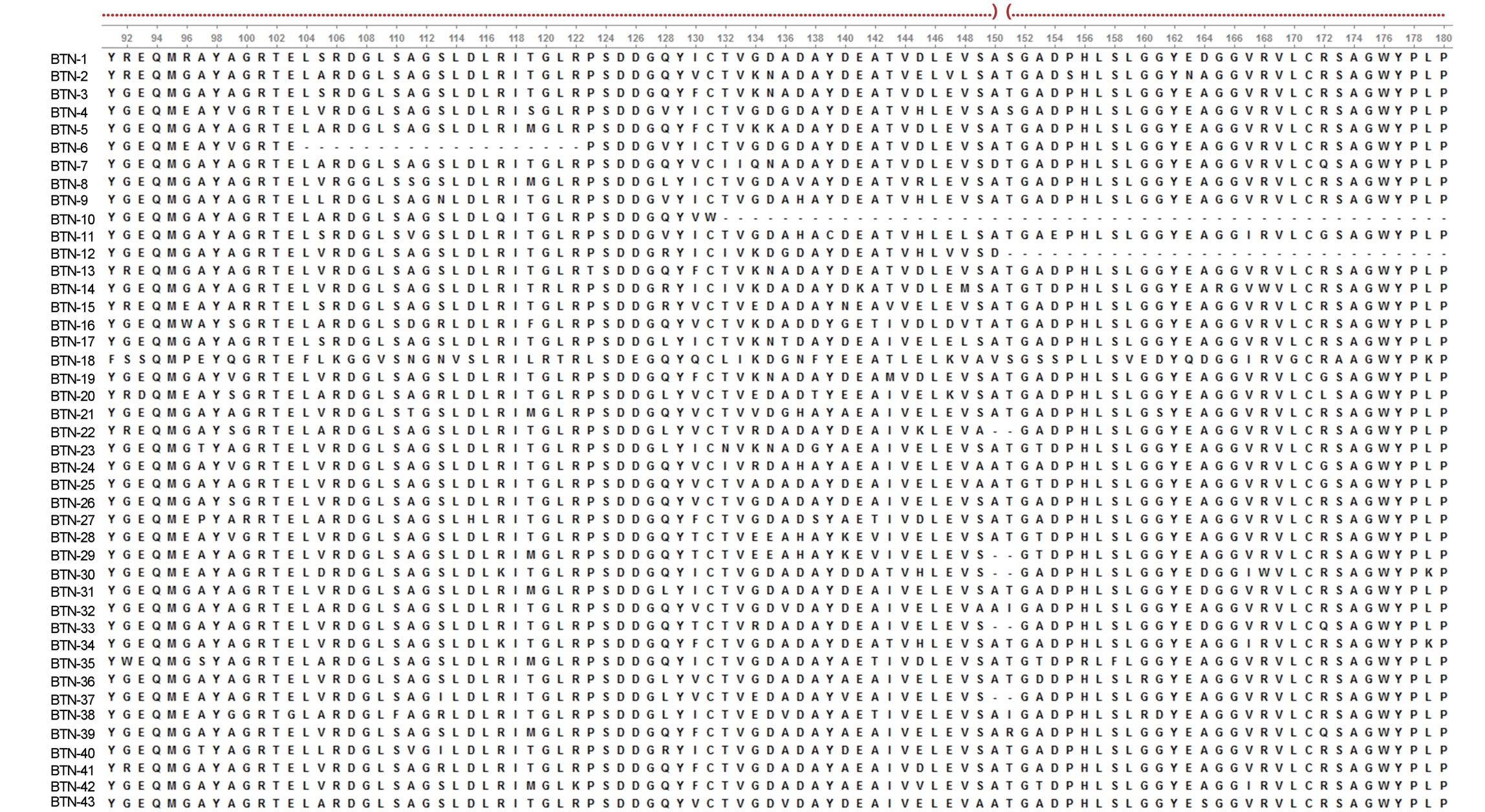


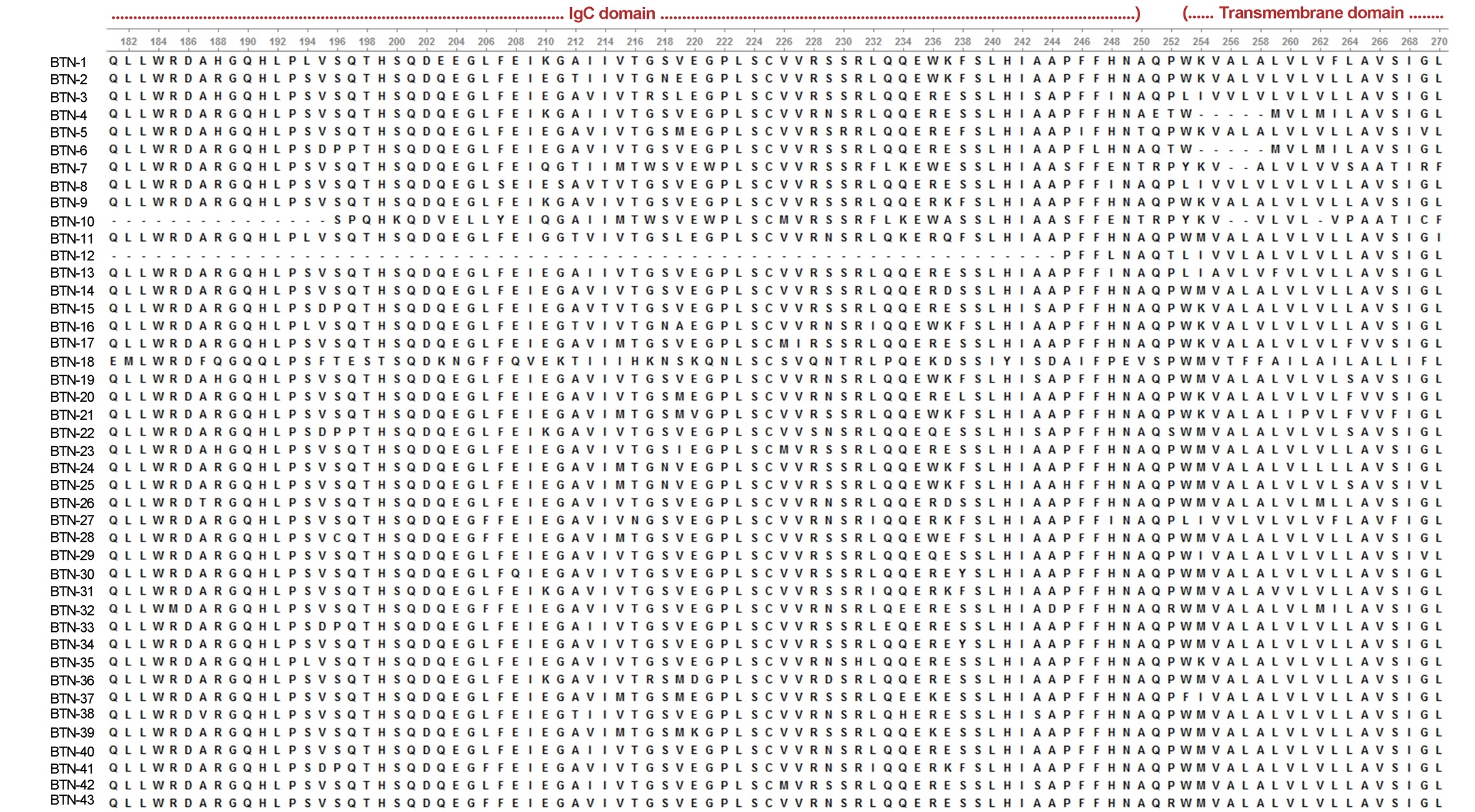


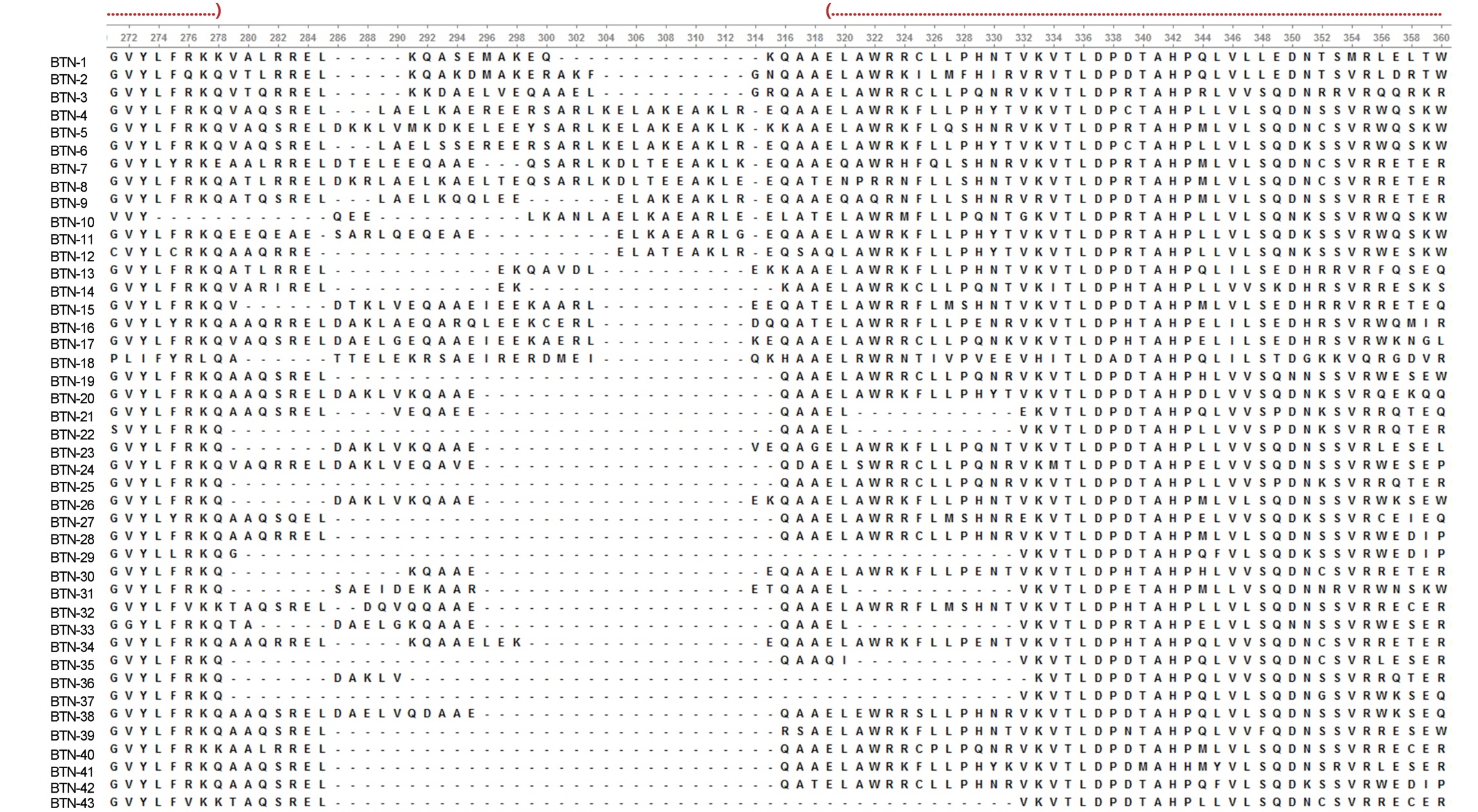


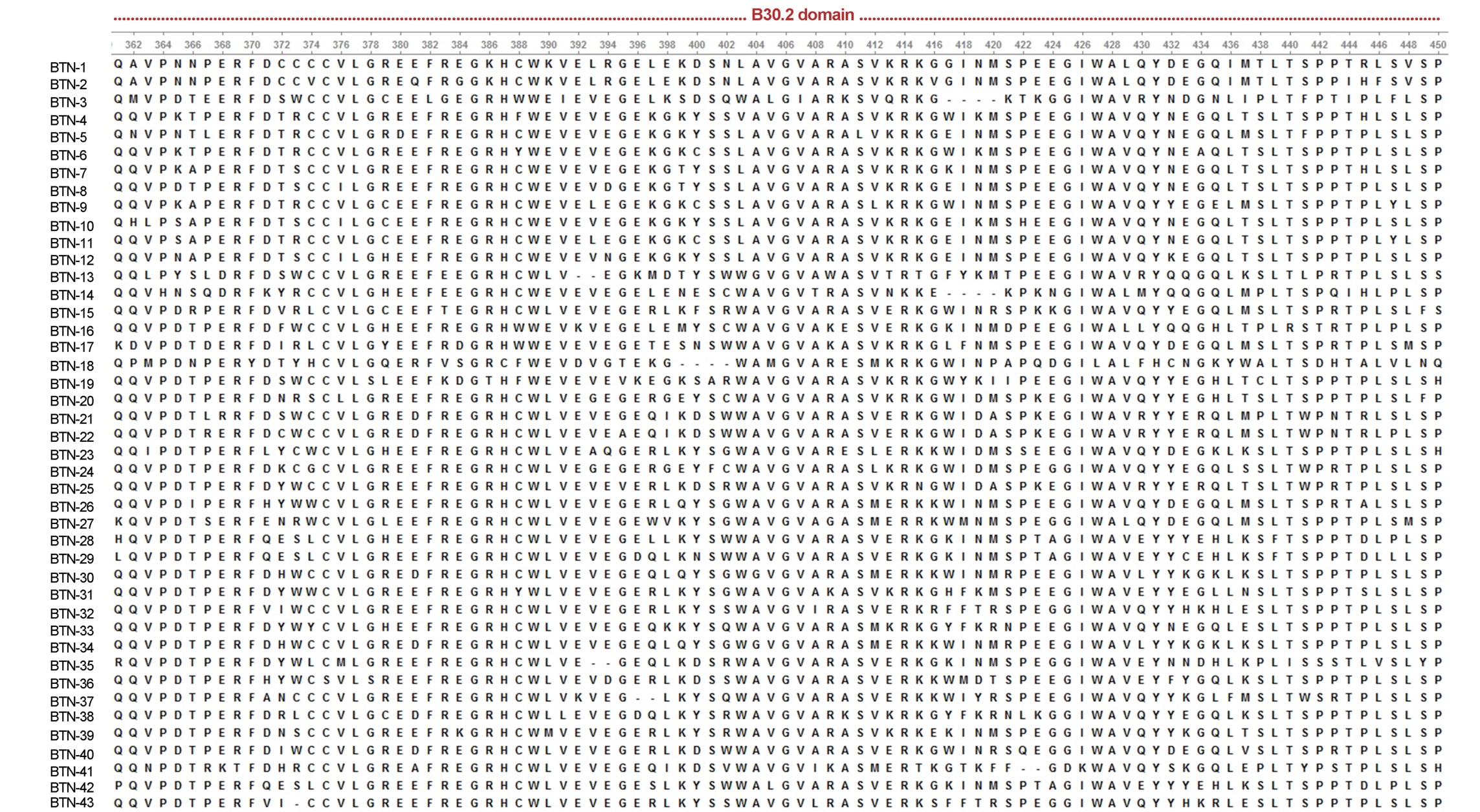


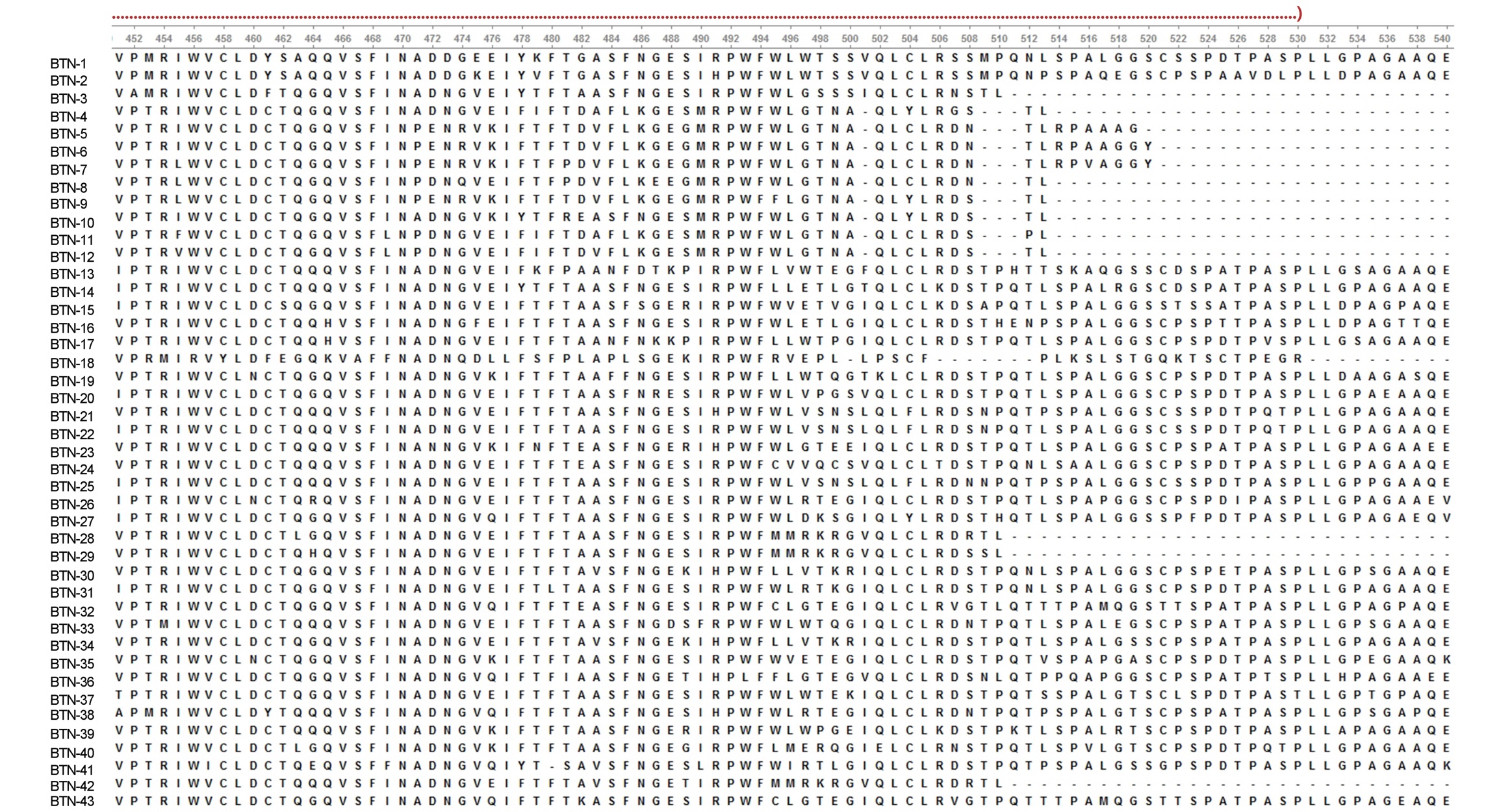


**Fig. S22** Multiple sequence alignment of duck BTN proteins. Duck butyrophilin (BTN) protein sequences were retrieved from our SKLA1.0 genome. Protein structures were predicted using the INTERPROSCAN (http://www.edi.ac.uk/interpro/) with default parameters. Multiple sequence alignments were performed using the Prank software (version 140603) under the “AA” model with 1,000 iterations. “-” denotes gap.


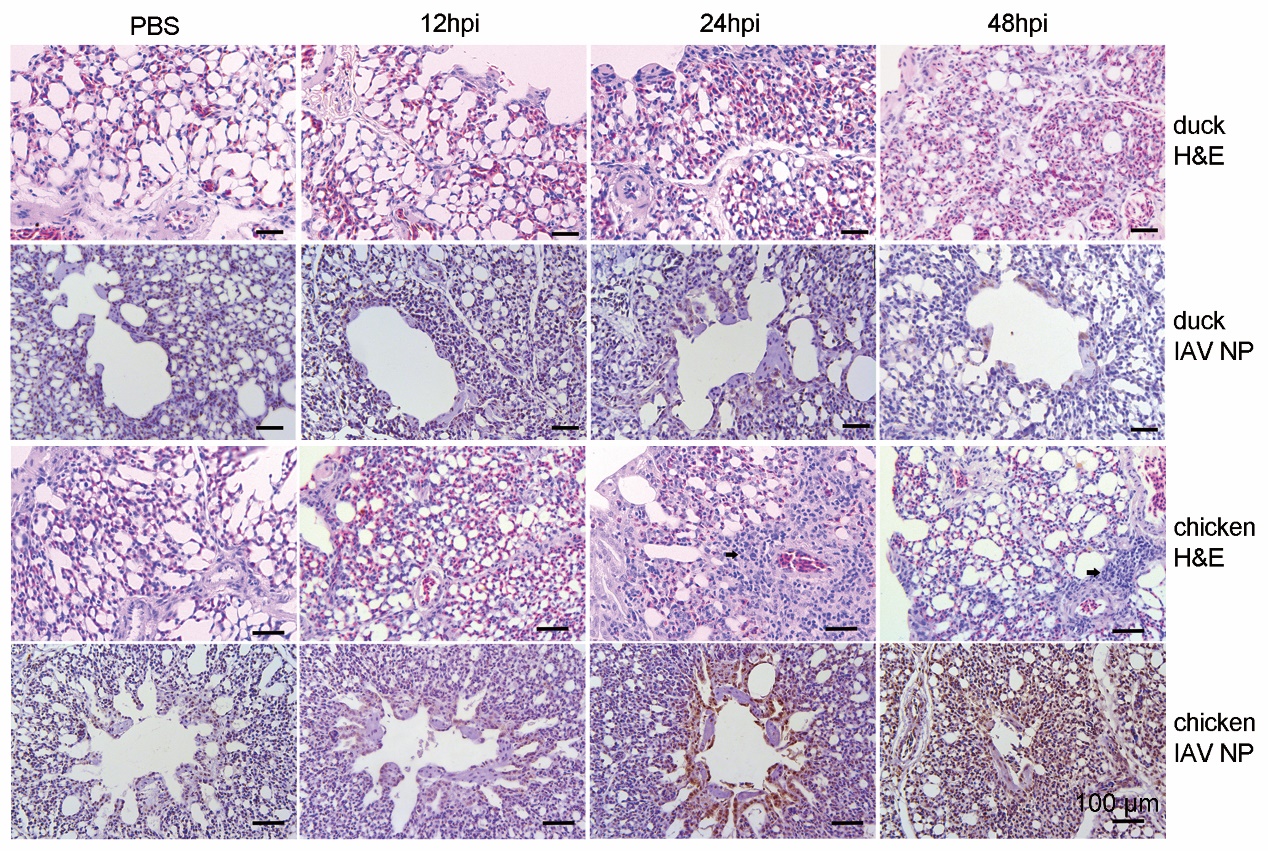


**Fig. S23** Histopathological images of lung tissues from duck and chicken. Animals in experiment and control groups were infected by A/chicken/Sheny/0606/2008 (SY/08) H5N1 virus or PBS respectively. Lung tissues were collected at 12 hours, 24 hours and 48 hours post infection. The section of lung tissue was stained with hematoxylin and eosin (H&E) (Top row for each species). Immunohistochemistry (IHC) for IAV NP gene was used to check for virus-infected cell (Bottom row for each species). X20 magnification, scale bar: 100 μm.


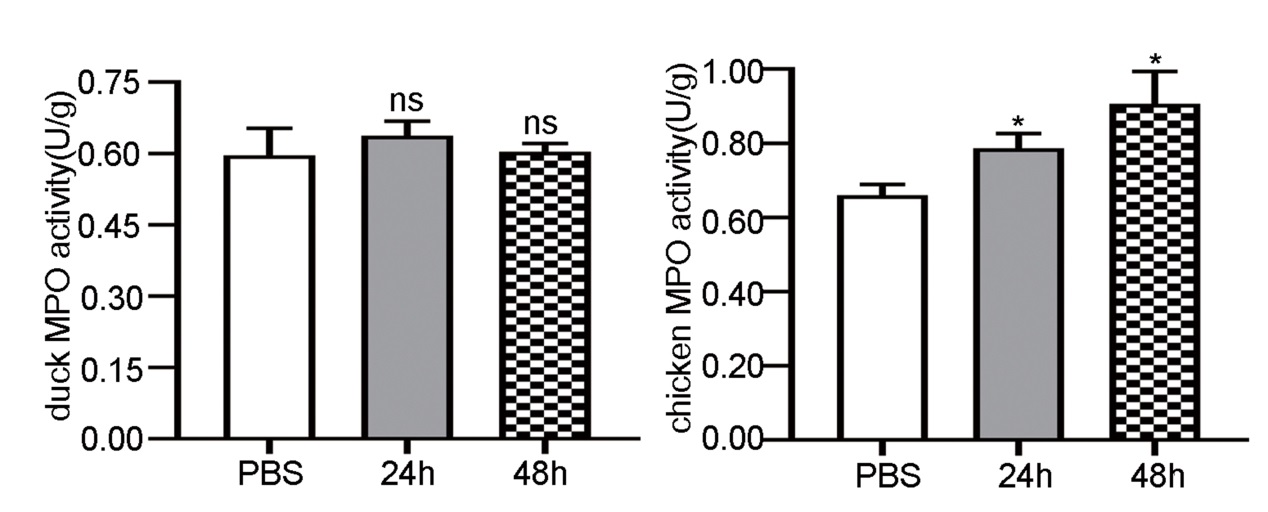


**Fig. S24** Inflammatory cell infiltration in lung tissues of duck and chicken. Animals in experiment and control groups were infected by A/chicken/Sheny/0606/2008 (SY/08) H5N1 virus or PBS respectively. Lung tissues were collected at 24 hours and 48 hours post infection. Lung homogenates were used to analyze level of inflammatory cell infiltration by MPO assay. Data is showed as mean ± SEM (n =5). Significant difference was determined using the student’s t test with threshold of P < 0.05 (*).


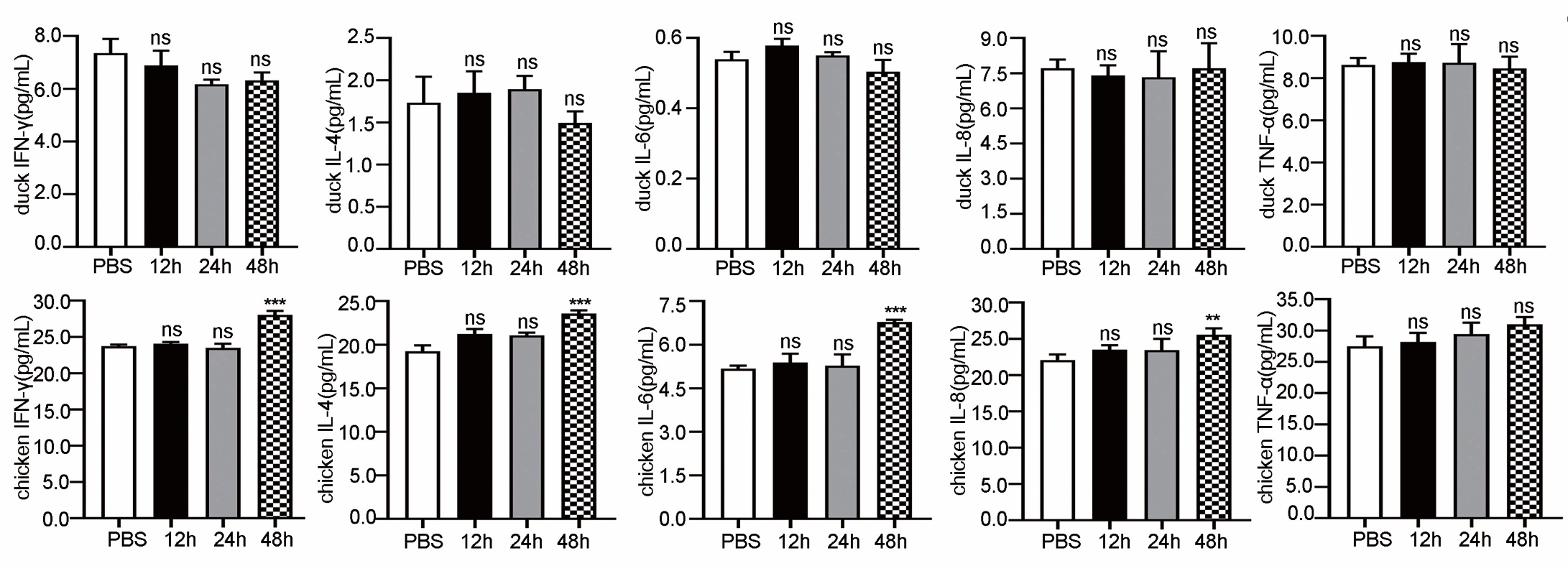


**Fig. S25** Protein level of IFN-γ, IL-4, IL-6, IL-8 and TNF-α in duck (Top) and chicken (Bottom) plasma. Animals in experiment and control groups were infected with A/chicken/Sheny/0606/2008 (SY/08) H5N1 virus or PBS respectively. Protein levels of IFN-γ, IL-4, IL-6, IL-8 and TNF-α in plasma at 12 hours, 24 hours and 48 hours post infection were quantified by ELISA assay. Data is showed as mean ± SEM (n =5). Significant difference was detected using the student’s t test with threshold of P < 0.05 (*).


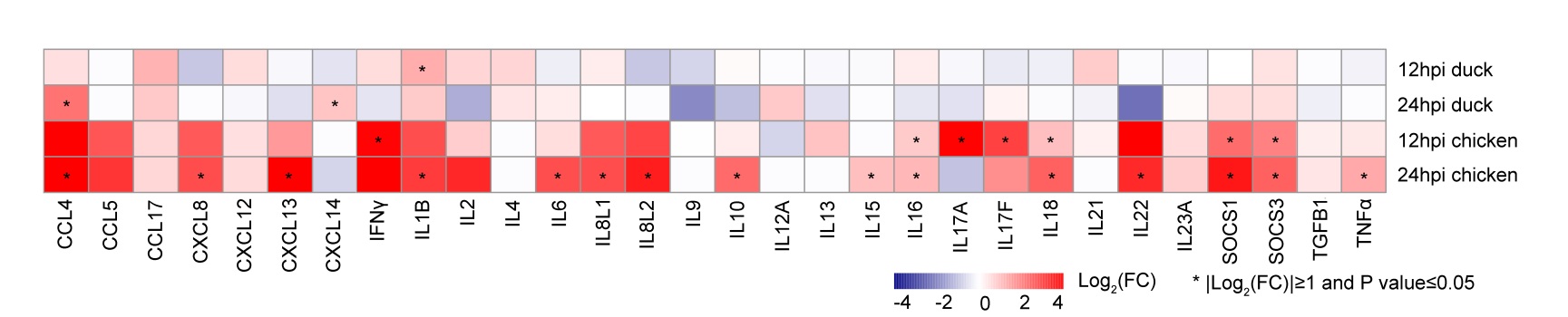


**Fig. S26** Expressional profiles of inflammation-related genes in lung tissues of duck and chicken infected by A/chicken/Sheny/0606/2008 (SY/08) H5N1 virus. Heatmap was generated by using the R packages ggplot2 (http://had.co.nz/ggplot2/) and pheatmap (<https://cran.r-project.org/web/packages/pheatmap/>).


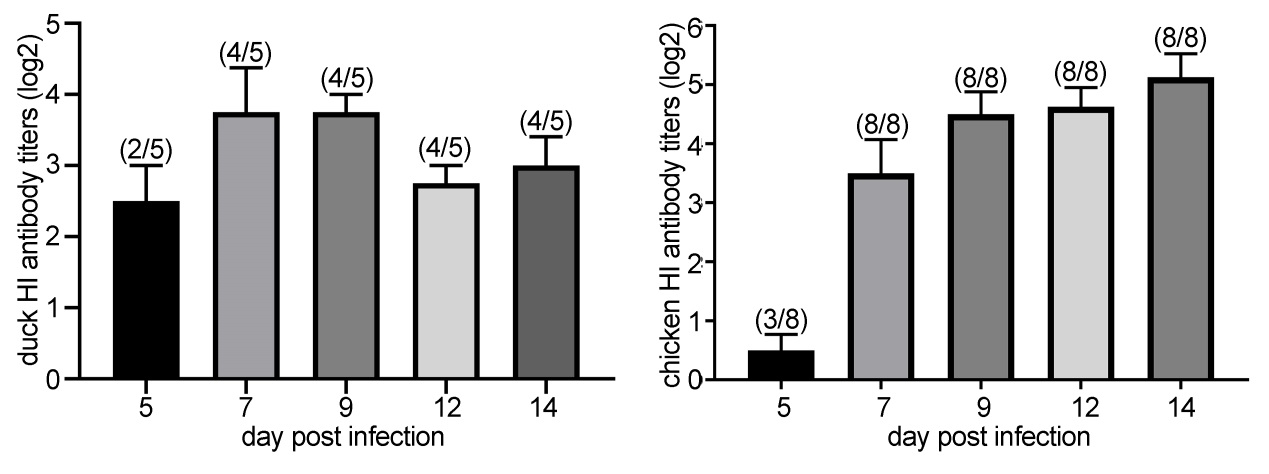


**Fig. S27** Antibody titer in ducks and chickens infected by the recombinant attenuated SY08ΔHA H5N1 virus. Samples were harvested at day 5, 7, 9, 12, and 14 post infection. A haemagglutination inhibition (HI) assay against attenuated SY08ΔHA H5N1 was performed to test antibody levels in duck (left) and chicken (right) serum samples.


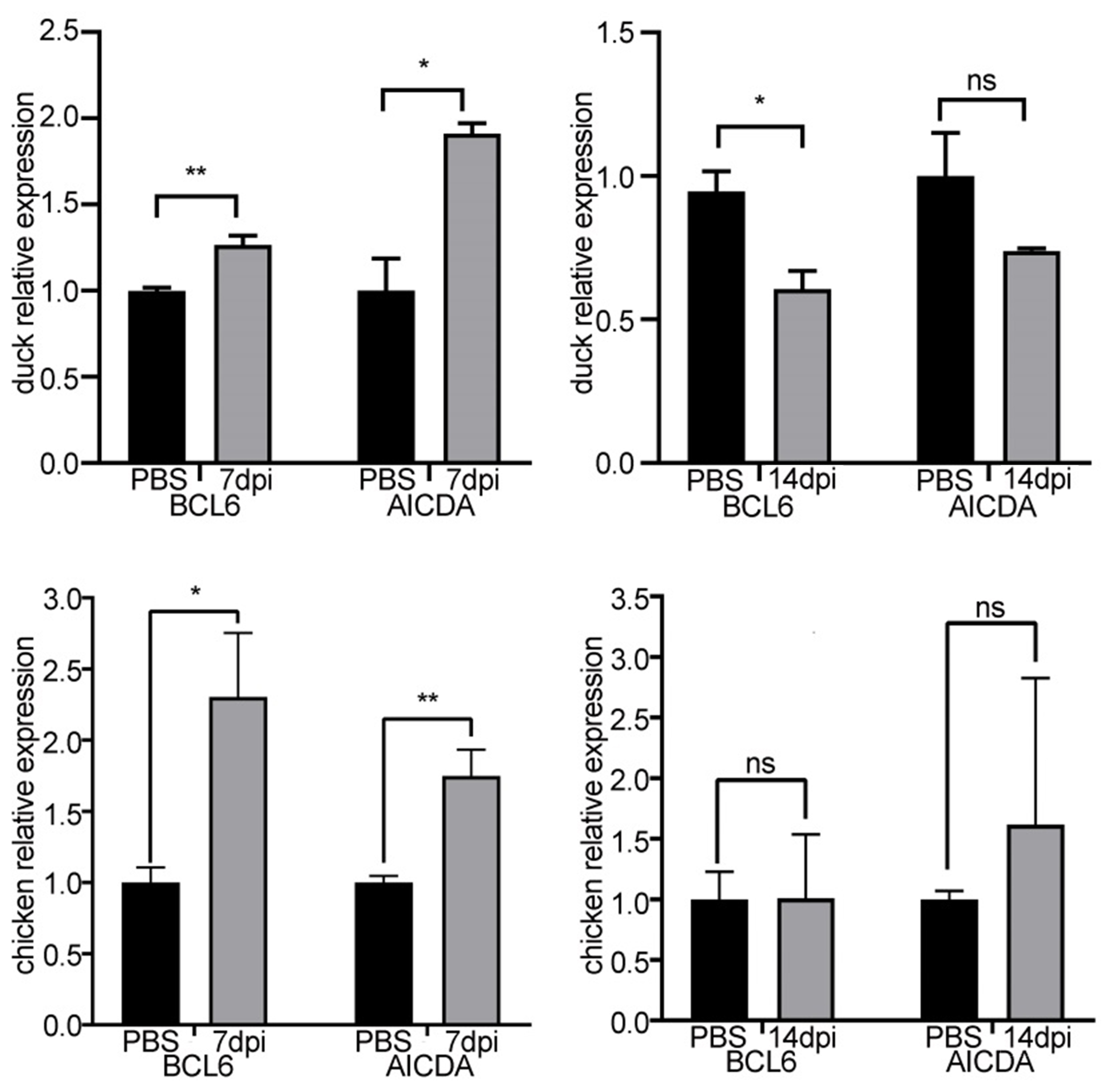


**Fig. S28** Expression of *BCL6* and *AICDA* genes in spleen of ducks and chickens infected by the recombinant attenuated SY08ΔHA H5N1 virus and in control individuals. Samples were harvested at day 7 and 14 post infection. Expression levels of *BCL6* and *ACIDA* were tested by real-time PCR analysis. Data is shown as mean ± SEM (n =3). BCL6 (B cell CLL/Lymphoma 6) is a lineage-defining transcription factor for follicular helper T cells and allows GC B-cells to proliferate very rapidly in response to T-cell dependent antigens. AICDA (activation-induced cytidine deaminase) is a RNA-editing deaminase involving in gene conversion and class-switch recombination of immunoglobulin genes in B cells. We further took these two genes to estimate the activity of CD4+ T and B cell response to the IAV’s infection.


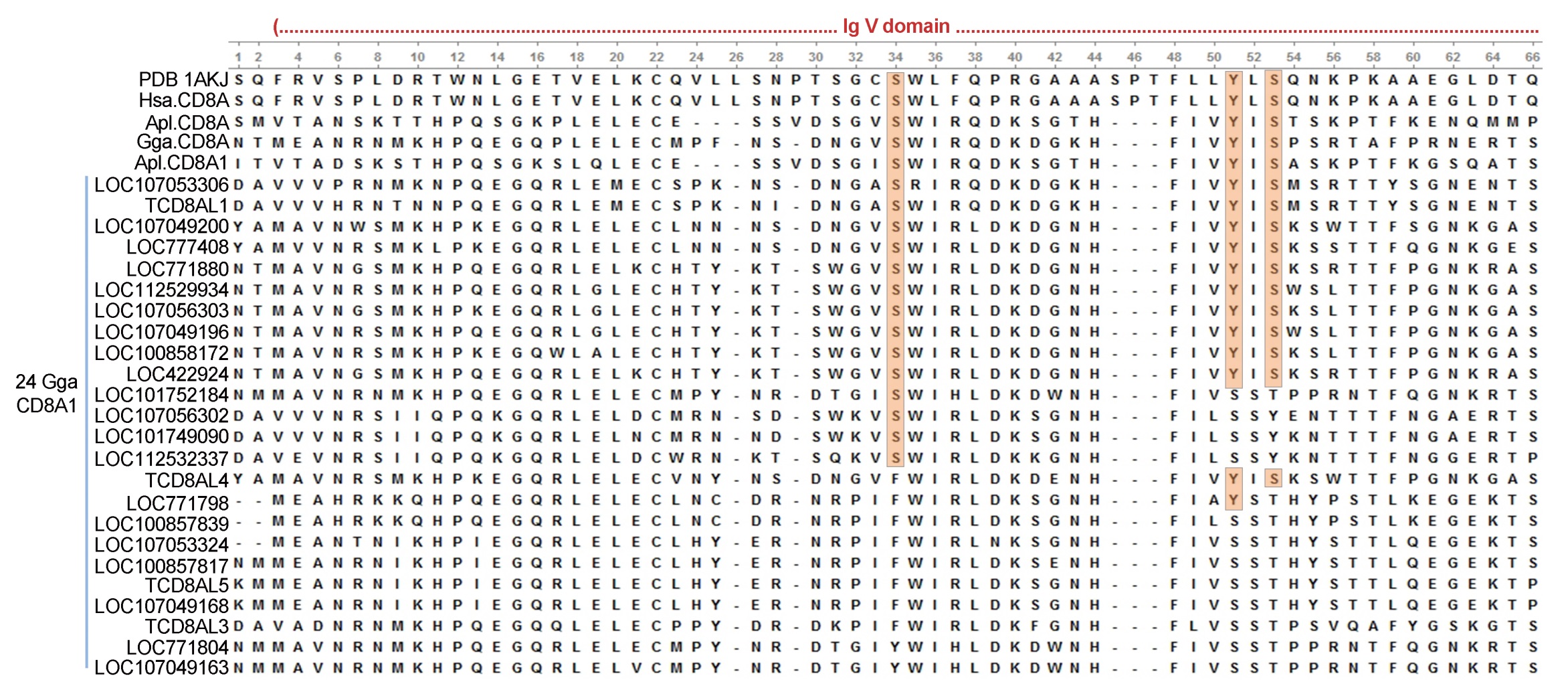


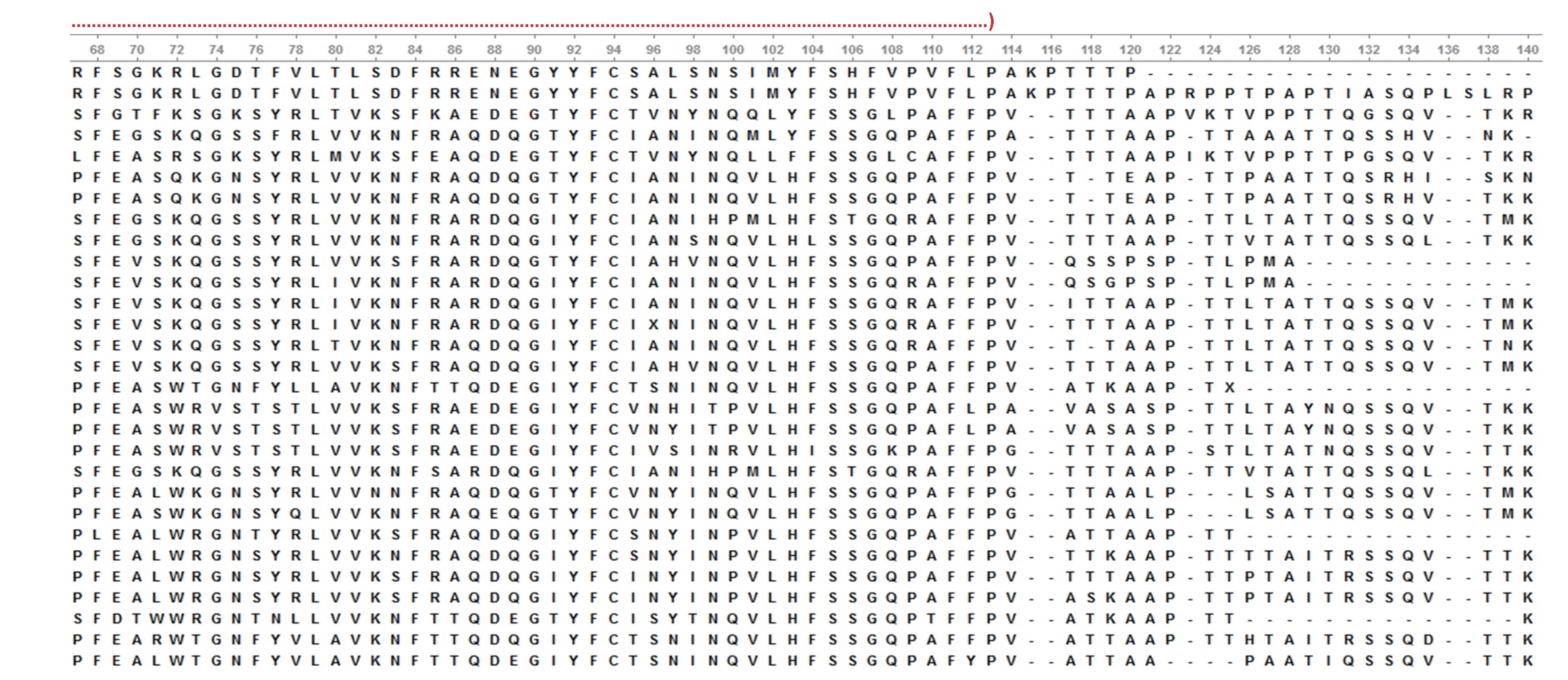


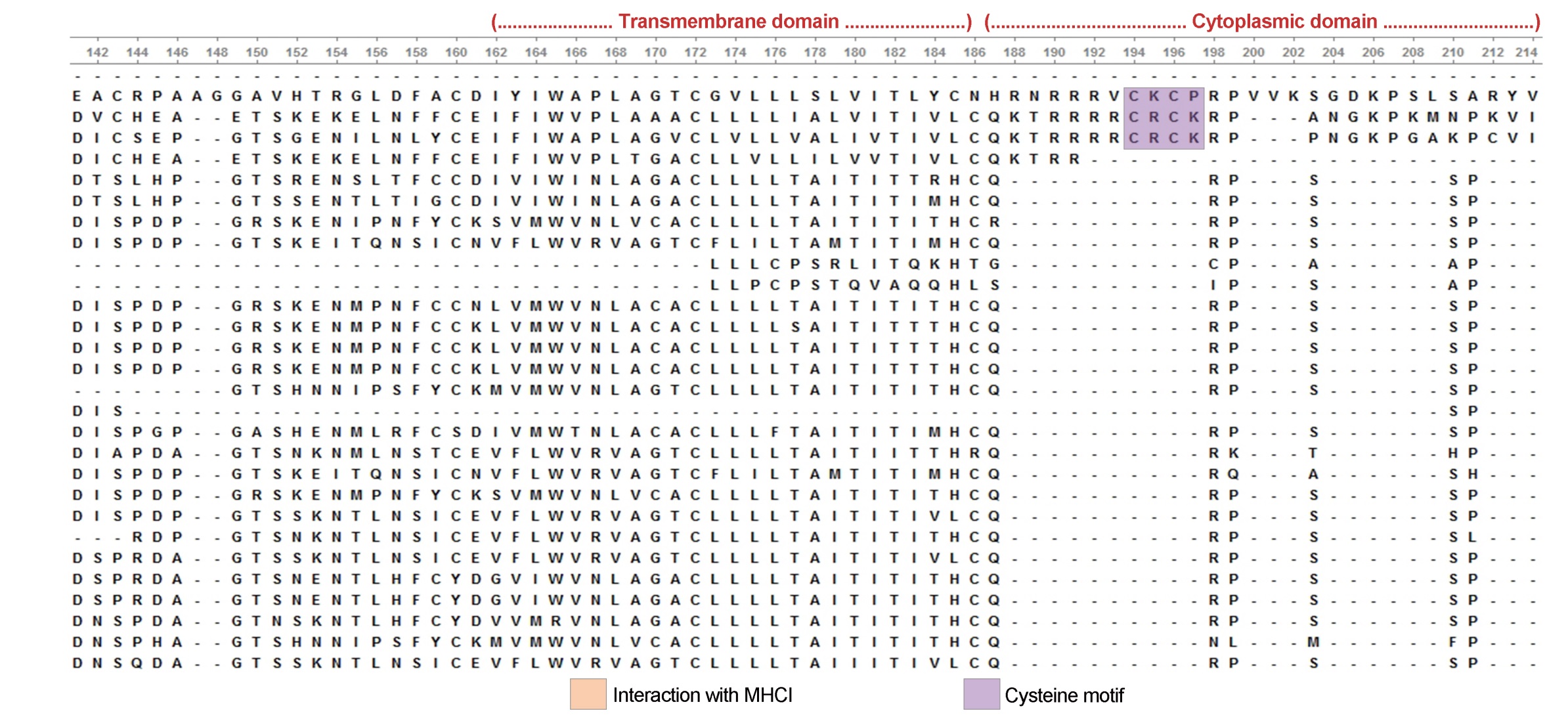


**Fig. S29** Multiple sequence alignment of CD8A proteins. “1AKJ” and “Hsa.CD8A” are reference sequences. Protein sequence domains were predicted using the INTERPROSCAN (<http://www.edi.ac.uk/interpro/>) with defaults parameter.


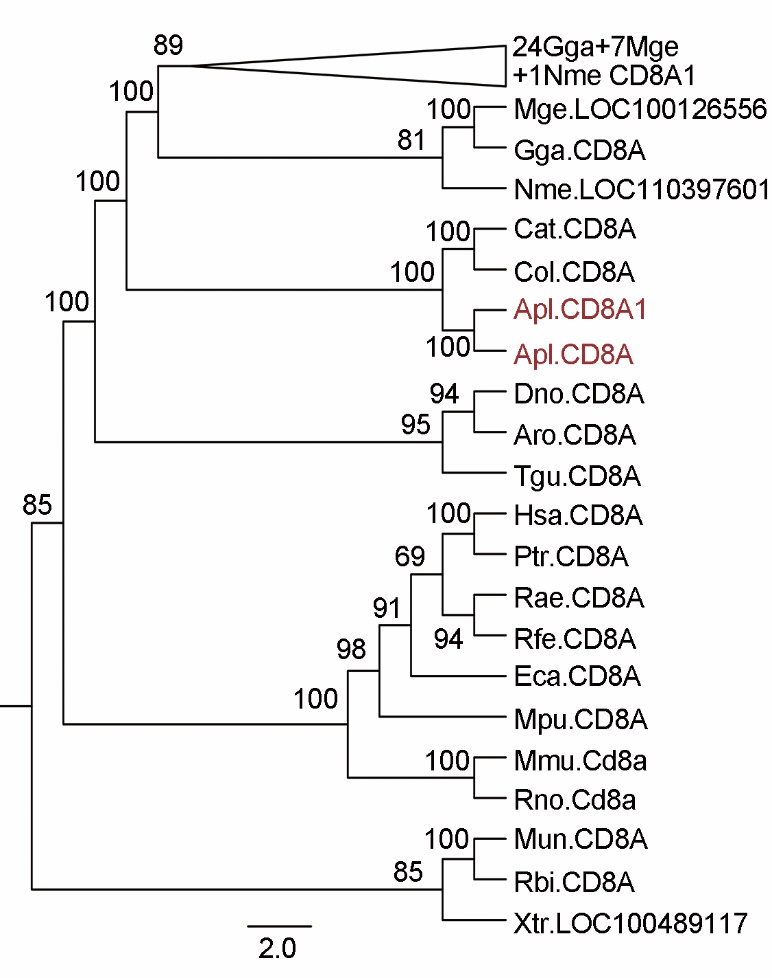


**Fig. S30** Maximum likelihood tree of *CD8As* genes. The tree was generated using 53 CDS sequences from 3 amphibian (tropical clawed frog, Xenopus tropicalis; caecilians, Microcaecilia unicolor; two-lined caecilian, Rhinatrema bivittatum), 8 mammalian (human, Homo sapiens; chimpanzee, Pan troglodytes; Egyptian rousette, Rousettus aegyptiacus; greater horseshoe bat, Rhinolophus ferrumequinum; horse, Equus caballus; domestic ferret, Mustela putorius furo; mouse, Mus musculus; Norway rat, Rattus norvegicus) and 9 birds (duck, Anas platyrhynchos; black swan, Cygnus atratus; mute swan, Cygnus olor; chicken, Gallus gallus; turkey, Meleagris gallopavo; helmeted guineafowl, Numida meleagris; emu, Dromaius novaehollandiae; Okarito brown kiwi, Apteryx rowi; white-throated tinamou, Tinamus guttatus). Apl.CD8A and Apl.CD8A1 were annotated from our SKLA1.0 genome. Other sequences were downloaded from the NCBI website (https://www.ncbi.nlm.nih.gov/). Duck has only 2 CD8A family members while chicken has 25 *CD8A* family members. Multiple sequence alignment was performed using the Prank software (version 140603) under the “DNA” model with 1,000 iterations. The ML tree was built using the IQ-tree software (version 1.6.5). Bootstrap value of 1,000 bootstrap replicates is shown on branches.


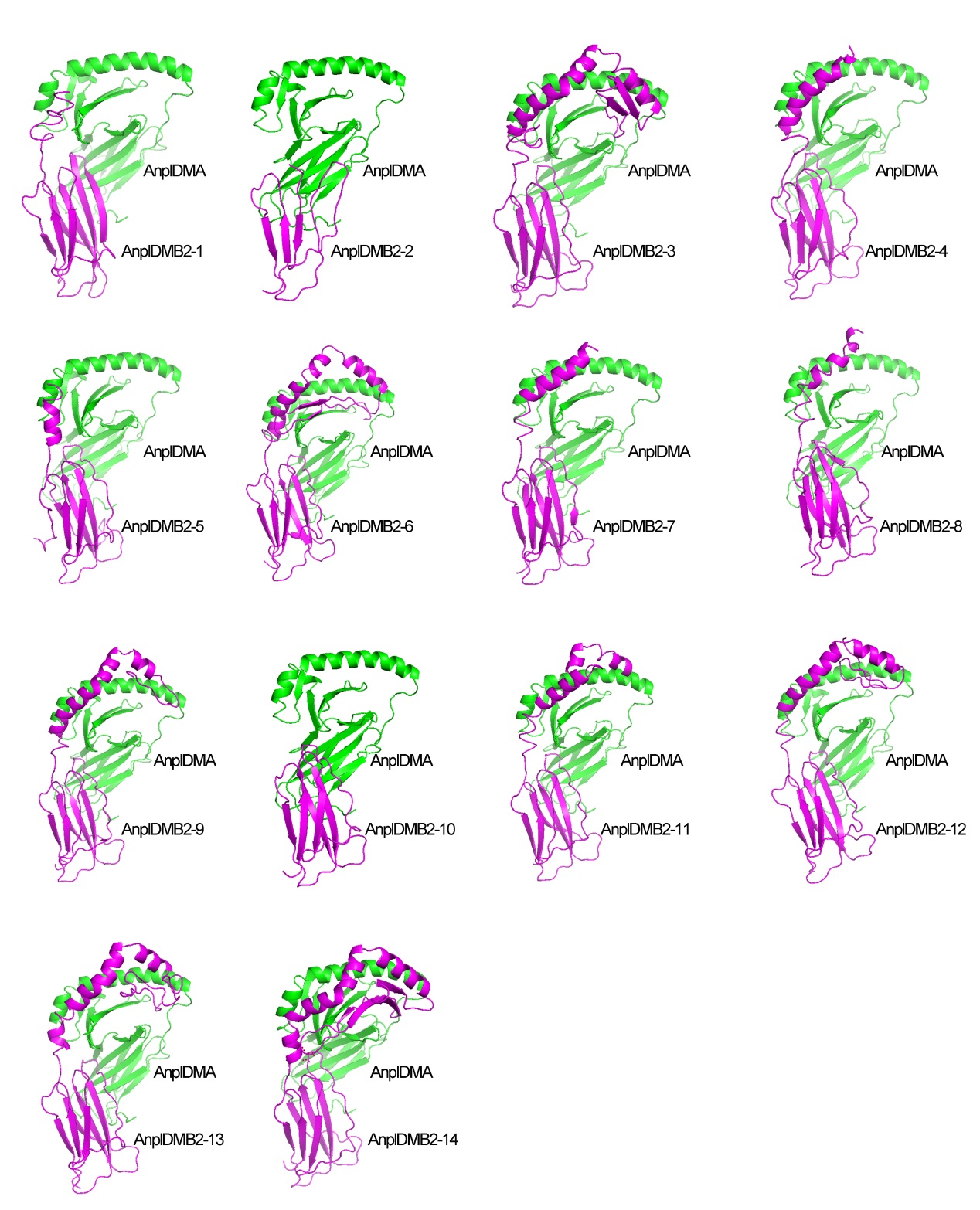


**Fig. S31** Predicted structures of duck DM heterodimers. DMA and DMB proteins mean the α and β chains of the DM heterodimer. Duck has one *DMA* gene and fourteen *DMB2* genes. DMA and DMB proteins were colored in green and purple respectively. Sequence alignment in Additional file 2: Fig.S16 shows that only AnplDMB2-14 has complete β1 and β2 domain, and the other duck *DMB2* genes have lost the β1 domain. AnplDMA and AnplDMB2 protein structures were predicted using 4FQX_C and 4FQX_D as templates. Protein structures were visualized using the PyMOL software (http://www.pymol.org/, version 4.2.0).

**
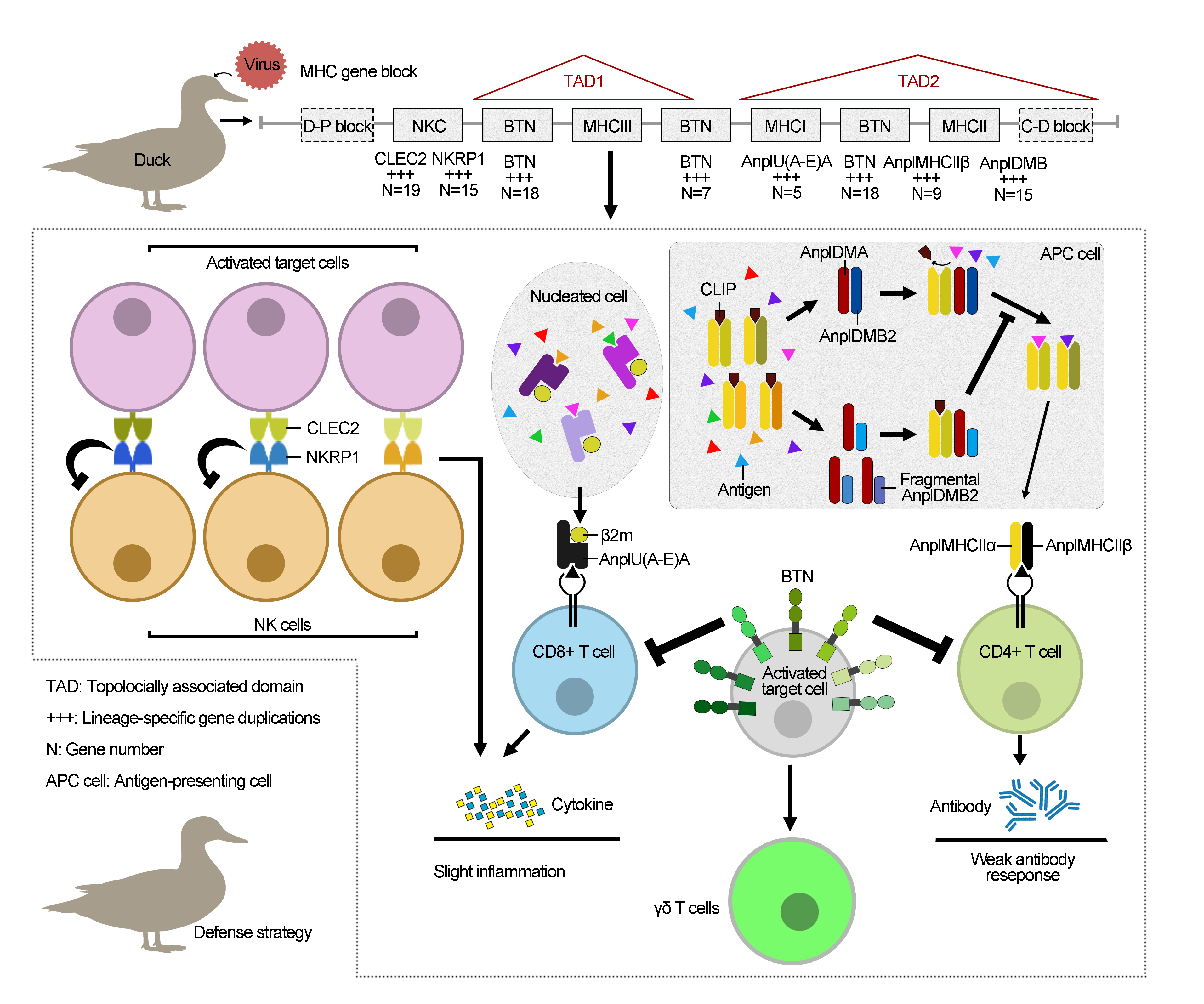
****Fig. 32** A proposed defense model to avian influenza virus in duck.

Duck, as a natural host of IAVs, has evolved a complex MHC model including significantly expanded AnplMHCI, AnplMHCIIβ, AnplDMB, NKRL (NK cell receptor like gene) and BTN genes families. Duck uses divergent AnplMHCI and AnplMHCIIβ genes to recognize pathogens. Moreover, duck can also efficiently regulate its immune response to avoid cytokine storm: where expression of diverse inhibitory NKRP1-like genes and BTNs might reduce secretion of pro-inflammatory cytokines. Moreover, expanded fragmental DMBs might negatively regulate peptide presentation to CD4^+^ T cells. This together with negative regulation of CD4^+^ T cell activation through expanded BTN genes might contribute to weak antibody response. At last, BTNs may also activate γδ T cells to eradicate virus.


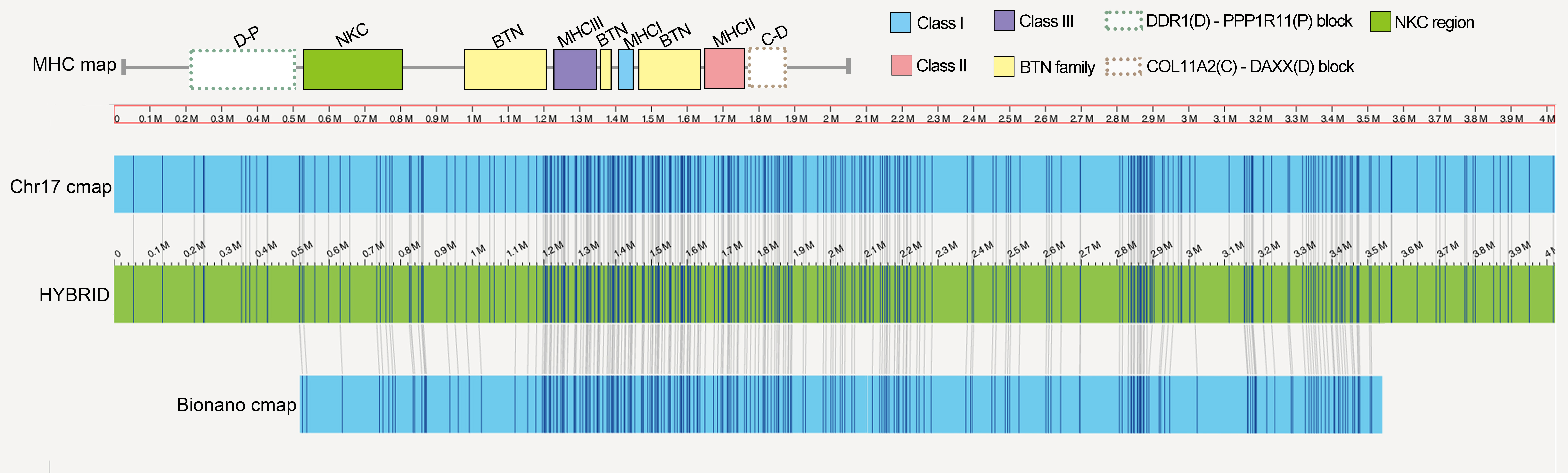
 **Fig. S33** Comparison of chromosome 17 (chr17) cmap and Bionano cmap. Chr17 cmap was generated by *in silico* digestion using the SOLVE toolkit according to chr17 genomic sequence (https://bionanogenomics.com/support/software-downloads/, version 3.2.1). Bionano cmap was generated by *de novo* assembly with the BNX raw reads. HYBRID scaffold was generated by comparing chr17 cmap and bionano cmap using SOLVE software. Chr17 contained only one contig, with the MHC region located at 0.04 Mb to 1.86 Mb (the specific gene information refers to Fig. 2a and Supplementary Data 1).


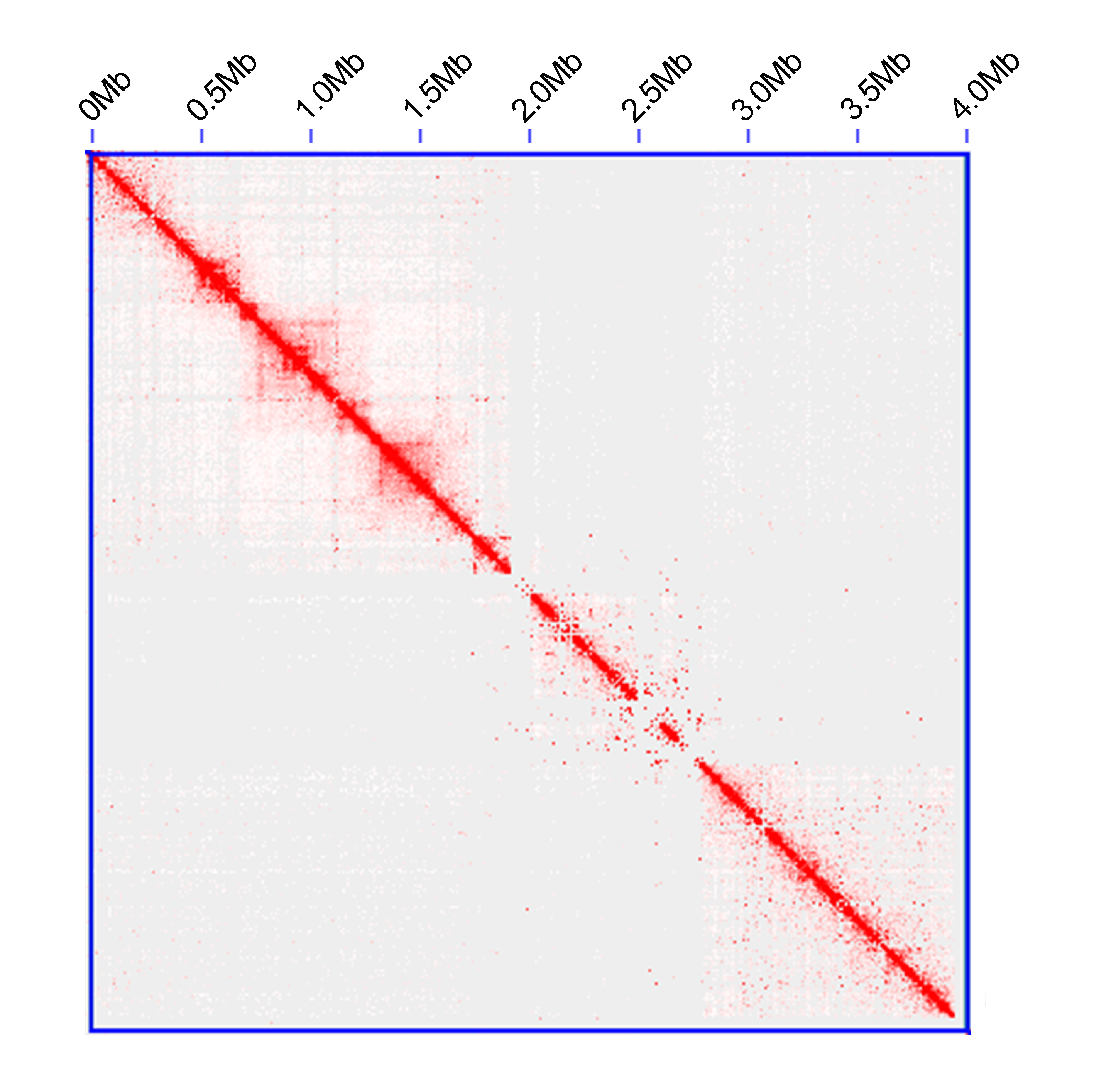


**Fig. S34** Hi-C matrix map of chromosome 17. The contact matrix map was generated by aligning and refining Hi-C data from a duck (C18) to its genome assembly (SKLA1.0) using juicer (version 1.5) and 3d-dna (version 180922) software with default parameters. Matrix visualization was performed using juicebox software (version 1.13.0). Position is marked on the top of the map. The map shows that chromosome 17 has good assembly quality.
