# Supplementary material for: A new chromosome-scale duck genome shows a major histocompatibility complex with several expanded multigene families
Source: BMC Biol. 2024 Feb 5;22:31. doi: 10.1186/s12915-024-01817-0 (PMC10845735; doi:10.1186/s12915-024-01817-0)
Supplement: Supplementary file 6 — Additional file 6: Detailed materials and methods. [file 12915_2024_1817_MOESM6_ESM.docx]

**Supplementary Note**

**Data generation**

**Source organism**. DNA was extracted from the blood of a male Pekin duck (Anas platyrhynchos) in a Poultry Institute of Jiangsu, China. This is a 14-week old male Beijing duck named C18. C18 duck is well developed and in good health. This duck is a hybrid offspring of native male and female Pekin ducks (Gold star Duck Production, Beijing, China).

**Nanopore sequencing**. High-molecular-weight DNA was extracted from fresh blood and muscle of C18 using the QIAGEN Genomic DNA kit (Qiagen, Hilden, Germany). Seven 1D libraries with an insert fragment than 20 Kb were constructed and sequenced on Oxford Nanopore Technology GridION X5 instrument at the Genome Center of NextOmics Bioscience (Wuhan, China). This effort yielded 82.56 Gb clean reads (Additional file 1: Table S2). Moreover, two ultra-long libraries were prepared and sequenced on the PromethION instrument. One library generated 9.68 Gb ultra-long clean reads and the other one generated 18.26 Gb clean reads (Additional file 1: Table S2).

**Bionano data**. High-quality genomic DNA was isolated from fresh blood of C18 and two libraries were constructed according to protocols offered via the Bionano genomics official website (https://bionanogenomics.com/). DNA was degenerated into fragments using DLE-1 enzyme with a label density of 8~25/100kb. DNA was loaded onto to Saphyr instrument to produce optical molecules, with molecules less than 150 Kb filtered out. A total of 259.60 Gb clean data were generated with 18.17 labels per 100 Kb and the N50 length was 274.90 Kb (Additional file 1: Table S5).

**Hi-C data**.  Liver tissue of a 14-week male duck (C18) were cut into 2-3 mm blocks and about 1mL blocks were fixed with 2% formaldehyde in DMEM (Gibco) at room temperature for 10min. Cross-linking reaction was quenched by adding a final concentration of 0.125M glycine solution. Blocks were incubated at room temperature for 5min and subsequently at 4℃ for 15min. Sample was then centrifuged for 10min at 4℃, removed the supernatant and lysed using ice-cold Hi-C lysis buffer. The nuclei were permeabilized and the chromatin were digested with HindIII enzyme at 37℃. Restricted fragments were labeled using biotin-14-ATP. After reversal of crossing-links, DNA were purified, sheared to a length of ~400bp, and pulled down using streptavidin beads in binding buffer. DNA was then quantified and sequenced on an Illumina Hi-Seq X Ten platform with 150bp paired-end read lengths. This effort produced 274.19 Gb PE150 paired-end clean reads (Additional file 1: Table S7).

**Short-read genomic DNA data**. DNA was extracted from fresh blood of C18 using the QIAGEN Genomic DNA kit (Qiagen, Hilden, Germany) according to the protocol offered by the Genome Center of NextOmics Bioscience (Wuhan, China). A library with 400 bp insert size was constructed and sequenced on the Illumina Hiseq X ten platform. A total of 136.81 Gb PE150 paired-end clean reads with 117-fold coverage of the duck genome were generated.

**RNA-seq data.** Total RNA was extracted from lung tissues using Trizol (Invitrogen, USA) according to the manufacturer instructions. RNA sample concentration, RIN, 28S/18S and size were detected using the Agilent 2100 Bioanalyzer (Agilent RNA 6000 Nano Kit) and samples with RIN ≥ 8.50 were used to construct cDNA library according to the Illumina protocols. cDNA libraries were assessed using the Agilent 2100 Bioanalyzer and sequenced to generate 150-bp pair-end reads using the Hi-Seq 4000 System (Tru-Seq SBS KIT-HS V3, Illumina).

**Genome assembly**

Before assembly, we first estimated the genome size and heterozygosity of C18 using short Illumina reads by K-mer analysis. Iteratively selecting a sequence of K bases from a continuous sequence, if the read length is L and the length of the K-mer is K, then L-K+1 K-mers can be obtained. We used K=17 for the subsequent analysis. K-mers from the Illumina data were counted using Jellyfish, and the frequency of each K-mer determined. Taking the frequency (can also be called K-mer depth) as the abscissa, and the percentage of total K-mer segments in which the frequency appears as the ordinate, and a frequency distribution map of all k-mers was obtained. Without regard to the sequencing error rate, the heterozygosity of the genome, or the degree of repetition, the K-mer curve is subject to a Poisson distribution in an ideal state. According to the Lander Waterman algorithm, genome size (G) is related by the following formulas: (K_num_ is the number of K-mer, K_depth_ is the expected depth of K-mer, b_num_ is the number of bases, and b_depth_ is the expected depth of the base)

$G=\frac{K_{num}}{K_{depth}}$=$\frac{b_{num}}{b_{depth}}$

The 17-mer distribution curve is an abnormal Poisson distribution, showing a bimodal distribution, and with a peak value near 54x and 109x respectively. The main peak was 109. K_depth_ was 109, and K_num_ was 127,690,067,177. According to the formula, the genome size is therefore 1.17G. To predict the genome heterozygosity, the Arabidopsis genome was used to simulate the short-segment data of the corresponding depth, and the K-mer curve fitting was performed under the condition of different gradients of the heterozygosity. Ultimately, the genome heterozygosity was 0.58 %.

Raw normal and Ultra-long Nanopore reads were filtered using thresholds of read length ≥ 5 kb and q value ≤ 7. Clean Nanopore reads were assembled using the NextDenovo software (<https://github.com/Nextomics/NextDenovo>, version [2.1-beta.0](https://github.com/Nextomics/NextDenovo/releases/tag/v2.1-beta.0)). Other parameters were set according to our DELL server: sort_options=-m 40g -t 10, minimap2_options_raw=-t 10, pa_correction=5, correction_options=-p 20, minimap2_options_cns = -t 20, nextgraph_options = -a 1. Contigs were then polished three rounds with Illumina short reads using the Nextpolish software (version 1.2.3) under the following parameters: task=best, rerun=3, parallel_jobs=6, multithread_jobs=5, genome_size=1.2g, sgs_fofn=./sgs.fofn, sgs_options=-max_depth 100 -bwa, lgs_fofn=./lgs.fofn, lgs_options=-min_read_len 1k -max_depth 100, lgs_minimap2_options=-x map-ont. After that, high quality contigs and Bionano optical maps were used to generate hybrid scaffolds using seven R packages (data.table, igraph, intervals, MASS, parallel, XML and argparser), and short and low quality contigs were removed using the RefAligner tool in the SOLVE software (https://bionanogenomics.com/support/software-downloads/, version 3.2.1). Genome cmap file was generated using the perl fa2cmap_multi_color.pl script. *De novo* assembly of the bionano cmap file was conducted using the CL.py script under the following parameters: -T 80 -j 80 -N 4 -f 0.2 -R -y -I 7 -a optArguments_nonhaplotype_noES_noCut_DLE1_saphyr.xml. Hybrid scaffolds were constructed using the hybridScaffold.pl script.

Hi-C reads were filtered using Trimmomatic software (http://www.usadellab.org /cms/index.php?page=trimmomatic, Version 0.36) with threshold of Q value < 20. Scaffolds were indexed and *in silico* cut using the BWA software and the generate_site_positions.py script respectively. After that, we mapped Hi-C data to the above scaffolds using the juicer (version 1.5) software and assigned these scaffolds into chromosomes with the 3d-DNA (version 180922) software. After Polish, Split, Seal and Merge steps, we obtained a raw genome draft. Hi-C data visualization and interactive assembly refinement was performed manually using the Juicebox (https://github.com/aidenlab/Juicebox, version 1.13.01) software. We further performed three rounds of gap filling using the Gapcloser (https://github.com/ BGI-Qingdao/TGS-GapCloser, version 0.56) software with normal and Ultra-long Nanopore reads with default parameters to gain the final genome assembly.

**Gene annotation**

We prepared four data sets for genome annotation: data set1, we collected a total of 456.94 Gb pair-end RNA-seq reads from 83 samples (Additional file 1: Table S9). These data were download from NCBI SRA website (https://www.ncbi.nlm. nih.gov/sra/) or obtained from our unpublished works; data set2, we assembled data set1 with the Trinity-v2.8.5 software under the default parameters; data set3, we prepared 307,554 full-length transcripts generated by the Pacbio Sequel platform. This included 199,993 transcripts from the NCBI Transcriptome Shotgun Assembly database (accession number GHJL00000000.1) and 107,561 transcripts were obtained from our unpublished work; data set 4, we collected 77,519 protein sequences from duck (BGI_duck_1.0), chicken (GRCg6a), mouse (GRCm39) and human (GRCh38.p13) for homology-based annotation. For gene encoded multiple protein isoforms, we retained the longest protein sequence.

Gene prediction was performed using the GETA pipeline (https://github.com/ chenlianfu/geta, version 2.4.4) with the data set 1 and 4 under the following parameters: --RM_species chicken --augustus_species chicken_geta --pfam_db Pfam-AB.hmm. We also predicted genes using the Funannotate pipeline (https://github.com/next genusfs/funannotate, version 1.7.4) with data set 2, 3 and 4 under the following parameters: –s "chicken" --busco_db aves --max_intronlen 10000 –organism other. After that, we integrated gene set generated by the GETA and Funannotate pipeline, and filtered the combined gene sets by searching against homologous proteins with e-value < 1e-10. We further removed redundant genes using a R script and obtained a reference set including 17,896 coding genes. Finally, we added transcripts information through a three-round update with data set 2 and 3 using the PASA software.

**Evaluation of contiguity and completeness of the genome assembly and annotated gene set**

To assess quality of the duck assembly, we first compared the SKLA1.0 assembly to another four genome drafts (BGI1.0, ZJU1.0, GRCg6a, and bTaeGut1.4.pri) for contiguity and completeness (Table 1). We also performed TreeMap to scale the length of contigs using the R package TreeMap (https://cran.r-project.org/web/ packages/treemap/index.html) (Fig. 1a). We then compared mapping ratio of 83 Illumina PE90/PE150 RNA-seq datasets from nine tissues on three duck assemblies (BGI1.0, ZJU1.0, and SKLA1.0) (Additional file 1: S9). RNA-seq reads were mapped to the three duck assemblies using Hisat2 (version 2.1.0) software with defaults (Fig. 1b). We then aligned 1,625,932 transcripts (data2 and data3) to the three duck assemblies using the GMAP (version 2018-07-04) with defaults (Fig. 1c).

We compared number of coding genes and transcripts of SKLA1.0 with the other four genome drafts (BGI1.0, ZJU1.0, GRCg6a, and bTaeGut1.4.pri) (Fig. 1e). We evaluated completeness of the assembled genome and coding genes using the BUSCO software (version 3.0.2) with 8,338 BUSCO avian proteins from the OrthoDB database (version 10) (Fig. 1d).

We counted gaps upstream and downstream of each gene (Fig. 1f). For example, at the 1kb level, we extend 1 kb sequence upstream and downstream of each gene. The percentage of gaps was the proportion of genes with gaps in its upstream or downstream regions compare to the total number of genes.

**Gene expansion and contraction analysis**

Protein sequences of seven species (chicken, zebra finch, emu, Egyptian rousette, greater horseshoe bat, human, and tropical clawed frog) were retrieved from NCBI and duck protein sequences annotated in this study were grouped using the OrthoMCL pipeline. Gene group ID was collected by uploading human and chicken protein ID to the PANTHER database (http://www.pantherdb.org/) and groups with the same PATHER family ID were combined. This effort resulted in a total of 6,606 gene families including 1,800 single-copy gene families and 4,806 multiple-cope gene families. We then performed multiple sequence alignments using the Prank (version 14063), trimmed all aligned sequences using the Gblock (version 0.91b) with default parameters and concatenated the aligned and trimmed single-copy proteins of each species into one. The super-proteins were used to generate a maximum likelihood species tree using the IQ-tree software (version 1.6.5) under a JTT + G substitution model with 1,000 bootstrap replicates. Species divergent time was referenced from the Timetree website (http://www.timetree.org/). The orthologous gene family and the phylogenetic tree were used to detect gene gain and loss with the CAFÉ (version 4.2.1) software. This effort showed that 498 out of 4,806 multiple-copy gene families were significantly expanded or contracted (P-value ≤ 0.05). Among them, 38 significantly expanded or contracted gene families were seen to be listed in InnateDB, an Innate Immunity Genes database (https://www.innatedb.com/), and thus defined as immune-related gene families.

**Multiple sequence alignment and maximum likelihood tree construction**

Multiple sequence alignment was performed using the Prank (version 14063) with 1,000 iterations under the "DNA" model for CDS sequence and the "AA" model for amino acid sequences. The Maximum-likelihood tree was inferred using the IQ-TREE software (version 1.6.5) with a bootstrap replication of 1,000 after selecting the best model according to the Bayesian information criterion (BIC) score reported in model test. Tree was viewed using the Figtree software (http://tree.bio.ed.ac.uk/software/figtree/, version 1.42,). There are many phylogenetic trees in the Manuscript and Additional files. We have unified the branch names in these phylogenetic trees. The standard is as follow: “species name”+“.”+“gene name”. The “species name” has been abbreviated, refer to Additional file 1: Table S20. The “gene name” is the gene symbol of the NCBI database in each species (duck gene names are from our data).

**Structure prediction, pocket volume calculating and molecular docking**

Protein sequence was searched using the SWISS-MODEL website (https://www.swissmodel.expasy.org/). Point mutation was made and optimized using the Discovery Studio 2019, and protein structure was predicted with the I-TASSER software (https://zhanglab.ccmb.med.umich.edu/I-TASSER/) and viewed using the PyMOL software (http://[www.pymol.org/](http://www.pymol.org/), version 4.2.0). Structural similarity between predicted protein structures and template tertiary structure was measured by counting the global small root mean square deviation (RMSD) using the PyMOL program (http://www.pymol.org/, version 4.2.0). The global model quality estimation (GMQE) score was counted using the SWISS-MODEL program with defaults (http://www.swissmodel.expasy.org/). A/chicken/Sheny/0606/2008 (SY/08) H5N1 virus HA protein sequence was cleaved into small peptides by a sliding window according to the motif length reported in the literature and docked to structure of MHCI using GalaxyPepDock (https://galaxy.seoklab.org/cgi-bin/submit.cgi?type= PEPDOCK). Dock models were viewed using the Pymol software and filtered according to interaction between B and F pockets of MHCI and peptide. Electrostatic potential (EP) and lipophilic potential (LP) maps were estimated using the MOLCAD program in the SYBYL (version X2.1.1) software.

**Information of samples used in RNA-seq analysis**

Beijing duck and Shaoxing duck are two common breeds used in researches about avian influenza viruses. 12 Beijing ducks were divided into two groups, namely the control group and the treatment group randomly (6 VS 6). Ducks in control group were infected with PBS and ducks in treatment group were infected with H5N1 SY/08 virus. Lung samples were harvested in 12 hours and 24 hours post infection (Additional file 3, Data S6). Besides ducks, the same experiments were also carried out using chickens. In addition, to understand the expression of MHC genes in other tissues, we used unpublished duck liver transcriptome data from our laboratory (Ning et al) as well as RNA-seq data download from the NCBI SRA (https://www.ncbi.nlm.nih.gov/sra/?term=) database (Additional file 3, Data S6). The DK/49 and GS/65 H5N1 viruses were isolated from a duck and a goose, respectively. These data sets contain 8 samples, namely, breast muscle, ileum, jejunum, duodenum, lung, spleen, liver and brain.

**Generation of a recombinant attenuated H5N1 virus and antibody test**

Since the SY/08 H5N1 virus causes the death of chickens, we constructed a recombinant attenuated SY08ΔHA H5N1 virus using reverse genetics as described previously, which expressed a mutated HA protein containing an amino acid deletion (G325) in the HA cleavage site (HACS) region and leaving the seven other virus proteins. The SY08ΔHA virus was verified by the Sanger sequence method, propagated in 10-day specific pathogen-free (SPF) chicken embryos and stored at -80℃. Viral titer was determined with EID_50_ individuals using the Reed and Muench method.

Two groups of 67-day SPF Shaoxing ducks were inoculated intranasally with 600 uL of 10^8.5^EID_50_ of SY08ΔHA H5N1 virus or PBS by dripping into the trachea. Serum samples collected from 10 individuals of each group after days 3, 5, 7, 9, 12 and 14 post inoculation were tested for antibody titer using hemagglutination inhibition (HI) assays. HI assays were performed in accordance with the manual accompanying the annually distributed WHO kits. Virus neutralization tests were performed in a V-bottomed plate. Serum samples diluted at 1:2, 1:4, 1:8, 1:16, 1:32, 1:64, 1:128 and 1:256 were added in equal volume (25 μL) with four hemagglutination units (HAU) of virus and incubated at 20℃ for 30 minutes. After incubation, 25 μl of 1% duck or chicken erythrocyte suspension was added and incubated at 20℃ for 15 minutes. We defined the hemagglutination titer under thresholds of the well with the largest diluted proportion showing signs of visible hemagglutination. Besides ducks, the same experiment was also carried out using chickens.

Spleen tissues of 10 duck from each group were collected at day 7 and 14 post inoculation, and total RNA was extracted using Trizol (Invitrogen, Rockvile, MD, USA). cDNA was generated using the High-capacity cDNA Reverse Transcription Kit (Invitrogen, Rockvile, MD, USA). Gene expression was quantified using the 2^-ΔΔCt^ method by the quantitative PCR with primers in Additional file 1: Table S22 and normalized to the corresponding internal reference gene ─ *GAPDH*. Significant difference was analyzed using a two-tailed student’s t test with a threshold of p values ≤ 0.05. Besides ducks, the same experiment was also carried out using chickens.
